# OCT4 cooperates with distinct ATP-dependent chromatin remodelers in naïve and primed pluripotent states in human

**DOI:** 10.1038/s41467-021-25107-3

**Published:** 2021-08-26

**Authors:** Xin Huang, Kyoung-mi Park, Paul Gontarz, Bo Zhang, Joshua Pan, Zachary McKenzie, Laura A. Fischer, Chen Dong, Sabine Dietmann, Xiaoyun Xing, Pavel V. Shliaha, Jihong Yang, Dan Li, Junjun Ding, Tenzin Lungjangwa, Maya Mitalipova, Shafqat A. Khan, Sumeth Imsoonthornruksa, Nick Jensen, Ting Wang, Cigall Kadoch, Rudolf Jaenisch, Jianlong Wang, Thorold W. Theunissen

**Affiliations:** 1 Department of Medicine, Columbia Center for Human Development, Columbia Stem Cell Initiative, Columbia University Irving Medical Center, New York, NY USA; 2 Department of Developmental Biology, Washington University School of Medicine, St. Louis, MO USA; 3 Center of Regenerative Medicine, Washington University School of Medicine, St. Louis, MO USA; 4 Department of Pediatric Oncology, Dana-Farber Cancer Institute, Boston, MA USA; 5 Broad Institute of Harvard and MIT, Cambridge, MA USA; 6 Institute of Informatics (I2), Washington University School of Medicine, St. Louis, MO USA; 7 Department of Genetics, Center for Genome Sciences & Systems Biology, Washington University School of Medicine, St. Louis, MO USA; 8 Memorial Sloan Kettering Cancer Center, New York, NY USA; 9 Department of Cell, Developmental and Regenerative Biology, Black Family Stem Cell Institute, Icahn School of Medicine at Mount Sinai, New York, NY USA; 10 Department of Cell Biology, Zhongshan School of Medicine, Sun Yat-sen University, Guangzhou, China; 11 Whitehead Institute for Biomedical Research, Cambridge, MA USA; 12 Center for Biomolecular Structure Function and Application, School of Biotechnology, Suranaree University of Technology, Nakhon Ratchasima, Thailand; 13 Department of Biology, Massachusetts Institute of Technology, Cambridge, MA USA

**Keywords:** Chromatin remodelling, Embryonic stem cells

## Abstract

Understanding the molecular underpinnings of pluripotency is a prerequisite for optimal maintenance and application of embryonic stem cells (ESCs). While the protein-protein interactions of core pluripotency factors have been identified in mouse ESCs, their interactome in human ESCs (hESCs) has not to date been explored. Here we mapped the OCT4 interactomes in naïve and primed hESCs, revealing extensive connections to mammalian ATP-dependent nucleosome remodeling complexes. In naïve hESCs, OCT4 is associated with both BRG1 and BRM, the two paralog ATPases of the BAF complex. Genome-wide location analyses and genetic studies reveal that these two enzymes cooperate in a functionally redundant manner in the transcriptional regulation of blastocyst-specific genes. In contrast, in primed hESCs, OCT4 cooperates with BRG1 and SOX2 to promote chromatin accessibility at ectodermal genes. This work reveals how a common transcription factor utilizes differential BAF complexes to control distinct transcriptional programs in naïve and primed hESCs.

## Abstract (web summary)

Although the interactors of pluripotency factors have been identified in mouse embryonic stem cells (ESCs), their interactors in human ESCs remain unexplored. Here the authors map OCT4 protein interactions in naïve and primed human ESCs to find specific interactions with BAF subunits that promote an open chromatin architecture at blastocyst-associated genes and ectodermal genes, respectively.

## Introduction

Pluripotency, the ability of a single cell to give rise to all cell types found in an organism, is a fundamental characteristic of embryonic stem cells (ESCs). Mouse and human ESCs are both derived from the inner cell mass (ICM) of the preimplantation blastocyst but differ significantly in their epigenomic, morphological, and transcriptomic features. Mouse ESCs (mESCs) are marked by early developmental properties associated with naïve pluripotency^1^. In contrast, human ESCs (hESCs) derived under conventional culture conditions are developmentally more advanced and resemble mouse epiblast stem cells (EpiSCs); thus, they are considered to represent a primed state of pluripotency^1–3^. Over the past few years, specific combinations of inhibitors and cytokines have been developed to enable the generation of naïve hESCs by resetting primed hESCs^4–6^, somatic cell reprogramming^7–10^, or deriving novel naïve hESCs directly from human preimplantation embryos^4,11^. Naïve hESCs offer a window into aspects of early development that cannot be adequately modelled with primed hESCs, such as X chromosome reactivation^12,13^ and the role of hominid-specific transposable elements associated with early embryogenesis^14,15^. Furthermore, recent studies indicate that naïve hESCs have a unique predisposition to acquire extraembryonic fates^16–20^ and generate human blastocyst-like structures^21,22^.

The POU factor OCT4, which is expressed across the pluripotency continuum in mouse and human, is considered a fundamental factor for early embryogenesis^23–26^. OCT4 is the only factor that is sufficient to trigger reprogramming of mouse and human somatic cells to induced pluripotent stem cells (iPSCs) in the absence of other factors^27,28^. Previous studies of OCT4-centered protein–protein interaction networks (i.e., the OCT4 interactomes) in mESCs have identified important links to transcriptional cofactors, epigenetic modifiers, and chromatin remodelers that synergize with OCT4 during mESC self-renewal, differentiation, and reprogramming^29–31^. While the genome-wide targets of OCT4 have been mapped in both mouse and human ESCs^32–35^, its physical interactome in hESCs has not to date been explored. Hence, it remains unclear how OCT4 controls distinct transcriptional programs in human naïve and primed pluripotent states.

Here we sought to identify critical OCT4 cofactors in human pluripotent cells. We captured the dynamic OCT4-centered protein–protein interaction networks (interactomes) under the naïve and primed conditions using affinity purification followed by mass spectrometry (AP-MS) and uncovered extensive associations with ATP-dependent chromatin remodelers. Integrative analysis generated a reference map of stage-specific transcription factors (TFs) and chromatin remodelers, linking enhancer reorganization with concordant transcriptional changes. Our work indicates that a switch in OCT4 partner association contributes to the activation of distinct target genes in naïve and primed hESCs and consequently their distinct developmental potential.

## Results

### Establishing OCT4-centered protein interactomes in naïve and primed hESCs

**Fig. 1:**
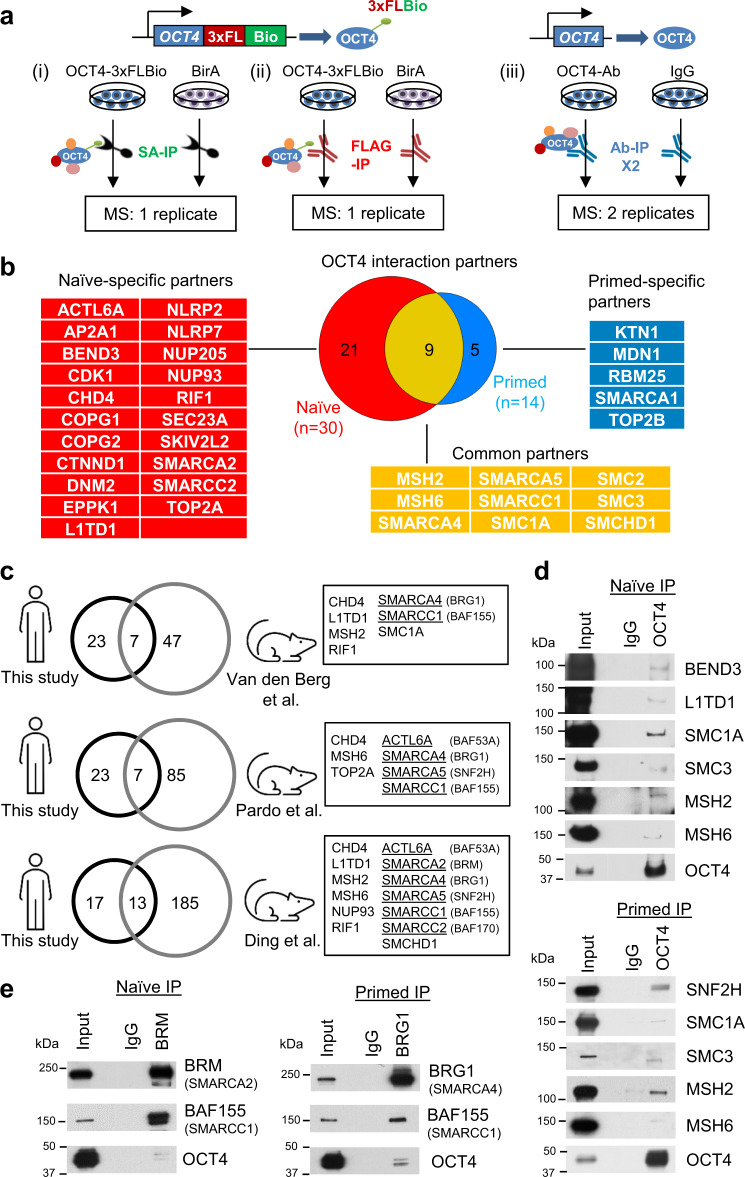
An OCT4 interactome in human embryonic stem cells (hESCs). **a** Affinity purification mass spectrometry (AP-MS) strategies for identification of OCT4-associated proteins (interactome) in hESCs. Three strategies are used: (i) streptavidin immunoprecipitation (SA-IP) of WIBR2-*OCT4*^*3x*FLB*io*^ ESCs or BirA control; (ii) FLAG-IP of WIBR2-*OCT4*^*3x*FLB*io*^ ESCs or BirA control; (iii) native OCT4 and IgG antibody (Ab-IP) pulldown in WIBR2 and WIBR3 hESC lines. **b** OCT4-associated proteins identified in naïve and primed hESCs. Proteins are listed as official gene symbols. **c** Proteins in the naïve human OCT4 interactome are compared to OCT4-associated proteins identified in three independent studies in mouse ESCs^29–31^. Components of SNF2-family chromatin remodeling complexes are underlined. **d** OCT4 co-immunoprecipitation (co-IP) followed by western blot of selected OCT4-associated proteins in naïve and primed hESCs. **e** BRM (SMARCA2) and BRG1 (SMARCA4) co-IP followed by western blot analysis of BAF155 (SMARCC1) and OCT4. **d, e** Experiment is repeated independently twice with similar results.

To identify OCT4 partners in human pluripotent cells, we employed transcription activator-like effector nucleases (TALENs) to target the bacterial ligase BirA to the *AAVS1* safe harbor locus in WIBR2 primed hESCs (Supplementary Fig. 1a–c). We then targeted a donor vector containing a fusion of the 3xFLAG tag and Biotin recognition sequence (3xFLBio) to the C-terminus of endogenous *OCT4* (Fig. 1a, Supplementary Fig. 1d, e, and see Methods for details). In the presence of BirA this sequence becomes biotinylated on the lysine residue (Supplementary Fig. 1f), enabling affinity precipitation followed by mass spectrometry (AP-MS) analysis using streptavidin (SA) beads^36^. WIBR2-BirA and WIBR2-*OCT4*^*3x*FLB*io*^ hESCs retained full expression of pluripotency genes and differentiation potential (Supplementary Fig. 1g, h). OCT4-associated proteins were identified by SA and 3xFLAG AP-MS under primed conditions and upon transfer to naïve pluripotency by overexpression of KLF2 and NANOG transgenes^4,5^ (Fig. 1a). In addition, we performed native OCT4 antibody pulldown followed by mass spectrometry in WIBR2 and WIBR3 hESCs before and after resetting using our chemically defined naïve conditions^15^ (Fig. 1a). Candidate OCT4 partners were selected based on detection in at least three of four independent MS experiments, removal of background contaminants using the CRAPome^37^ database, and spectral count analysis using a combined cumulative probability (CCP) score^38^ with a false-discovery rate (FDR) cutoff of 0.1 (Supplementary Fig. 1i, j and Supplementary Data 1). This analysis identified 30 OCT4-associated proteins in naïve hESCs and 14 in primed hESCs, 9 of which were detected in both pluripotent states (Fig. 1b). We compared the OCT4 interactome in naïve hESCs with that in mESCs^29–31^. Only 7 (23%), 7 (23%), and 13 (43%) proteins were shared between the naïve human OCT4 interactome and three independent mouse studies, respectively (Fig. 1c). While this limited overlap may hint at species-specific differences in OCT4 protein associations, it is important to bear in mind that the mouse data used in these comparisons were generated under serum/LIF conditions, which do not truly represent ground state conditions, but rather a heterogeneous and metastable state that is intermediate between naïve and primed pluripotency^39^. Components of the cohesin complex (SMC1A, SMCHD1), RNA binding protein L1TD1^40^, DNA mismatch repair proteins (MSH2, MSH6), and multiple components of SNF2-family ATP-dependent chromatin remodeling complexes (Fig. 1c, underlined) were conserved between the mouse and human OCT4 networks. Interactions of OCT4 with selected partners in naïve and primed hESCs were validated by co-immunoprecipitation (co-IP) and western blot (Fig. 1d, e). These results indicate that a subset of OCT4 interaction partners is conserved between naïve cells in mouse and human.

### OCT4-associated SNF2-family ATPases are divergently expressed in naïve and primed hESCs

**Fig. 2:**
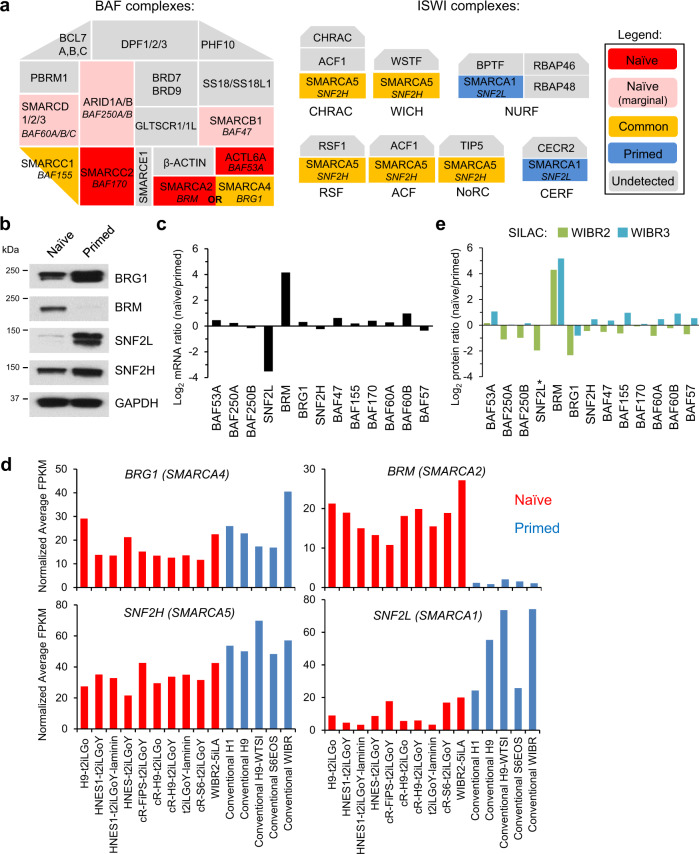
Differential expression of OCT4-associated SNF2-family ATPases in naïve and primed hESCs. **a** Illustration of naïve-specific (red/pink), primed-specific (blue), and common (yellow) components of the mammalian SWI/SNF family of subunits (represented as one complex rather than three subcomplexes cBAF, PBAF, and ncBAF) and ISWI complexes identified in the naïve and primed OCT4 interactomes. Pink color indicates proteins that are identified with marginal significance (0.1 < FDR < 0.2) in the naïve OCT4 interactome. **b** Western blot analysis of ATPase expression in naïve and primed hESCs. Experiment is repeated independently twice with similar results. **c** log_2_ ratios of mRNA expression of BAF and ISWI components in naïve and primed hESCs based on a microarray analysis^4^. **d** Expression of *BRG1 (SMARCA4), BRM (SMARCA2)*, and *SNF2L (SMARCA1)* mRNA transcripts in the alternative t2iLGoY culture condition for naïve human pluripotency (red) and parental primed hESCs (blue) according to RNA-seq data^5,46^. **e** log_2_ ratios of protein expression of BAF and ISWI components in naïve and primed hESCs based on SILAC followed by MS analysis in two naïve and primed hESC lines. *SNF2L was quantified by MS only in WIBR2 cells.

The human OCT4 interactome was strongly enriched in components of mammalian SNF2-family ATP-dependent chromatin remodeling complexes^41–45^, including members of the Brahma-associated factor (BAF or mammalian SWI/SNF) and imitation SWI (ISWI) complexes (Fig. 2a). The BAF complex contains one of two mutually exclusive ATPase subunits, BRG1 (SMARCA4) or BRM (SMARCA2), while other BAF subunits provide structural or chromatin targeting functions^45^. BRG1 was detected in both the naïve and primed OCT4 interactomes, while its paralog ATPase, BRM was only detected in the naïve OCT4 interactome (Fig. 1b, Fig. 2a, and Supplementary Fig. 2a). In a similar fashion, ISWI complexes contain one of two ATPase subunits, SNF2H (SMARCA5) or SNF2L (SMARCA1), and 2–4 additional subunits^45^. SNF2H was detected in both the naïve and primed OCT4 interactomes, while SNF2L was only detected in the primed OCT4 interactome (Fig. 1b and Fig. 2a). Hence, OCT4 is associated with distinct SNF2-family ATPases in naïve and primed hESCs.

We examined whether these distinct protein interactions may be explained by differential expression of OCT4 partners. Indeed, BRM and SNF2L were specifically expressed in naïve and primed hESCs, respectively (Fig. 2b, c). Other BAF and ISWI components were expressed at similar levels in naïve and primed hESCs (Fig. 2b, c and Supplementary Fig. 2b), although BRG1 was elevated at the protein level in primed cells (Fig. 2b). Similar expression patterns were observed in naïve hESCs generated using t2i/L/Gö, an alternative culture formulation for naïve hESCs^5,46^ (Fig. 2d). Some BAF subunits that were identified in the naïve OCT4 interactome nonetheless showed an interaction with OCT4 by co-IP assay in both naïve and primed cells (Supplementary Fig. 2c), suggesting that the stringent cutoffs used in our AP-MS analysis may have caused some bona fide interactions to be omitted. Similarly, the topoisomerase TOP2A, which synergizes with the BAF complex to recruit pluripotency factors in mouse ESCs^47^, was identified in the naïve OCT4 interactome but its CCP score was only slightly below the cutoff for inclusion in the primed OCT4 interactome (Supplementary Fig. 1j). Conversely, its isoform TOP2B was upregulated in primed cells and exclusively detected in the primed OCT4 interactome (Supplementary Fig. 1j, k). Co-IP assays indicated that SNF2L and TOP2B form a direct interaction with BRG1 in primed hESCs (Supplementary Fig. 2d). These data suggest that some of the protein associations with OCT4 may be bridged by third partners, such as BRG1.

To determine whether other OCT4-associated proteins were differentially expressed between naïve and primed hESCs, we performed stable isotope labeling with amino acids in cell culture (SILAC) followed by MS to determine global protein expression levels (Supplementary Fig. 2e). This analysis confirmed that BRG1 was upregulated at the protein level in primed hESCs, whereas several naïve-specific OCT4 partners (BRM, EPPK1, and NLRP2/7) were highly induced in naïve hESCs (Fig. 2e and Supplementary Fig. 2f). However, most OCT4-associated proteins did not show a significant shift in expression between naïve and primed hESCs. We conclude that cell-type-specific association with OCT4 can be attributed to differential protein expression in only a handful of cases, including BRM (naïve specific) and SNF2L (primed specific).

### OCT4 and BAF complexes shape the naïve- and primed-specific chromatin landscapes

**Fig. 3:**
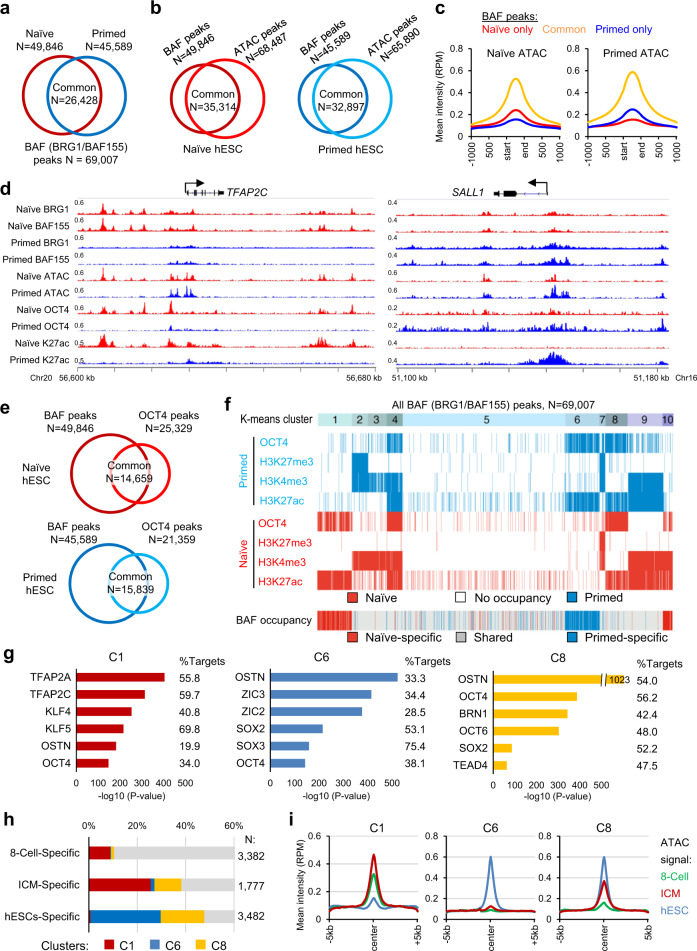
Genome-wide binding profiles of OCT4 and BAF complexes shape the naïve- and primed-specific enhancer landscapes. **a** Overlap of BAF (BRG1/BAF155) peaks in naïve and primed hESCs identified by ChIP-seq analysis. **b** Overlap of BAF and chromatin accessibility (ATAC-seq)^48^ peaks in naïve (left) and primed (right) hESCs. **c** Intensity plots of mean ATAC signals at naïve-only, primed-only, and common BAF peaks (shown in panel **a**) in naïve (left) and primed (right) hESCs. **d** ChIP-seq tracks of BAF155, BRG1, ATAC-seq, OCT4, and H3K27ac at the naïve-specific *TFAP2C* and primed-specific *SALL1* loci. The two tracks of each factor between naïve and primed hESCs are normalized to the same RPM (reads per million) values. **e** Overlap of BAF (BRG1/BAF155) and OCT4 peaks in naïve (top) and primed (bottom) hESCs. **f** K-means clustering analysis of all BAF (*N* = 69,007) peaks (shown in panel **a**) in naïve and primed hESCs identified 10 clusters with distinct patterns of OCT4 and histone marks enrichment. **g** Highly enriched TF binding motifs in regions C1 (left), C6 (middle), and C10 (right) from clustering analysis (shown in panel **f**). OSTN: OCT4/SOX2/TCF/NANOG consensus motif^80^. **h**–**i** Overlap of 8-cell-specific, ICM-specific, and primed hESC-specific ATAC-seq peaks (**h**) and plots of mean ATAC intensity (**i**) at clusters C1, C6, and C8 peak regions. The stage-specific distal ATAC regions are from a recent study of chromatin accessibility in human preimplantation embryos^53^.

To explore the role of the BAF complexes in regulating naïve versus primed human pluripotency, we performed chromatin immunoprecipitation followed by deep sequencing (ChIP-seq) analysis using antibodies specific for BRG1 and BAF155 (SMARCC1, a common scaffold protein of the BAF complex^42^) under naïve and primed conditions (ChIP-seq QC metrics in Supplementary Data 2 and source data provided with this paper). A total of 69,007 BAF (BRG1/BAF155 merged) peaks were identified in both states, among which 26,428 peaks (53.0%, 26,428/49,846 of naïve peaks, or 58.0%, 26,428/45,589 of primed peaks) were shared (Fig. 3a). Consistent with the known role of BAF complexes in promoting an accessible chromatin architecture, we observed a high overlap of BAF peaks with open chromatin regions in naïve and primed cells, as defined by the assay for transposase-accessible chromatin followed by sequencing (ATAC-seq)^48^ (Fig. 3b). BAF peaks specific to naïve and primed cells showed higher ATAC intensities in their respective states, as exemplified by genes active in naïve (*TFAP2C*) and primed (*SALL1*) states (Fig. 3c, d).

In line with the physical interaction between OCT4 and the BAF components, OCT4 peak regions from ChIP-seq^35^ overlapped substantially (57.9%, 14,659/25,329 in naïve state or 74.1%, 15,839/21,359 in primed state) with BAF peaks (Fig. 3e). Using a clustering method based on the ChIP-seq intensities of OCT4^35^ and histone H3 modification marks K4me3^4^, K27ac^35^, and K27me3^4^ at all BAF peak regions (*N* = 69,007), we defined 10 discrete BAF-bound clusters with distinct occupancy patterns of OCT4 and active and repressive histone marks in naïve and primed hESCs (Fig. 3f, Supplementary Fig. 3a, b, and Supplementary Data 3). Of particular interest for understanding differential gene regulation, clusters 1, 6, and 8 (C1, C6, C8) represent naïve-specific, primed-specific, and shared enhancers, respectively, that were enriched in OCT4 and the active histone mark H3K27ac but lacked the promoter mark H3K4me3 and the repressive mark H3K27me3. Additionally, clusters 4 and 10 (C4, C10) represent shared and naïve-specific active promoters, respectively, that were enriched in OCT4, H3K27ac, and H3K4me3, but lacked H3K27me3, while cluster 7 (C7) represents shared bivalent promoters that were enriched in both H3K4me3 and H3K27me3 (Fig. 3f and Supplementary Fig. 3a, b). As expected, annotations of these peak regions agreed with their predicted activity in naïve vs. primed hESCs based on chromatin accessibility and expression of the nearest genes (Supplementary Fig. 3c, d).

By examining the presence of TF binding motifs, we identified enrichment of TFAP2A/C and KLF4/5 motifs at naïve-specific OCT4/BAF-bound enhancers (C1) and promoters (C10) (Fig. 3g and Supplementary Fig. 3e). KLF4 and TFAP2C were previously shown to activate enhancers specific to naïve hESCs^14,48^. In contrast, primed-specific OCT4/BAF-bound enhancers (C6) were enriched in motifs associated with ZIC2/3 and SOX2/3 (Fig. 3g). Zic2/3 have been implicated as transcriptional determinants of primed mouse EpiSCs^49,50^, while SOX2 is a master regulator of pluripotent cells that also contributes to neural differentiation^51,52^. These findings suggest that the BAF complex is recruited to discrete sets of target genes in naïve and primed hESCs through association with TFs that are differentially expressed in these two pluripotent states.

To determine the in vivo relevance of these OCT4/BAF-bound enhancers, we examined stage-specific enhancers that were identified in early human embryos using ATAC-seq analysis^53^. Distal ATAC-seq peaks that were identified specifically in human 8-cell embryos, ICM, and primed hESCs were compared to naïve-specific (C1), primed-specific (C6), and naïve/primed-shared (C8) enhancer regions. C1 regions were associated with 8-cell and ICM-specific ATAC peaks, while C8 regions were only associated with ICM-specific ATAC peaks in vivo (Fig. 3h, i). These data confirm the biological relevance of the identified OCT4/BAF-bound enhancers and demonstrate that naïve hESCs partially recapitulate enhancer signatures of human 8-cell-to-ICM embryos^10^.

### Naïve and primed-specific BAF-bound enhancers harbor distinct chromatin remodelers and TFs

Our OCT4 interactome studies identified BRM as a naïve-specific partner (Fig. 1b). ChIP-seq analysis identified 17,413 BRM peaks in naïve hESCs, most of which (88.8%, 15,463/17,413) were shared with BAF (BRG1/BAF155) peaks in naïve cells (Supplementary Fig. 4a). Previous studies suggested that the BRM- and BRG1-containing BAF complexes are mutually exclusive^54,55^. In naïve hESCs, we detected an interaction between these two ATPases by reciprocal co-IP experiments (Supplementary Fig. 4b). However, the interaction of BRM with BRG1, but not other BAF components (BAF53A, BAF47), was abolished using a more stringent co-IP condition (Supplementary Fig. 4c). Hence, the two ATPases are likely not assembled in the same BAF complex, which is consistent with structural studies^42,56^, and their association in naïve hESCs may be bridged by a third interactor, such as OCT4.

**Fig. 4:**
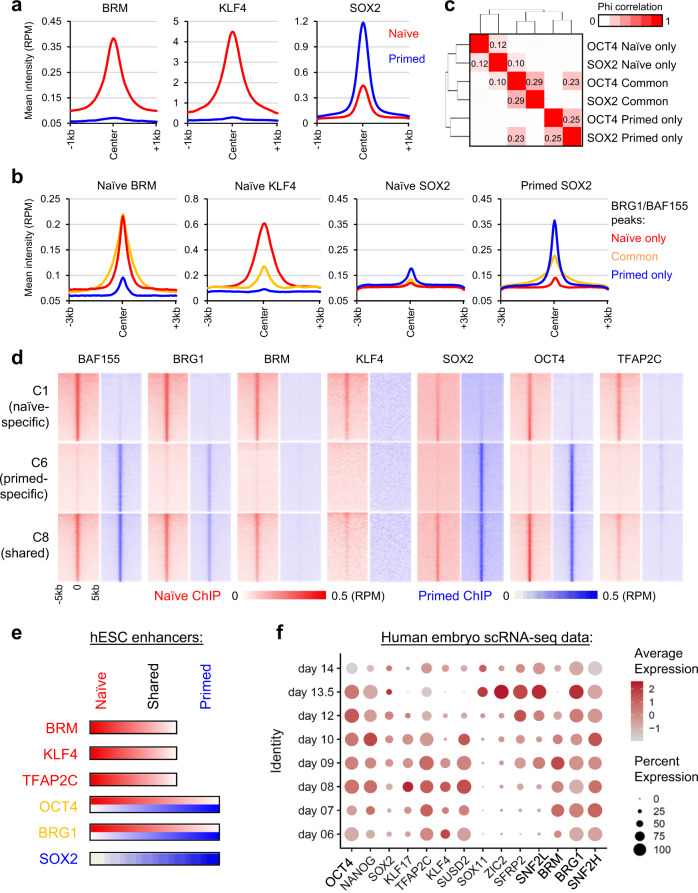
Naïve and primed-specific enhancers harbor distinct chromatin remodelers and TFs. **a** Mean intensity plots of BRM, SOX2 (ChIP-seq), and KLF4 (CUT&Tag) signals in naïve and primed hESCs. **b** Mean intensity plots of BRM, KLF4, and SOX2 signals at naïve-only, primed-only, and naïve/primed-common BAF peaks. **c** Correlation analysis of OCT4 and SOX2 chromatin association in naïve and primed hESCs grouped by naïve-only, primed-only, and naïve/primed-common OCT4 or SOX2 peaks. **d** Intensity heatmaps of BAF subunits, KLF4, SOX2, OCT4, and TFAP2C signals at clusters C1, C6, and C8 regions in naïve (left) and primed (right) hESCs. **e** Summary of activities of chromatin remodeling complexes and TFs in naïve (C1), primed (C6), and shared (C8) enhancers in hESCs. **f** Expression of OCT4-associated chromatin remodelers and TFs in single-cell RNA-seq analysis of human embryos cultured in vitro between days 6 and 14 of human development^57^. Average gene expression levels and the percentage of cells that express each gene are presented with differential color intensities and circle sizes, respectively. Note that *KLF17* and *SUSD2* are markers associated with naïve pluripotency, while *SFRP2* and *SOX11* are associated with primed pluripotency.

The enrichment of the KLF4 motif at naïve-specific (C1) enhancers and of the SOX2 motif at primed-specific (C6) enhancers (Fig. 3g) prompted us to further investigate the genome-wide location of KLF4 and SOX2 in naïve and primed hESCs by CUT&Tag and ChIP-seq analysis, respectively (Supplementary Data 2). Interestingly, expression of KLF4 and SOX2 was largely naïve and primed specific, respectively (Supplementary Fig. 4d, e). Accordingly, association of KLF4 to chromatin was only detected in naïve hESCs, while that of SOX2 was detected with a much larger proportion in primed than naive hESCs (Fig. 4a and Supplementary Fig. 4f). In naïve hESCs, BRM and KLF4 were enriched at naïve specific and common BAF (BRG1/BAF155) peaks, while SOX2 occupied all BAF peaks with a low intensity. In contrast, SOX2 was more enriched at primed specific and common BAF regions in primed hESCs (Fig. 4b). These data suggest that SOX2 may function as a largely primed-specific TF in the human context, although its genome-wide binding in naïve hESCs was still weakly correlated with that of OCT4 (Fig. 4c). We then examined the association of state-specific chromatin remodelers and TFs with BAF-bound enhancers. BRG1, BRM, KLF4, OCT4, and TFAP2C^48^ were enriched at naïve-specific (C1) and naïve/primed-shared (C8) enhancers, while BRG1, SOX2, and OCT4 were enriched at primed-specific (C6) and naïve/primed-shared (C8) enhancers (Fig. 4d and Supplementary Fig. 4g). Thus, the naïve and primed-specific enhancers harbor distinct ATP-dependent chromatin remodelers and TFs (Fig. 4e).

To investigate whether such a switch in OCT4 partner association could also be observed in transcriptome studies of human embryos, we analyzed single-cell RNA-seq (scRNA-seq) data from human embryos between days 6 and 14 of development^57^. By profiling cells corresponding to the epiblast (EPI) lineage over this period, we observed elevated expression of the naïve-associated factors *BRM*, *KLF4*, and *TFAP2C* during early timepoints, which diminished after day 9 (Fig. 4f). The primed-specific OCT4 partner *SNF2L* was induced starting at day 9 together with *SOX2* and subsequently, *ZIC2*. In contrast, *OCT4, BRG1*, and *SNF2H* maintained a relatively constant expression level over this time course (Fig. 4f). These transcriptional dynamics are consistent with the dynamic reorganization of OCT4 protein partners observed in naïve and primed hESCs in vitro.

### Naïve- and primed-specific BAF-bound enhancers drive expression of blastocyst and ectodermal signatures, respectively

**Fig. 5:**
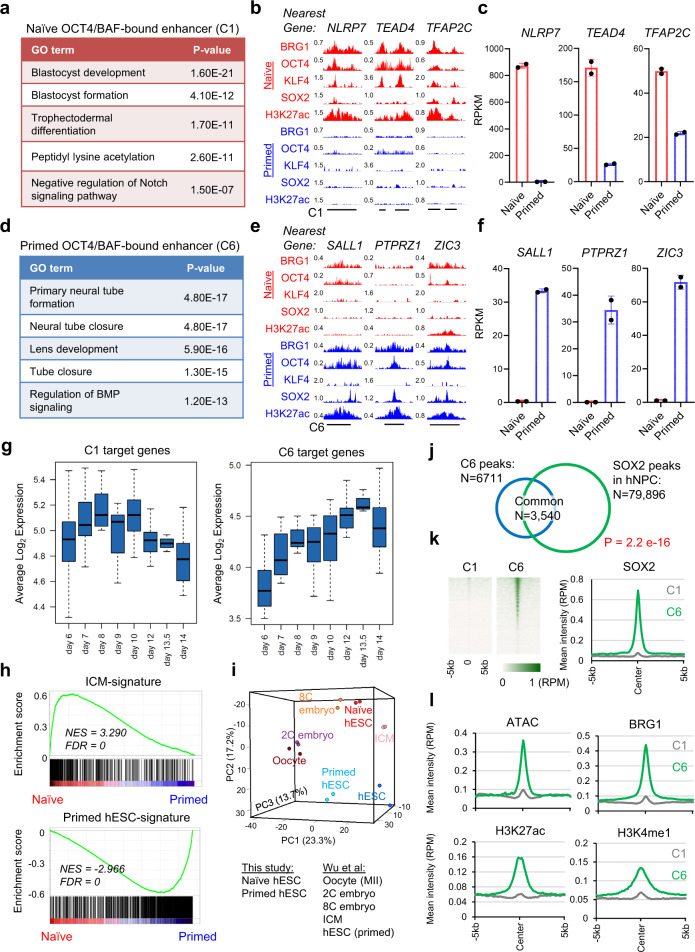
Naïve and primed-specific enhancers drive expression of blastocyst and ectodermal lineage signatures, respectively. **a**–**c** Gene ontology (GO) analysis (**a**), ChIP-seq tracks (**b**), and expression from RNA-seq analysis (**c**) of representative target genes (*NLRP7*, *TEAD4*, and *TFAP2C*) located nearby naïve-specific OCT4/BAF-bound enhancers (C1). **d–f** GO analysis (**d**), ChIP-seq tracks (**e**), and expression from RNA-seq analysis (**f**) of typical target genes (*SALL1*, *PTPRZ1*, and *ZIC3*) located nearby primed-specific OCT4/BAF-bound enhancers (C6). **a, d** *P* value is from the right-sided Fisher’s Extract test. **c, f** Data are presented as mean ± SD, obtained from *n* = 2 biologically independent experiments. **g** Expression of C1 and C6 target genes in a scRNA-seq analysis of epiblast cells in human embryos cultured in a 3D matrix between days 6 and 14 of development^57^. Boxplot presents the 25th, median, and 75th quartiles, and the whiskers extend 1.5 of interquartile ranges. **h** Geneset Enrichment Analysis (GSEA) for the terms “ICM-signature” and “Primed hESC-signature” in naïve versus primed hESCs used in this study. These signature genes were defined in a transcriptome study of human preimplantation embryos^53^. **i** Principal component analysis (PCA) of RNA-seq data from naïve and primed hESCs (this study) and distinct stages of human preimplantation development^53^. **j** Overlap of SOX2 peaks in human neural progenitor cells (hNPC)^52^ and primed-specific enhancer (C6) regions in hESCs. *P* value is from the right-sided Fisher’s Extract test. **k** Heatmaps and intensity plots of SOX2 ChIP-seq in hNPCs^52^ at naïve- (C1) and primed-specific (C6) enhancer regions. **l** Intensity plots of ATAC-seq, BRG1, and enhancer marks H3K27ac and H3K4me1 in hNPCs^54^ at naïve- (C1) and primed-specific (C6) enhancer regions.

We examined the identity of the target genes of the OCT4/BAF-bound enhancers in naïve and primed hESCs. Naïve-specific OCT4/BAF-bound enhancers (C1) were located nearby genes that are involved in blastocyst development, blastocyst formation, and trophectoderm differentiation. Examples of such genes include *NLRP7, TEAD4*, and *TFAP2C* (Fig. 5a–c). In contrast, primed-specific OCT4/BAF-bound enhancers (C6) were located nearby genes that are involved in differentiation towards ectoderm (Fig. 5d), which is consistent with recent evidence in the mouse that ectodermal enhancers become primed in the early post-implantation epiblast^58^. Examples of such genes include *SALL1, PTPRZ1*, and *ZIC3* (Fig. 5e, f). As expected, naïve/primed-shared OCT4/BAF-bound enhancers (C8) were located nearby genes that are involved in stem cell population maintenance (Supplementary Fig. 5a, b). Comparison with scRNA-seq data from human embryos^57^ confirmed that naïve-specific enhancer target genes (C1) were more highly expressed during early timepoints, whereas primed-specific enhancer target genes (C6) were more highly expressed after day 9 (Fig. 5g and Supplementary Data 3). Similar expression dynamics were observed in non-human primate embryos^59^ (Supplementary Fig. 5c). In accordance with prior studies that examined the developmental identity of naïve hESCs^46,60^, naïve hESCs displayed a human ICM-specific gene expression signature^53^ (Fig. 5h), which was confirmed by principle component analysis (PCA) (Fig. 5i). These data indicate that OCT4/BAF-bound enhancers drive the expression of blastocyst-specific genes in naïve hESCs.

Since the primed-specific OCT4/BAF-bound enhancers (C6) were associated with genes involved in ectoderm development, we investigated the activity of these enhancers in human neural progenitor cells (hNPCs). Primed-specific OCT4/BAF-bound enhancers overlapped substantially with SOX2 peaks in hNPCs^52^ (Fig. 5j, k). Chromatin accessibility, BRG1 binding, and the enhancer-associated histone marks H3K27ac and H3K4me1 were all highly enriched at primed-specific enhancers (C6) compared to naïve-specific enhancers (C1) in hNPCs^54^ (Fig. 5l and Supplementary Fig. 5e). In line with activity of these enhancers, expression of primed-specific enhancer target genes was significantly higher in primed hESCs and hNPCs compared to naïve hESCs (Supplementary Fig. 5d). In addition, we putatively identified a naïve-specific (C1, distance to TSS: 50 kb) and a primed-specific (C6, distance to TSS: 45 kb) enhancer within downstream intergenic regions of *SOX2*. BRG1 and OCT4 co-occupied the C1 enhancer in naïve hESCs, whereas BRG1 and OCT4/SOX2 co-occupied the C6 enhancer in primed hESCs, and finally BRG1 and SOX2 remained present at the C6 enhancer in hNPCs (Supplementary Fig. 5f). These data reveal a regulatory cascade at distinct enhancers controlled by stage-specific TFs and BAF chromatin remodelers to drive expression of SOX2 in hESCs and hNPCs.

### Functional redundancy of BRG1 and BRM in naïve hESCs

**Fig. 6:**
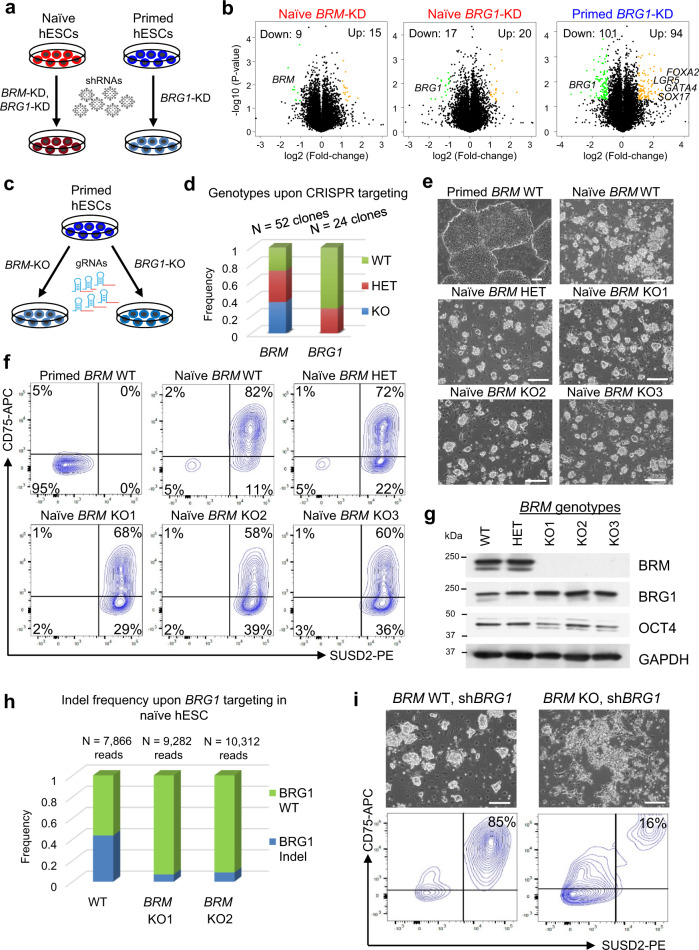
Functional redundancy of BRG1 and BRM in naïve hESCs. **a** Schematic depicting the generation of *BRM* or *BRG1* knockdown (KD) in both naïve (red) and primed (blue) hESCs. **b** Volcano plots of significantly dysregulated genes (fold change > 2, *p* value < 0.05, from unpaired two-sided *t*-test) in shBRM vs. control KD in naïve hESCs, shBRG1 vs. control KD in naïve hESCs, and shBRG1 vs. control KD in primed hESCs. **c** Schematic depicting the generation of *BRM* or *BRG1* knockout (KO) clones by CRISPR/Cas9 targeting of each ATPase in primed hESCs. **d** Genotypes of primed hESC lines obtained after CRISPR targeting of *BRM* (*n* = 52 clones) or *BRG1* (*n* = 24 clones) ATPase domain. Note that we were unable to derive primed hESC lines containing homozygous frameshift mutations in *BRG1*. **e** Phase contrast images of cell lines analyzed in primed hESCs and naïve hESCs with three distinct *BRM* genotypes: wild-type (*BRM*^*+/+*^), heterozygous (*BRM*^+/−^), or homozygous (*BRM*^−/−^). Scale bar is 200 μm. **f** Flow cytometry analysis for the naïve cell-surface markers CD75 and SUSD2 in the previous panel. *n* = 3 independent BRG1 KO clones were analyzed. **g** Western blot analysis for BRM, BRG1, OCT4, and GAPDH in *BRM* WT, HET, and KO (three independent clones) naïve hESCs. **h** Frequency of *BRG1* insertion–deletions (indels) after introduction of sgRNAs targeting the *BRG1* ATPase domain in WT naïve hESCs and two independent *BRM* KO clones. Numbers of reads sequenced in each cell line are indicated at the top of the histogram. **i** *BRG1* KD in *BRM*^−/−^ naïve hESCs impairs self-renewal as assessed by morphological changes and reduced expression of the naïve cell-surface markers CD75 and SUSD2. This experiment is representative of *n* = 2 biological replicates. Scale bar is 200 μm.

We examined the functional significance of the BAF complex in controlling naïve and primed human pluripotency. We performed short hairpin RNA (shRNA)-mediated knockdown (KD) of BRG1 in naïve and primed hESCs, and KD of BRM in naïve hESCs (Fig. 6a). Knockdown resulted in more than 50% reduction in mRNA transcript and protein levels of each target gene (Supplementary Fig. 6a, b). Approximately two hundred genes (101 Down, 94 Up, fold change > 2, *p* value < 0.05) were differentially expressed upon BRG1 KD in primed hESCs, but little effect was seen in naïve hESCs upon depletion of either BAF ATPase (Fig. 6b and Supplementary Data 4). In agreement with prior findings^61^, BRG1 knockdown in primed hESCs caused upregulation of genes with bivalent chromatin domains, as identified in C7 of our clustering analysis (Fig. 3f and Supplementary Fig. 3b). In particular, regulators of endodermal lineage differentiation, such as *SOX17, GATA4, FOXA2*, and *LGR5*, were significantly upregulated in response to BRG1 depletion in primed hESCs (Fig. 6b and Supplementary Fig. 6c–e).

Since KD of neither ATPase had a significant effect on transcription in naïve hESCs, we examined whether BRG1 and BRM may have an overlapping functional role in naïve human pluripotency. We first introduced guide RNA (gRNAs) targeting the ATPase domains of BRG1 or BRM into primed hESCs and genotyped individual clones (Fig. 6c). One third of clones nucleofected with *BRG1* gRNAs contained heterozygous frameshift mutations in *BRG1*, but none contained homozygous frameshift mutations (Fig. 6d). In contrast, nucleofection with *BRM* gRNAs generated equivalent proportions of clones that were wild-type (WT), heterozygous (HET), or knockout (KO) for *BRM* (Fig. 6d). These results confirm that BRG1, but not BRM, is critical for self-renewal of primed hESCs. We then reprogrammed these primed *BRM*^*+/+*^*, BRM*^+/−^, or *BRM*^−/−^ hESCs to the naïve state and found that cells of all three genotypes expressed the naïve cell-surface markers CD75 and SUSD2^62,63^, although CD75 intensity was marginally reduced in the absence of BRM (Fig. 6e, f and Supplementary Fig. 6f). Western blot analysis indicated that BRG1 protein levels increased in the absence of BRM, suggesting a potential compensatory mechanism (Fig. 6g). We then attempted to deplete BRG1 in naïve hESCs that were also deficient in BRM. gRNAs targeting the ATPase domain of BRG1 were introduced into *WT* and two independent clones of *BRM*^−/−^ naïve hESCs. The frequency of insertion–deletions (indels) in each genetic background was determined by sequencing. While we detected a substantial (43.4%) frequency of *BRG1* alleles containing indels in *WT* naïve cells, very few (<9%) *BRG1* indels were detected in the *BRM*^−/−^ background (Fig. 6h). In addition, KD of BRG1 in *BRM*^−/−^ naïve cells resulted in severe collapse of colony morphology and loss of the CD75^+^/SUSD2^+^ population compared to KD of BRG1 in WT naïve cells (Fig. 6i). These results indicate that naïve cells deficient in both BAF ATPases are selectively eliminated during culture.

**Fig. 7:**
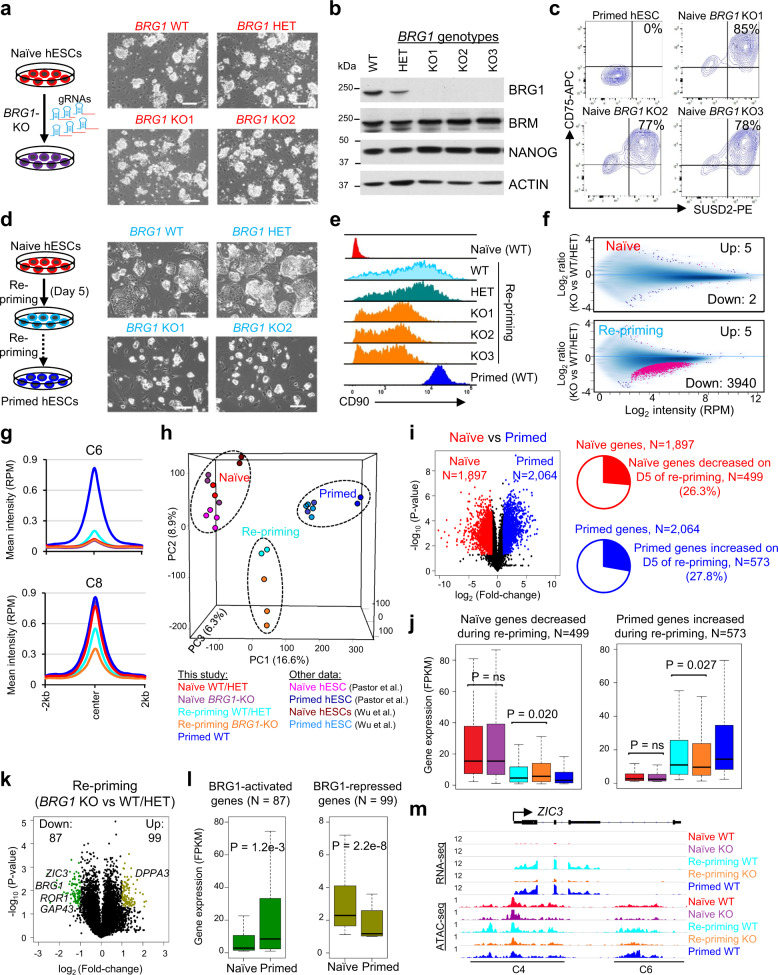
BRG1 regulates chromatin accessibility during the exit from naïve pluripotency. **a** Schematic overview of CRISPR/Cas9-mediated generation of BRG1 KO naïve hESCs and representative images. *n* = 3 independent BRG1 KO clones were generated. Scale bar is 200 μm. **b** Western blot analysis for BRG1, BRM, NANOG, and ACTIN in WT (*BRG1*^*+/+*^), HET (*BRG1*^+/−^), and KO (*BRG1*^*−*/*−*^) naïve hESCs (three independent clones). **c** Flow cytometry analysis for the naïve cell-surface markers CD75 and SUSD2 in primed hESCs and naïve *BRG1* KO hESCs. *n* = 3 independent BRG1 KO clones were analyzed. **d** Schematic overview of the naïve-to-primed pluripotency transition and representative images with WT, *BRG1*^*+/−*^, and *BRG1*^−/−^ cells on day 5 of repriming. Scale bar is 200 μm. **e** Histogram representing the mean fluorescence intensity (MFI) of CD90 cell-surface antibody staining in *BRG1* WT, HET, and KO cells on d10 of repriming compared to naïve and primed hESCs. *n* = 3 independent BRG1 KO clones were analyzed. **f** Scatterplots showing the distribution of ATAC-seq peaks in *BRG1* KO vs. WT/HET cells under naïve conditions (top) or on day 4 of repriming (bottom). Sites with significantly different ATAC intensities were determined by FDR < 0.05. **g** Mean ATAC-seq intensity at primed-specific (C6) and naïve-primed-shared (C8) enhancers of the BAF peaks during repriming in *BRG1* KO vs. WT/HET cells. **h** Principal component analysis (PCA) of ATAC-seq profiles from *BRG1* WT, HET, and KO cells under naïve conditions and during repriming compared to published ATAC-seq data of naïve and primed hESCs^48,53^. **i** Volcano plot (left) showing differentially expressed genes (fold change > 2, *p* value < 0.05, from unpaired two-sided *t*-test) between naïve and primed (WT) hESCs. Pie charts (right) for portions of naïve genes with decreased expression and primed genes with increased expression on day 5 repriming, compared with day 0 of naive cells. **j** Expression of the subsets of naïve genes decreased in repriming (left) and primed genes increased in repriming (right) from RNA-seq analysis of *BRG1* WT/HET and KO cells under naïve, primed conditions, and during repriming. **i**, **j** Data are obtained from *n* = 3 biologically independent experiments. **k** Volcano plot showing differentially expressed genes between *BRG1* KO and WT/HET naïve hESCs on day 5 of repriming. **l** Expression of BRG1-activated (*N* = 87) and -repressed (*N* = 99) genes on day 5 of repriming in naïve and primed hESCs. **m** RNA-seq and ATAC-seq tracks at the *ZIC3* locus showing reduced expression and chromatin accessibility at enhancer and promoter regions in *BRG1*^−/−^ cells during repriming. Boxplots (**j, l**) present the 25th, median, and 75th quartiles, and the whiskers extend to the 1.5 of interquartile ranges. *P* value is from two-sided Mann–Whitney test.

Based on the above findings we predicted that it should be possible to completely disrupt BRG1 in naïve hESCs that contain functional BRM. We subcloned *BRM*^*+/+*^ naïve cells that were transfected with gRNAs targeting *BRG1* and isolated three clones containing homozygous mutations in the *BRG1* ATPase domain (Fig. 7a). These cells lacked detectable BRG1 protein expression (Fig. 7b) but maintained expression of CD75 and SUSD2 (Fig. 7c). Furthermore, RNA-seq analysis indicated few differentially expressed genes (DEGs) between *WT*, *BRM*^−/−^, and *BRG1*^−/−^ naïve hESCs (Supplementary Fig. 7a). We surmise that neither BRM nor BRG1 is required for maintenance of naïve hESCs, which supports our hypothesis that these two ATPases play a functionally redundant role in naïve human pluripotency.

### BRG1 regulates chromatin accessibility during the exit from naïve pluripotency

Since we were unable to derive *BRG1*^−/−^ primed hESCs (Fig. 6d), we hypothesized that BRG1 is essential for establishment of primed pluripotency. Naïve hESCs containing different *BRG1* genotypes were treated with primed media, a process called “re-priming” (Fig. 7d). *WT* and *BRG1*^+/−^ naïve hESCs rapidly acquired a flattened epithelioid morphology typical of primed hESCs within 5 days of repriming. In contrast, *BRG1*^−/−^ naïve hESCs initially retained a dome-shaped colony morphology (Fig. 7d). In agreement with this delayed morphological transition, *WT* and *BRG1*^+/−^ naïve hESCs showed more rapid induction of the primed-specific cell-surface marker CD90 (Fig. 7e). ATAC-seq analysis revealed few differentially accessible regions (DARs, FDR < 0.05) between *WT*, *BRG1*^+/−^, or *BRG1*^−/−^ cells under naïve conditions (Fig. 7f). In contrast, chromatin accessibility was substantially reduced (3940 out of 3945, or 99.9% DARs) in *BRG1*^−/−^ cells compared to *WT*/*BRG1*^+/−^ cells during repriming (Fig. 7f). These reduced DARs (*N* = 3940) were enriched in the primed specific (C6) and naïve/primed-shared (C8) OCT4/BAF-bound enhancers identified in our ChIP-seq analysis (Fig. 7g and Supplementary Fig. 7b). The C6 enhancers with reduced chromatin accessibility were associated with target genes that are upregulated in both primed hESCs and hNPCs (Supplementary Fig. 7c). Hence, the removal of BRG1 alters the epigenomic landscape during the naïve-to-primed transition (Fig. 7h and Supplementary Fig. 7d).

Finally, we performed RNA-seq analysis on *WT*, *BRG1*^+/−^, and *BRG1*^−/−^ hESCs during 5 days of repriming. *BRM* transcript was rapidly downregulated, while *BRG1* levels were maintained in WT cells (Supplementary Fig. 7e). There were 1897 and 2064 DEGs significantly (fold change > 2, *p* value < 0.05) upregulated in WT naïve and primed cells, respectively (Fig. 7i and Supplementary Data 5). Clustering analysis based on these DEGs indicated that cells undergoing repriming acquired an intermediate transcriptional identity (Supplementary Fig. 7f). 499 (out of 1897, 26.3%) and 573 (out of 2064, 27.8%) of these DEGs were significantly down and upregulated, respectively, during repriming compared with the naïve state (Fig. 7i). Consistent with the retention of a naïve colony morphology, the reduction of naïve-specific genes and activation of primed-specific genes were delayed in *BRG1*^−/−^ cells (Fig. 7j and Supplementary Fig. 7g). Examples of genes showing delayed repriming kinetics in *BRG1*^−/−^ cells include the naïve-specific TF *DPPA3* and the primed-specific genes *GAP43*, *ROR1*, and *ZIC3*, which also exhibited reduced chromatin accessibility at enhancer and/or promoter regions (Fig. 7k–m and Supplementary Fig. 7h). Hence, while BRG1 appears to be dispensable for maintenance of naïve hESCs, it plays a critical role in ensuring adequate chromatin accessibility during the exit from naïve pluripotency.

## Discussion

**Fig. 8:**
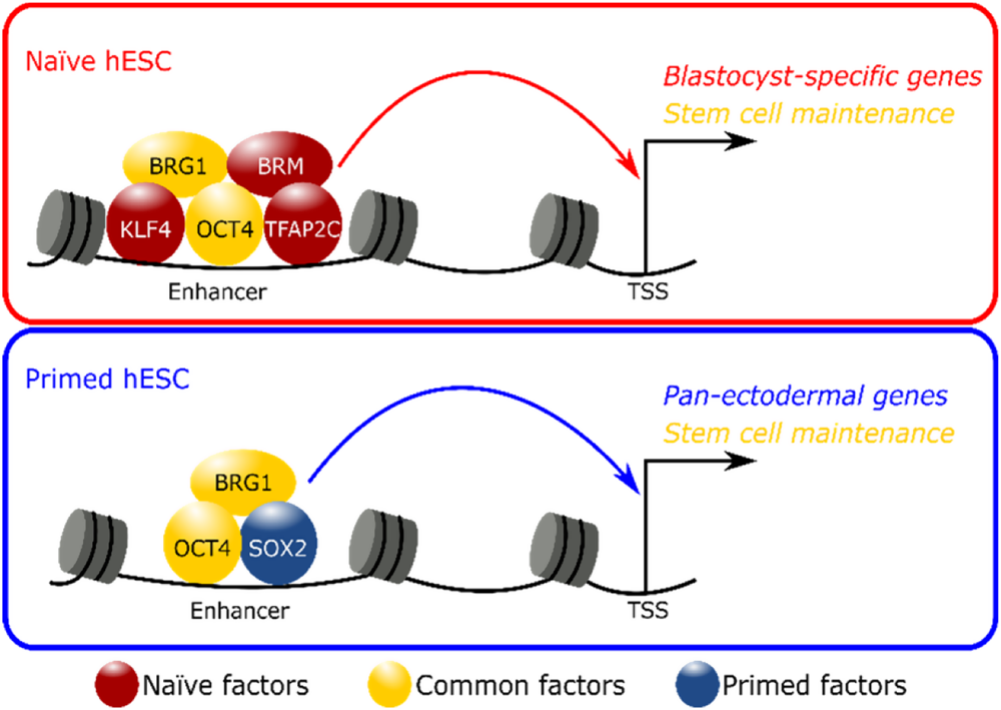
Model of enhancer regulation by common (OCT4, BRG1) and state-specific TFs and chromatin remodelers in naïve and primed hESCs. In naïve hESCs OCT4 regulates enhancers of blastocyst-specific and stem cell maintenance genes together with the BAF ATPases BRG1 and BRM. Specificity of OCT4/BAF targeting to these naïve-specific genomic sites is conferred by the naïve TFs TFAP2C and KLF4, which are downregulated during the naïve-to-primed transition in hESCs and in human embryos cultured through implantation stages in vitro^57^. In contrast, in primed hESCs OCT4 and SOX2 control expression of pan-ectodermal lineage and stem cell maintenance genes together with the BAF ATPase BRG1. Hence, the BAF complex has overlapping and divergent roles in naïve and primed pluripotent states in human.

In summary, we have generated an interactome of protein–protein interactions around OCT4 in primed hESCs and then reconstructed this interactome under recently devised conditions for naïve human pluripotency. Our results indicate that OCT4 engages in dynamic interactions with ATP-dependent chromatin remodelers in human pluripotent states. While the interaction between OCT4 and BRG1 has been well documented in mouse ESCs^44,64^, its association with distinct chromatin remodelers under naïve and primed conditions was not previously reported. The currently prevailing view is that BRG1 is the key catalytic subunit of BAF complexes in ESCs^64^ and early embryos^65,66^, whereas BRM is thought to play a greater role in differentiated cells that are less proliferative^67^. By assessing the expression, genome-wide binding, and function of BAF subunits in naïve and primed hESCs, we propose that both BRG1 and BRM contribute to the activation of enhancers involved in blastocyst development and trophectoderm specification in naïve cells. Co-binding of KLF4 and TFAP2C to OCT4/BAF-bound enhancers suggests that these two TFs provide specificity to OCT4 binding in the naïve state, as was previously reported for KLF4 during the early stages of human fibroblast reprogramming^14^. In contrast, OCT4 engages with the BRG1-containing BAF complex and SOX2 to activate ectodermal enhancers in primed hESCs. This precocious activation of ectodermal enhancers in primed hESCs is consistent with recent evidence from mouse embryos that ectodermal enhancers become primed in the early post-implantation epiblast^58^. Thus, our interactome analysis has uncovered a switch in OCT4 protein partners that orchestrates a dynamic reconfiguration of the enhancer landscape during the interconversion between naïve and primed pluripotent states (Fig. 8). This remodeling of the epigenetic landscape may explain the distinct developmental competencies of naïve and primed hESCs towards trophoblast and neural fates, respectively^17^.

The temporal expression dynamics of OCT4-associated factors between days 6 and 14 of human embryonic development recapitulate the expression changes observed during the naïve-to-primed transition in hESCs. While *BRM* and *KLF4* are activated in the preimplantation epiblast, *SOX2* is most significantly induced at later stages. Indeed, our ChIP-seq data suggest that SOX2 functions largely as a primed-specific TF in the human context, in contrast to its central role in naïve mouse ESCs^68^. Whether BRM also plays a previously underappreciated role in mouse ESCs and early embryogenesis will require future investigation. However, blastocyst outgrowths from double heterozygous intercrosses indicated a combined gene dosage requirement for *Brg1* and *Brm* during mouse peri-implantation development^69^. Such functional redundancy may provide a fail-safe mechanism to ensure adequate chromatin accessibility at the blastocyst stage before BRM expression is extinguished and BAF catalytic activity becomes exclusively reliant on BRG1 in the post-implantation epiblast. This transition is modelled during the naïve-to-primed transition in vitro, which we have shown here is critically dependent on BRG1 to promote adequate chromatin accessibility at primed-specific enhancers and promoters. We propose that BRM and BRG1 have overlapping functions in naïve hESCs, but not during later stages of development. This would explain why many human diseases are caused by haploinsufficiency of BAF subunits^41^. Consistent with this interpretation, *Brm* was required to prevent implantation defects but not neural tube defects in *Brg1* heterozygous mice^69^.

An important focus for future studies will be to identify functional enhancers that are activated by the BAF complex during the primed-to-naïve transition. Since BRG1 is essential for the self-renewal of primed cells, such experiments will likely require the use of inducible systems to deplete one or both catalytic subunits of the BAF complex at discrete stages of resetting. In addition, the OCT4 interactome data in this study provide a rich resource to explore other candidate regulators of human pluripotency. For example, it will be instructive to determine how ISWI complexes (containing SNF2H or SNF2L as ATPases) contribute to epigenetic remodeling in human pluripotent cells. Intriguingly, *Snf2h*^−/−^ mouse embryos die during the peri-implantation stage, which has been attributed to growth arrest in both ICM and trophectoderm lineages^70^. Furthermore, evidence from mouse ESCs indicates that SNF2H, but not BRG1, plays a dominant role in orchestrating chromosome folding and insulation of topologically associated domains^43^. Other candidates of interest include the chromatin remodeler BEND3^71^ and the RNA binding protein NLRP7^8^, both of which are naïve-specific OCT4 partners. Taking a cue from multiple studies in the mouse system over the past decade, we propose that elucidating the role of OCT4-associated proteins in human stem cells will likely yield important insights into the molecular basis of pluripotency, differentiation, and reprogramming.

## Methods

### Cell culture

#### Primed hESC culture

WIBR2 and WIBR3 primed hESCs were maintained on mitomycin C-inactivated mouse embryonic fibroblast (MEF) feeder layers in primed hESC medium (hESM) and passaged using Collagenase IV (Gibco, 1.5 mg/mL), as previously described^4,72,73^. Primed hESC medium consisted of DMEM/F12 supplemented with 15% fetal bovine serum, 5% KnockOut Serum Replacement, 1 mM GlutaMax (Gibco, 35050), 1% nonessential amino acids (Gibco, 11140), 1% penicillin-streptomycin (Gibco, 15140), 0.1 mM β-mercaptoethanol and 4–8 ng/ml FGF2. H9 primed hESCs were cultured in mTeSR Plus (STEMCELL Technologies, 05825) on Matrigel hESC-Qualified Matrix (Corning, 354277) coated wells and passaged using Gentle Cell Dissociation Reagent (STEMCELL Technologies, 07174) every 4 to 6 days.

#### Primed-to-naïve resetting

Chemically induced resetting of primed hESCs to naïve pluripotency was performed as previously described^4,15^. 2 × 10^5^ single primed cells were seeded on a six-well plate with MEF feeder layer in 2 mL primed hESC medium (for WIBR2/3) or mTeSR1 (for H9) supplemented with 10 μM Y-27632 (Stemgent, 04-0012). Two days later, medium was switched to 5i/L/A naïve media (see below for details). Ten days after seeding, the cells were expanded polyclonally using Accutase (Gibco, A1110501) or TrypLE Express (Gibco, 12604) on a MEF feeder layer. Media were changed every 1–2 days. For AP-MS experiments, we also performed transgene-mediated resetting to naïve pluripotency as previously described^4^. WIBR2-BirA and WIBR2-*OCT4*^*3x*FLB*io*^ human ESCs were infected with Doxycycline (Dox)-inducible KLF2 and NANOG lentiviral transgenes and maintained in 1 μM PD0325901 (Stemgent, 04-0006), 1 μM CHIR99021 (Stemgent, 04-0004), 20 ng/ml hLIF (Peprotech, 300-05), 2 µg/ml DOX, and 10 μM ROCK inhibitor Y-27632 (2i/L/DOX+RI).

#### Naïve hESC culture

Naive hESCs were cultured on mitomycin C-inactivated MEF feeder cells and were passaged by a brief PBS wash followed by single-cell dissociation using 5 min treatment with Accutase or TrypLE Express (Gibco, 12604) and centrifugation in fibroblast medium [DMEM (Millipore Sigma, #SLM-021-B) supplemented with 10% FBS (Millipore Sigma, ES-009-B), 1× GlutaMAX, and 1% penicillin-streptomycin]. Naïve hESCs were cultured in 5i/L/A media as previously described^4^. Five hundred milliliters of 5i/L/A was generated by combining: 240 mL DMEM/F12 (Gibco, 11320), 240 mL Neurobasal (Gibco, 21103), 5 mL N2 100× supplement (Gibco, 17502), 10 mL B27 50× supplement (Gibco, 17504), 1× GlutaMAX, 1× MEM NEAA (Gibco, 11140), 0.1 mM β-mercaptoethanol (Millipore Sigma, 8.05740), 1% penicillin-streptomycin, 50 µg/ml BSA Fraction V (Gibco, 15260), and the following small molecules and cytokines: 1 μM PD0325901 (Stemgent, 04-0006), 1 μM IM-12 (Enzo, BML-WN102), 0.5 μM SB590885 (Tocris, 2650), 1 μM WH4-023 (A Chemtek, H620061), 10 μM Y-27632 (Stemgent, 04-0012), 20 ng/mL hLIF (Peprotech, 300-05), and 10 ng/mL Activin A (Peprotech, 120-14). For MS experiments, the GSK3 inhibitor IM-12 was omitted from the 5i/L/A cocktail to achieve enhanced proliferation (4i/L/A), as previously described^15^. *BRM*-targeted and *BRG1*-targeted naïve hESCs were transferred from 5i/L/A to PXGL^74^ since this maintenance medium better supported clonal expansion following CRISPR/Cas9-mediated genome editing. PXGL media consisted of N2B27 supplemented with 1 μM PD0325901, 2 μM XAV939 (Selleckchem, S1180), 2 μM Gö6983 (Tocris, 2285), and 20 ng/mL human LIF. 10 μM Y-27632 was added during passaging. All naïve hESC experiments were conducted in 5% O_2_, 5% CO_2_.

#### SILAC labeling

To perform quantitative mass spectrometry based whole-proteome comparison of primed and naive pluripotent states, WIBR2 and WIBR3 primed hESCs were cultured in SILAC heavy medium whereas chemically reset naïve hESCs were cultured in 4i/L/A naïve medium (SILAC light). For the SILAC heavy condition, primed hESCs were grown for three passages in DMEM/F12 with corresponding complete supplements but deficient in both L-lysine and L-arginine and supplemented with heavy ^13^C_6_^15^N_4_ L-arginine and ^13^C_6_^15^N_2_ L-lysine (Cambridge Isotope Laboratories). The medium was supplemented with 10% dialyzed FBS for SILAC (Thermo Fisher Scientific), and all other gradients followed the normal condition for primed hESC culture.

#### Repriming of naïve hESCs

The naïve-to-primed pluripotency transition (repriming) was performed as previously described^75^. Naïve hESCs were single cell dissociated using TrypLE Express and 5 × 10^5^ cells were seeded per Matrigel-coated well in mTeSR Plus supplemented with 10 µM Y-27632. The cells were cultured in 5% CO_2_ and 20% O_2_. After 2 days, Y-27632 was withdrawn and cells were collected on d5 for RNA-seq analysis. To improve survival and sample quality for ATAC-seq analysis, the repriming protocol was modified by seeding naïve hESCs on a MEF feeder layer and cells were harvested 4 days after transfer to mTeSR Plus media.

### Donor vectors for gene targeting in hESCs

*AAVS1* and *OCT4* TALEN plasmids were designed and assembled as previously described^72^. Intron 1 of the *AAVS1* locus was targeted to constitutively express biotin ligase BirA. The *OCT4* loci were targeted to express the 3xFLAG sequence followed by a 23 amino acid recognition for biotin ligase BirA, which becomes biotinylated on the lysine residue. Donor vectors were constructed by PCR amplifying homology arms from the corresponding loci using genomic DNA isolated from hESCs. The homology arms followed by either biotin ligase of *E. coli* BirA or 3xFLAG-Biotin sequences were cloned into pCR2.1-TOPO vector (Invitrogen) using standard cloning methods.

### TALEN-mediated gene targeting in hESCs

To target the CAGGS-BirA-V5His sequence to the *AAVS1* locus, WIBR2 human ESCs were cultured with ROCK inhibitor (10 µM) 24 h prior to electroporation. Cells were harvested using 0.25% trypsin/EDTA solution (Invitrogen) and resuspended in phosphate buffered saline (PBS). Ten million cells were electroporated with 40 μg of donor vector and 5 μg of each *AAVS1* TALEN encoding plasmid (Gene Pulser Xcell System, Bio-Rad: 250 V, 500 μF, 0.4 cm cuvettes). Cells were subsequently plated on DR4 MEFs feeder layer with neomycin for selection in hESCs medium supplemented with 10 μM ROCK inhibitor (Y-27632) for the first 24 h. Individual colonies were picked and expanded after neomycin selection (300 μg/ml) 10–14 days after electroporation. Gene targeting analysis was verified by Southern blotting (EcoRV digested). To introduce a 3xFLAG-Biotin sequence at the C-terminus of endogenous *OCT4*, WIBR2-BirA hESCs were used and the same electroporation protocol was applied with 40 μg of donor vector and 5 μg of each OCT4 TALEN encoding plasmid. Correctly targeted clones were confirmed by Southern blot analysis (BamHI digested). Then the PGK-Puro selection cassette was removed by transient expression of Cre recombinase in WIBR2-*OCT4*^*3x*FLB*io*-*Puro*^ hESCs. Briefly, hESCs were transfected with 40 μg pTurbo-Cre (GenBank accession number AF334827) and 10 μg fluorescent FUW-Tomato vector with the same electroporation protocol. Two days later, Tomato-expressing cells were FACS-purified and replated at low density on MEF feeder layers. Individual colonies were picked and expanded 10–14 days after FACS sorting. The excision of the PGK-puro selection cassette was confirmed by Southern blot analysis.

### Southern blot

Genomic DNA was extracted from WIBR2, WIBR2-BirA, and WIBR2-*OCT4*^*3x*FLB*io*^ hESCs (before and after Cre excision of the PGK-Puro selection cassette) by tail lysis buffer (10 mM Tris-Cl (pH8.0), 100 mM NaCl, 10 mM EDTA, 0.5% SDS and 0.4 mg/ml proteinase K) and incubated overnight at 37 °C. The next morning an equal volume of isopropyl alcohol was added and centrifuged at 6000 × *g* for 5 min. The supernatant was discarded, and the pellet resuspended in 1 ml of 70% ethanol for washing and centrifuged at 6000 × *g* for 5 min. At this point the supernatant was carefully decanted and the pellet air dried before being resuspended in 200 μl TE buffer. Five micrograms aliquots were digested overnight with the appropriate enzymes (EcoRV for *AAVS1* targeting, BamHI for *OCT4* targeting) at 37 °C. Digested genomic DNA was separated on a 0.8% agarose gel followed by capillary transfer to a Hybond N^+^ membrane (Amersham Biosciences). For making an *AAVS1*-specific internal probe, the donor vector was digested with SacI and EcoRI enzyms to generate a 643 bp fragment of 5′ arm of *AAVS1*. External probes specific to *OCT4* were amplified from genomic DNA outside the region of the targeting arms. These DNA fragments were labeled by using [∝-^32^P] dCTP and Prime-It II Random Primer Labeling Kit (Agilent Technologies) according to the manufacturer’s protocol. Hybridization was carried out overnight at 65 °C. Post-hybridization washes were done sequentially with two standard saline citrate (SSC) solutions (20× SSC; 3 M NaCl in 0.3 M sodium citrate (pH 7.0)), 2× SSC, and 0.2× SSC along with 0.2% SDS, each for 20 min at 65 °C. Blots were exposed at −80 °C with X-ray film in film cassette for 24 h. Finally, the films were developed by an auto-processor in a darkroom.

### Immunostaining

Cells in 12-well plates were fixed in PBS supplemented with 4% paraformaldehyde for 20 min at room temperature. The cells were then permeabilized and blocked using 0.2% Triton X-100, 0.1% Tween-20 and 3% donkey serum in PBS for 30 min. Primary antibody against human OCT4 (1:500, Santa Cruz, sc-5279), human SOX2 (1:500, R&D Systems, AF2018), and human NANOG (1:250, goat polyclonal, R&D Systems, AF1997) was diluted in 0.2% Triton X-100 in PBS and incubated with the samples overnight at 4 °C. The cells were treated with an appropriate Molecular Probes Alexa Fluor® dye conjugated secondary antibodies (Donkey-α-Mouse Alexa Fluor® Plus 594 Cat# A32744 and Donkey-α-Goat Alexa Fluor® Plus 488 Cat# A32814, both 1:500, Invitrogen) and then incubated for 1 h. The nuclei were stained with DAPI for 10 min.

### Teratoma formation

WIBR2-BirA and WIBR2-*OCT4*^*3x*FLB*io*^ hESCs were collected by Collagenase treatment and separated from feeder cells by subsequent washes with medium and sedimentation by gravity. The cells were resuspended in 250 µl of PBS and injected subcutaneously into SCID mice. Tumors developed within 4–8 weeks and animals were sacrificed before tumor size exceeded 1.5 cm in diameter. Teratomas were isolated after sacrificing the mice and fixed in formalin. After sectioning, teratomas were diagnosed based on hematoxylin and eosin staining.

### Affinity purification of OCT4-associated protein complexes

To identify OCT4-associated proteins in primed and naive hESCs, Streptavidin (SA) and FLAG pulldown were performed in WIBR2-*OCT4*^*3x*FLB*io*^ and WIBR2-BirA (control) ESCs, and two replicates of endogenous OCT4 antibody pulldown were performed in wild-type WIBR2 and WIBR3 hESCs (compared to IgG control). All cell lines for AP-MS were expanded to about fifteen confluent 15 cm diameter dishes before harvest. For primed hESCs, the colonies were collected by Collagenase IV (Gibco, 1 mg/ml) treatment and separated from feeder cells by washes with medium and sedimentation by gravity. For naïve hESCs, SA and 3xFLAG pulldown were performed in naive WIBR2-*OCT4*^*3x*FLB*io*^ and WIBR2-BirA hESCs infected with DOX-inducible KLF2 and NANOG transgenes and cultured in 2i/L/DOX+RI, and endogenous OCT4 antibody pulldown was performed in hESCs cultured in 4i/L/A medium (omission of GSK3β inhibitor IM-12) cocktail to achieve enhanced proliferation, as previously described^15^. Naïve hESCs were collected by dissociation using 3–5 min treatment with Accutase and centrifugation in fibroblast medium.

Nuclear extraction and affinity purification of 3xFLBio-tagged OCT4-associated complexes were performed as previously described^76^. Briefly, the cell pellets were resuspended in ice-cold hypotonic buffer A (10 mM HEPES, pH 7.9, 1.5 mM MgCl_2_, 10 mM KCl, 0.5 mM DTT, 0.2 mM PMSF and protease inhibitor cocktail (Roche) and incubated for 10 min on ice. The sample was centrifuged at 4500 × *g* for 5 min at 4 °C and the pellet containing nuclei was washed by resuspending with 3 ml of ice-cold buffer A and centrifuging at 25,000 × *g* for 20 min at 4 °C. Then, nuclei were resuspended with 3 ml of ice-cold nuclear extract buffer C (20 mM HEPES, pH 7.9, 20% glycerol (v/v), 0.42 M NaCl, 1.5 mM MgCl_2_, 0.2 mM EDTA, 0.5 mM DTT, 0.2 mM PMSF, and protease inhibitor cocktail) and incubated at 4 °C for 30 min. Insoluble materials were pelleted by centrifugation at 25,000 × *g* for 20 min at 4 °C. The supernatant was collected as nuclear extract (NE) and dialyzed against buffer D (20 mM HEPES, pH 7.9, 20% glycerol (v/v), 100 mM KCl, 0.2 mM EDTA, 0.5 mM DTT, 0.2 mM PMSF) at 4 °C for 3 h. Then, 0.1 ml of Protein G agarose (Roche Diagnostic) equilibrated in buffer D containing 0.02% NP40 (buffer D-NP) was added to nuclear extracts in 15 ml tubes (BD Falcon), in the presence Benzonase (25 U/mL, Millipore 70664), and incubated/precleared for 1 h at 4 °C with continuous mixing. For SA-IP, nuclear extracts were incubated with streptavidin-agarose beads (200 uL beads per IP, Invitrogen, 15942-050) and rotated for 6 h at 4 °C. For FLAG-IP, precleared extracts were incubated with pre-equilibrated ANTI-FLAG M2 affinity gel (200 uL slurry per IP, Sigma, F2426) for 3 h at 4 °C. For OCT4 antibody IP, precleared extracts were incubated with 20 ug OCT4 primary antibody (Santa Cruz, sc-5279) or mouse IgG (Millipore 12–371) overnight with rotation at 4 °C, then incubated with Protein G agarose beads for another 2 h. For all samples after beads incubation, five washes were performed with buffer D-NP. Bound material was eluted by boiling for 5 min in Laemmli buffer and fractionated on a 10% SDS-PAGE. The gel lanes were horizontally cut into 8–10 pieces and each piece was subjected to digestion with porcine trypsin (Promega) as previously described^76^. The resulting peptides from each piece were dried down and analyzed by LC-MS/MS.

### Mass spectrometry and proteomics data analysis

The samples were reconstituted in 5–10 μl of HPLC solvent A (2.5% acetonitrile, 0.1% formic acid). A nano-scale reverse-phase HPLC capillary column was created by packing 5 μm C18 spherical silica beads into a fused silica capillary (100 μm inner diameter × 12 cm length) with a flame-draw tip. After equilibrating the column each sample was loaded onto the column. A gradient of acetonitrile from 2.5 to 97.5% was used to elute the peptides. As peptides eluted, they were subjected to electrospray ionization and then they entered into an LTQ-Orbitrap-Velos mass spectrometer (Thermo Finnigan) with collision-induced dissociation (CID). Eluting peptide were detected, isolated, and fragmented to produce a tandem mass spectrum of specific fragment ions for each peptide. MS data were processed by Thermo Proteome Discoverer software with SEQUEST engine against Swiss-Prot human protein sequence database (available at https://www.uniprot.org/).

Outputs of protein identification from Proteome Discoverer were imported into a local Microsoft Access database. Duplicated records were removed by unique protein symbol. Common contamination proteins (trypsin, keratins, Actin, Tubulins) were removed, and protein lists were filtered by identification score >10, and number of identified peptides >2. A protein needed to be identified in three out of four experiments to be considered an interactor candidate. Proteins present in more than 20% of CRAPome^37^ (available at http://www.crapome.org/) experiments were removed prior to calculation of empirical p-values. OCT4 interactors were chosen from the list of proteins with a combined cumulative probability (CCP) score as described previously^38^, with the following minor modifications to the algorithm. We multiplied the ratio of spectral counts between pull-down and control experiments by the number of spectral counts in pull-down experiments prior to calculation of the empirical *p*-value. This rewards proteins with high spectral counts in pull-down experiments, which is necessary when working with a more sensitive MS platform. A false-discovery-rate (FDR) of 0.1 was applied as the significance cutoff of OCT4 interactors.

### Co-immunoprecipitation (co-IP) and western blot

Co-IP in regular condition was performed with the same protocol of OCT4 antibody IP-MS experiments but with a lower number of cells. Usually, confluent 15 cm diameter dishes of WIBR2 and WIBR3 cells in the primed and naïve (4i/L/A or 5i/L/A) conditions were harvested for co-IP. In high-salt co-IP, the cell nuclei were harvest with the sample protocol of IP-MS experiment. Then proteins were extracted with buffer C (containing 420 mM NaCl), incubated with the antibody overnight, and with Protein G agarose beads for another 2 h. Beads were washed four times with buffer C. The following antibodies were used: OCT4 (2 ug per IP, Santa Cruz, sc-5279), BRG1 (2 ug per IP, Santa Cruz, sc-17796), BRM (2 ug per IP, Bethyl, A301-015A), and the same amount of mouse IgG (Millipore, 12–371) or rabbit IgG (Millipore, PP64) as a control. The IPed samples were boiled with Laemmli/SDS Buffer.

For western lot analysis of protein expression in primed and naïve (5i/L/A) hESCs, cells from a confluent six-well were harvested and resuspended in radioimmunoprecipitation assay (RIPA) buffer, then incubated on ice for 20 min. Whole-cell extract concentration was measured by Bradford assay (Pierce, 23236). Proteins were balanced and subject to SDS-PAGE analysis. Primary antibodies used were FLAG (Sigma, F1804), V5 (Invitrogen, #1030648), Streptavidin-HRP Conjugate (diluted in TBS/T buffer, GE Healthcare, RPN1231), GAPDH (1:5000, Proteintech, 10494-1-AP), ACTIN (1:5000, Sigma, A5441), L1TD1 (Sigma, HPA030064), SMC1A (Abcam, ab137707), SMC3 (Santa Cruz, sc-8198), MSH2 (Santa Cruz, sc-494), MSH6 (BD Biosciences, 610918), BAF155 (Santa Cruz, sc-32763), OCT4 (Santa Cruz, sc-5279). SNF2L (Cell Signaling Tech., #12483), BRM (Bethyl, A301-015A), BRG1 (Santa Cruz, sc-17796), SNF2H (Santa Cruz, sc-13054), BAF47 (Bethyl, A301-087A), BAF53A (Santa Cruz, sc-137062), BAF60B (Santa Cruz, sc-101162), BAF60A (Santa Cruz, sc-514400), BAF170 (Santa Cruz, sc-17838), SOX2 (R&D systems, AF2018), KLF4 (R&D Systems, AF3640), NANOG (Abcam, ab109250), TFAP2C (Santa Curz, sc-12762). If not specified, primary antibodies were diluted by 1:1000 in TBS/T buffer with 5% bovine serum albumin. BEND3 primary antibody is kindly provided by Dr. Supriya Prasanth from University of Illinois at Urbana–Champaign.

### Flow cytometry analysis

Cells were single cell dissociated using TrypLE Express and washed once in FACS buffer [PBS supplemented with 5% FBS]. The cells were then resuspended in 100 μL fresh FACS buffer, and incubated with antibodies for 30 min on ice. The following naïve-specific cell-surface antibodies were used: anti-SUSD2-PE, 1:100 (BioLegend, 327406) and anti-CD75-eFluor 660, 1:100 (Thermo Fisher, 50-0759-42). Following antibody incubation, the cells were washed once with FACS buffer, resuspended in fresh FACS buffer, and passed through a cell strainer. Cell debris was excluded by FSC vs. SSC gates and single cells were gated by FSC-A vs. FSC-W. Naïve cell-surface markers were analyzed with anti-CD75-eFluor 660 (APC channel) and anti-SUSD2-PE. Primed cells and unstained cells that have undergone the same procedures were used as controls. Flow cytometry analysis was performed using a BD LSRFortessa X-20 and the data were analyzed using the FlowJo software.

### BRM and BRG1 CRISPR targeting

Guide RNAs (gRNAs) aimed at introducing out-of-frame indels to trigger nonsense-mediated decay of the transcripts of the human *BRM (SMARCA2)* and *BRG1 (SMARCA4)* genes were designed and validated in K562 cells by the Genome Engineering and iPSC center (GEIC) at Washington University. In short, synthetic gRNAs (Supplementary Data 6) were complexed with recombinant Cas9 protein and nucleofected into K562 cells. Transfected cells were harvested and lysed 48–72 h post-nucleofection. Each target region was PCR amplified, indexed, and analyzed on a MiSeq for indel rate, indicative of cleavage activity. The most efficient gRNAs for each target (sp4 and sp13)  were selected for nucleofection into hESCs. 1.5 × 10^6^ primed H9 hESCs were transfected with 0.5 µg pmaxGFP control vector, 300 pmol gRNA, and 192 pmol Cas9 protein. gRNA complexes targeting *BRM* or *BRG1* were transfected into primed hESC by nucleofection using the Amaxa P3 Primary Cell 4D-Nucleofector X Kit and 4D-Nucleofector device with program CA-137 (Lonza). Forty eight hours after nucleofection, the cells were single cell dissociated and sorted for GFP-expressing (~15%) cells using a Sony H800 flow cytometry system. Clonal lines were analyzed by NGS for presence of insertion–deletions (indels) around the cut site. Samples that exhibited a mixed indel ratio were discarded. Primed *BRM*^*+/+*^*, BRM*^+/−^, or *BRM*^−/−^ hESCs were converted to naïve pluripotency in 5i/L/A and further expanded in PXGL medium, which supports enhanced clonal expansion. 1 × 10^6^ wild-type (WT) and *BRM*^−/−^ naïve hESCs were then transfected with 1 µg pmaxGFP, 300 pmol gRNA targeting *BRG1*, and 192 pmol Cas9 protein with GeneJuice following manufacturer’s instructions (Millipore, #70967). GFP-expressing cells were sorted after 48 h, and pools were harvested for NGS two passages later to assess indel frequencies. To generate clonal lines of naïve *BRG1*^−/−^ hESCs, WT and *BRM*^+/−^ naïve hESCs were transfected with 1 ug pmaxGFP, 300 pmol gRNA targeting *BRG1*, and 192 pmol Cas9 protein with GeneJuice, and GFP-expressing cells were FACS sorted 48 h post-transfection. Individual sorted cells were replated on inactivated MEFs in 96-well plates in PXGL and naïve media were replaced every 2 days. Individual colonies were picked and expanded in 24-well plates. Mutations were validated by purified genomic DNA in the GEIC at Washington University and the absence of BRG1 protein was validated by Western blotting.

### Knockdown by lentiviral shRNAs

VSV-glycoprotein pseudotyped lentiviral vector particles were produced by co-transfection of HEK293T cells with packaging and envelope plasmids as previously described^77^. The pLKO.1-puro (SIGMA) plasmids targeting BRM (TRCN0000358828, TRCN0000020329, TRCN0000367881), BRG1 (TRCN0000015549, TRCN0000015550) and two Non-Mammalian shRNA Control Plasmid (SIGMA, #SHC002 and #SHC016) were used. Viral supernatants were harvested after 48 h and filtered through a 0.45 μm membrane. WIBR3 hESCs maintained in primed hESC medium and WIBR3 naïve hESCs maintained in 5i/L/A were infected with the lentivirus in the presence of 6 μg/ml polybrene (Millipore). Infected cells were plated on mitomycin C-inactivated DR4 feeder cells and selected with 0.5 μg/ml of puromycin starting 2 days after transduction. Total RNA was extracted after 5 days of puromycin selection.

### Quantitative real-time PCR

To assess the expression of *BirA* transgene following AAVS1 targeting in primed hESCs, total RNA was isolated using the Rneasy Kit (QIAGEN) and reversed transcribed using the Superscript III First Strand Synthesis kit (Invitrogen). Quantitative RT-PCR analysis was performed in triplicate using the ABI 7900 HT system with FAST SYBR Green Master Mix (Applied Biosystems). Gene expression was normalized to GAPDH. Error bars represent the standard deviation (SD) of the mean of triplicate reactions. Primer sequences are included in Supplementary Data 6.

### RNA-seq analysis

RNA-seq analysis was performed in naïve and primed hESCs in which the BAF ATPases were genetically perturbed by either shRNA-mediated knockdown of WIBR3 cells and CRISPR/Cas9-mediated knockout of H9 cells. Total RNA was extracted using E.Z.N.A. Total RNA kit (Omega Bio-Tek, R6834) with DNase I following the manufacturer’s instructions. Samples were prepared according to library kit manufacturer’s protocol, indexed, pooled, and sequenced on an Illumina HiSeq at the Genome Technology Access Center of Washington University in St. Louis. Single-end reads with 50 bp length were generated from the knockdown experiment of WIBR3 cells and paired-end reads with 150 bp length were generated from the knockout experiment of H9 cells.

### RNA-seq data processing

RNA-seq data from public resources and our study (see Supplementary Data 6) were processed together. Briefly, single-end reads were aligned to the human genome using TopHat (v2.0.10) and Bowtie2 (v2.1.0) with the default parameter settings. Paired-end reads were aligned to the human genome using STAR (v2.5.3) with the default parameter settings. The UCSC hg38 human genome, as well as the transcript annotation, was downloaded from the iGenomes site. The aligned bam files were sorted. Transcript assembly and differential expression analyses were performed using Cufflinks (v2.1.1). Assembly of novel transcripts was not allowed (-G), other parameters of Cufflinks followed the default setting. The summed RPKM (reads per kilobase per million mapped reads, for single-end RNA-seq) or FPKM (fragments per kilobase per million mapped reads, for paired-end RNA-seq) of transcripts sharing each gene_id were calculated and exported by the Cuffdiff program. In the gene expression matrix, a value of RPKM+0.1 was applied to the samples of single-end data and a value of FPKM+1 was applied to the samples of paired-end data, to minimize the effect of low-expression genes. *P* values were calculated using a *t*-test. Differentially expressed genes (DEGs) were by two-sided *t*-test *P* value < 0.05 and fold change > 2. Boxplots for expression were generated using R. *P* value was calculated from two-sided Mann–Whitney test.

PCA analysis was performed for RNA-seq data from different batches. Batch effects were adjusted by *ComBat* function implemented in the *sva* Bioconductor package (v.3.18.0). The expression data matrix was imported by Cluster 3.0 software (http://bonsai.hgc.jp/~mdehoon/software/cluster/software.htm) for PCA analysis. PC values were visualized with the *plot3d* function in the *rgl* package from CRAN. All R scripts were processed on R-Studio platform (v3.6.1).

### Transcriptome analyses of human and non-human primate embryos

Figures 4f and 5g: Processed single-cell RNA-seq data from 3D-cultured human pre-gastrulation embryos^57^ (GSE136447) were downloaded. Genes with very low expression (average(log_2_(FPKM + 1)) < 1) potentially due to low capture rates over all cell types and timepoints were removed. Plots representing the average expression of selected markers by color and the percentage of epiblast cells that express the marker by point size was generated by using the ‘*DotPlot’* function of the R *Seurat* package. Expression of C1 and C6 target genes in human embryos were obtained based on annotation of the BAF peaks (refer to ChIP-seq analysis, Supplementary Data 3). Boxplots were generated using R.

Supplementary Figure 5c: To examine the expression of C1 and C6 target genes (Supplementary Data 3) in non-human primate embryos, single-cell pre- and post-implantation cynomolgus monkey gene expression data^59^ (GSE74767) were acquired and filtered for mapped unique gene IDs and valid numeric expression values. Using a one-to-one gene ID lookup table (Nakamura et al. 2016, Supplementary Data 2), orthologs of expression target genes (from C1 and C6) were identified. Each cell’s average expression for all genes in a cluster was plotted for each developmental stage using R.

### ChIP-seq analysis

ChIP-seq was performed on WIBR3 cells cultured in primed and naïve (5i/L/A) conditions. Cells were single cell dissociated, resuspended in their respective media at a concentration of 1 million cells/mL, crosslinked in 1% formaldehyde at 37 °C for 10 min, quenched in glycine (0.125 M) for 5 min, and resuspended in ice-cold PBS. ChIP was performed following an EZChIP protocol from Millipore (#17-371). Briefly, about 5 million hESCs were used for each ChIP experiment. Sonication was performed on a Bioruptor system, with 30 s ON, 30 s OFF, 30 cycles, high amplitude. The primary antibodies used for ChIP were: BRG1 (5 ug, Abcam, ab110641), BRM (3 ug, Cell Signaling Tech., #11966), BAF155 (3 ug, homemade in Dr. Kadoch’s lab), and SOX2 (5 ug, R&D Systems, AF2018). 10% of sonicated genomic DNA was used as ChIP input.

For BRG1, BRM, and BAF155, library prep and sequencing were performed on Illumina NextSeq 500 in the Molecular Biology Core Facilities at the Dana-Farber Cancer Institute. Data with 75 bp single-end reads were obtained. For SOX2, ChIP libraries were prepared using the NEBNext Ultra II DNA library prep kit and index primers sets (NEB, #7645 S, #E7335S) followed the standard protocol. Massively parallel sequencing was performed by Novogene Co. with the Illumina HiSeq 4000 Sequencer according to the manufacturer’s protocol. Libraries were sequenced as 150 bp paired-end reads.

### CUT&Tag analysis

Cleavage Under Targets and Tagmentation (CUT&Tag) analysis to interrogate KLF4 binding in WIBR3 primed and naïve (5i/L/A) hESCs was performed as described previously^78^ with minor modifications. Briefly, 200,000 cells per sample replicate were washed in Wash Buffer [1 mL 1 M HEPES pH 7.5 (Sigma–Aldrich, H3375), 1.5 mL 5 M NaCl (Sigma–Aldrich, S5150), 12.5 μL 2 M Spermidine (Sigma–Aldrich, S2501), 1 Roche Complete Protease Inhibitor EDTA-Free tablet (Sigma–Aldrich, 5056489001), and bring the final volume to 50 mL with dH2O], then immobilized on 10 ul of Concanavalin A-coated beads (Bangs Laboratories). Cells were cleared on a magnetic rack, then permeabilized with Dig-wash buffer [20 mM HEPES pH 7.5, 150 mM NaCl, 0.5 mM Spermidine and 1× protease inhibitor cocktail containing 0.05% Digitonin]. The cells were then incubated with primary KLF4 antibody (R&D, AF3640, 1:100) at 4 °C overnight. The primary antibody was cleared on a magnetic rack. Rabbit anti-Goat IgG secondary antibody was diluted 1:100 in Dig-wash buffer and incubated at RT for an hour. Cells were cleared on a magnetic rack and washed with 1 mL of Dig-wash buffer. A 1:200 diluted of pA-Tn5 adapter complex was prepared in Dig-300 Buffer [20 mM HEPES pH 7.5, 300 mM NaCl, 0.5 mM Spermidine and 1× protease inhibitor cocktail containing 0.05% Digitonin]. Cells were cleared on a magnetic rack and incubated by adding 100 ul of pA-Tn5 at RT for 1 h. Cells were washed with 1 mL of Dig-300 buffer, resuspended in 300 ul of Tagmentation buffer [10 mM MgCl_2_ in Dig-300 Buffer], and incubated at 37 °C for 1 h. 10 ul of 0.5 M EDTA, 3 ul of 10% SDS, and 2.5 ul of 20 mg/mL Proteinase K was added to each reaction to stop the tagmentation at 55 °C for an hour. DNA was purified using phenol/chloroform/isoamyl alcohol (PCI) extraction followed by chloroform extraction and precipitated with glycogen and ethanol. DNA was pelleted with a high-speed spin at 4 °C, washed, air dried for 5 min and resuspended in 50 ul of double-distilled water (ddH_2_O). The DNA was then PCR amplified using i5 and i7 indexing primers, and cleaned up with AMPure XP beads, and the size distribution and concentration were confirmed using Tapestation. The libraries were then sequenced on an Illumina NovaSeq S4 2 × 150 platform.

### ATAC-seq analysis

ATAC-seq was performed as previously described^17^ on H9 *WT*, *BRG1*^+/−^ and *BRG1*^−/−^ naïve hESCs, *WT*, *BRG1*^+/−^, and *BRG1*^−/−^ cells at day 4 of repriming, and *WT* primed hESCs. Briefly, cells were harvested by TrypLE Express dissociation and centrifuged at 500 RCF for 5 min at 4 °C. After aspirating the supernatant, cells were washed once with cold PBS containing 0.04% BSA. Cell pellets were resuspended in 300 μl DNaseI (ThermoFisher, EN0521) solution [20 mM Tris pH 7.4, 150 mM NaCl, 1× reaction buffer with MgCl_2_, 0.1U/ul DNaseI] on ice for 5 min. After DNase treatment, 1 ml PBS containing 0.04%BSA was added and cells were centrifuged at 500 rcf for 5 min at 4 °C. Another two washes were done in 1 ml PBS containing 0.04% BSA. Cell pellets were resuspended in 100 ul ATAC-seq RSB [10 mM Tris pH 7.4, 10 mM NaCl, 3 mM MgCl_2_ in water] consisting of 0.1% NP40, 0.1% Tween-20, and 0.01% digitonin by pipetting up and down and incubating on ice for 3 min. After lysis, 1 mL of ATAC-seq RSB containing 0.1% Tween-20 was added and inverted with the lysis reaction. Then, nuclei were pelleted by centrifugation at 800 RCF for 5 min at 4 °C. Supernatant was removed, and the nuclei were resuspended in 20 µL 2× TD buffer [20 mM Tris pH 7.6, 10 mM MgCl_2_, 20% Dimethyl Formamide]. 50,000 counted nuclei were then transferred to a tube with 2× TD buffer filled up to 25 µL. 25 µL of transposition mix [2.5 µL Transposase (100 nM final) (Illunina, 20034197, 16.5 µL PBS, 0.5 µL 1% digitonin, 0.5 µL 10% Tween-20, and 5 µL water) was added. Transposition reactions were mixed and incubated for 30 min at 37 °C with gentle tapping every 10 min. Reactions were cleaned up with the Zymo DNA Clean and Concentrator-5 kit (Zymo Research, D4014). The ATAC-seq library was amplified for nine cycles on a PCR machine. The PCR reaction was purified with Ampure XP beads (Beckman Coulter, A63880) using double size selection following the manufacturer’s protocol, in which 27.5 µL beads (0.55× sample volume) and 50 µL beads (1.55× sample volume) were used based on 50 µL PCR reaction. The ATAC-seq libraries were quantitated by Qubit assays and sequenced by an Illumina NextSeq platform. QC and analysis on ATAC-seq libraries was performed using AIAP^79^. The generated peaks files for each library were incorporated with bedtools merge and counts on each peak were quantified for all libraries using bedtools coverage.

### ChIP-seq, CUT&TAG, and ATAC-seq data processing

ChIP-seq and ATAC-seq data from public resources and our study (see Supplementary Data 6) were processed together with the same settings. Briefly, reads were preprocessed by trim_galore (v0.6.3) and aligned to the hg38 human genome using the bowtie2 (v2.3.4) program. For single-end reads, the bowtie2 program followed the default setting. The aligned reads were exported (-F 0 × 04), sorted, duplicates-removed with samtools (v0.1.19). For paired-end reads, bowtie2 parameters were “-X 1000–no-mixed --no-discordant”. The aligned paired reads were exported (-F 0 × 04 -f 0 × 02) and sorted with samtools. Duplicates were removed with MarkDuplicates function in the PICARD (v2.14.0) package. All Bam files were converted to a binary tiled file (tdf) and visualized using IGV (v2.7.2) software.

All ChIP-seq peaks were determined by the MACS2 program (v.2.0.10) using the input ChIP-seq as the control data, and all other parameters followed the default settings. Peaks of TFs (SOX2, KLF4, OCT4) were called as narrow peaks, and peaks of histone marks and BAF components were called as broad peaks. ATAC-seq peaks in naïve and primed hESCs^48^ were determined by MACS2 program with the default settings. The R package *diffbind* (v1.16.3) from bioconductor was used to determine the common ATAC peaks, with a minimal overlap from 3 (out of 4) replicates. All peaks were annotated using the annotatePeaks module in HOMER program (v4.11) against the hg38 genome. A target gene of a called peak was defined as nearest gene’s transcription start site (TSS) with a distance to TSS less than 20 kb.

The BAF peaks were merged from the highly concordant BRG1 and BAF155 peak regions with the merge function in bedtools (v2.18.1) package in either naïve or primed hESCs. The common peaks were determined by the intersect function in bedtools package. Overlapped peaks were defined as a mutual overlap with a minimal 25% regions covered by each other (-f 0.25 -F 0.25 –e). For all the BAF peaks determined in naïve and primed hESCs, the presence or absence of overlapping peaks with OCT4 and the H3 histone marks was determined using the criteria above for the intersect function in bedtools to create a binary table of overlaps for each BAF peaks. The interactions among the BAF binding sites, OCT4 binding sites, and histone modifications were categorized by k-means clustering using R with the number of clusters set at k = 10 and parameters iter.max = 1000 and nstart = 1000. Clusters were annotated based on the pattern of histone marks at each cluster. Motif analysis was performed for each cluster with the findMotifsGenome module (-size given) in the HOMER program (v4.11). Heatmaps and mean intensity curves of ChIP-seq data at specific genomic regions were plotted by the NGSplot tools (v2.61, available at https://github.com/shenlab-sinai/ngsplot) centered by the middle point “(start+end)/2” of each region. The significance (*P* value) assessing the overlap between two groups of genomic regions was calculated by Fisher-exact test with an alternative hypothesis of greater on R platform.

For analysis of ATAC-seq data of naïve hESCs and repriming samples, the ATAC-seq read intensity at BAF peaks was calculated by the *diffbind* package of R (v1.16.3). The significantly increased or decreased ATAC peaks were obtained by cutoffs FDR<0.05. The M-A intensity plots were created by the *plotMA* function in the *diffbind* package. The predicted number of ATAC peaks in each BAF cluster was calculated by the total number of significantly regulated peaks multiplied by the portion of each BAF cluster among total BAF peaks. A *P* value was calculated by the fisher-extract test using the R platform.

### GO and GSEA analysis

The gene ontology (GO) analysis for the genomic locations of BAF peaks was performed with the GREAT tool (v3.0, http://great.stanford.edu/). GO analysis for the significantly regulated genes was performed with the DAVID tool (v6.8, https://david.ncifcrf.gov/tools.jsp). *P* value is from the right-sided Fisher’s Extract test. Geneset enrichment analysis (GSEA, v3.0, available at https://www.broadinstitute.org/gsea) was used to determine the enrichment of ICM- or hESC-signatures in naïve vs. primed hESCs. The gene signatures were obtained from a published RNA-seq dataset that reports stage-specific gene expression in early human embryos^53^. The normalized enrichment score (NES) and FDR q value were indicated for each enrichment test.

## Supplementary information

Supplementary Information

Description of Additional Supplementary Files

Supplementary Data 1

Supplementary Data 2

Supplementary Data 3

Supplementary Data 4

Supplementary Data 5

Supplementary Data 6

## Data Availability

The data that support this study are available from the corresponding authors upon reasonable request. The OCT4 affinity purification mass spectrometry data have been deposited to the ProteomeXchange Consortium via the PRIDE partner repository with the dataset identifier PXD026556. The ATAC-seq, ChIP-seq, CUT&Tag, and RNA-seq data generated in this study have been deposited in the Gene Expression Omnibus (GEO) database under accession codes GSE147751 and GSE168002. The ATAC-seq and TFAP2C ChIP-seq data (GSE101074), OCT4 and H3K27ac ChIP-seq data (GSE69647), and H3K4me3 and H3K27me3 ChIP-seq data (GSE59435) in naïve and primed hESCs were analyzed in this study. The ATAC-seq and RNA-seq data of human embryos (GSE101571), and ATAC-seq and ChIP-seq data (GSE122631) of human NPCs, and SOX2 ChIP-seq data (GSE69479) in human NPCs were analyzed in this study. Source data are provided with this paper.

## Source data

Source Data ZIP file
