# Supplementary material for: OCT4 cooperates with distinct ATP-dependent chromatin remodelers in naïve and primed pluripotent states in human
Source: Nat Commun. 2021 Aug 26;12:5123. doi: 10.1038/s41467-021-25107-3 (PMC8390644; doi:10.1038/s41467-021-25107-3)
Supplement: Supplementary file 1 — Supplementary Information [file 41467_2021_25107_MOESM1_ESM.pdf]

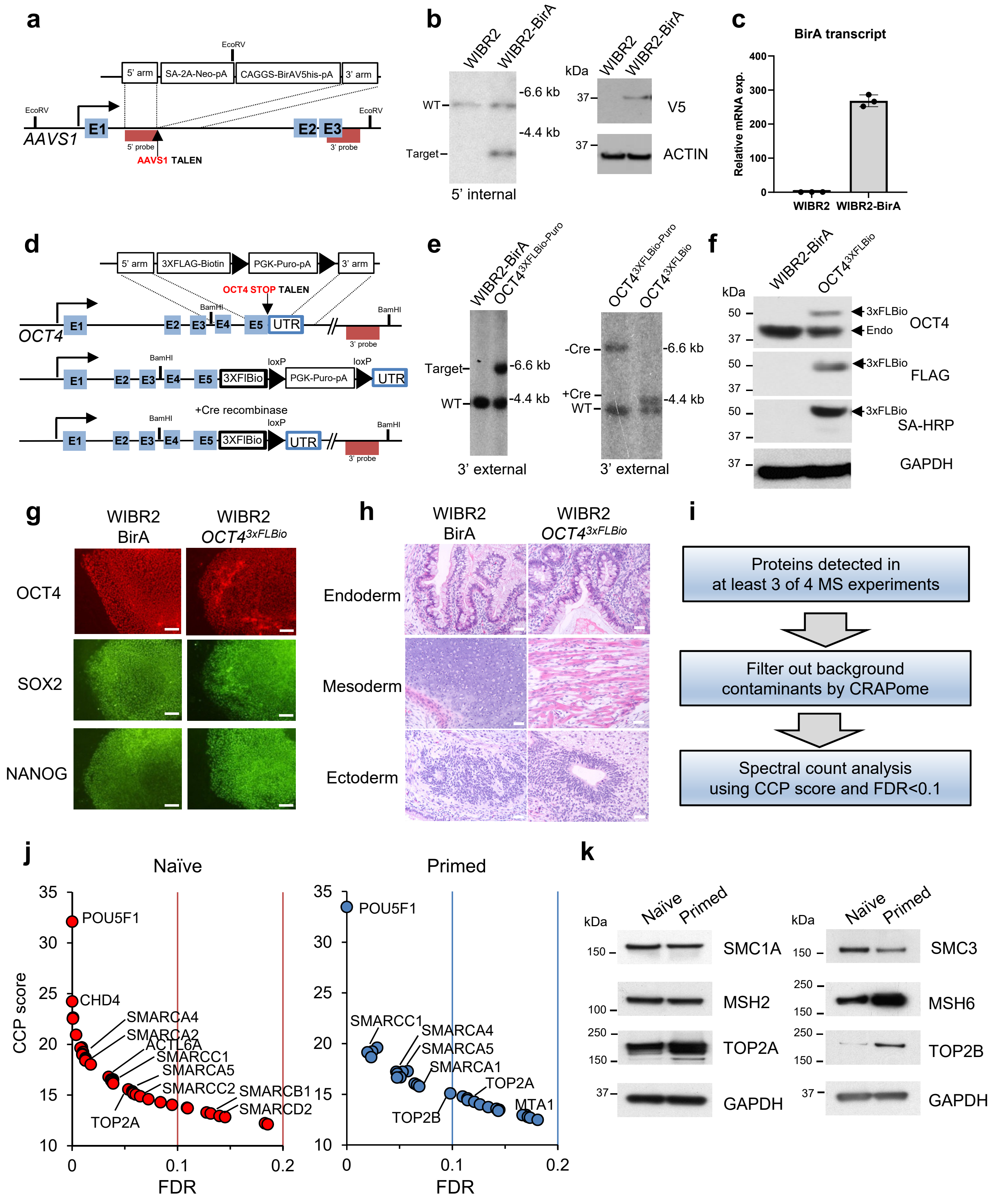
a
b
c
d
e
f
g
h
i
j
k
AAVS1
OCT4
AAVS1 TALEN
OCT4 STOP TALEN
5' arm
SA-2A-Neo-pA
CAGGS-BirAV5his-pA
3' arm
3XFLAG-Biotin
PGK-Puro-pA
+Cre recombinase
WIBR2
WIBR2-BirA
5' internal
3' external
V5
ACTIN
BirA transcript
Relative mRNA exp.
OCT4
FLAG
SA-HRP
GAPDH
SOX2
NANOG
Endoderm
Mesoderm
Ectoderm
Proteins detected in at least 3 of 4 MS experiments
Filter out background contaminants by CRAPome
Spectral count analysis using CCP score and FDR<0.1
Naïve
Primed
CCP score
FDR
SMC1A
SMC3
MSH2
MSH6
TOP2A
TOP2B

Suppl. Fig. 1

Supplementary Fig. 1 | An OCT4 interactome in human ESCs. a, Strategy for TALEN-mediated targeting of a CAGGS-BirA-V5His transgene to the *AAVS1* safe harbor locus in primed hESCs. **b**, Southern blot analysis (left) confirming targeting of the CAGGS-BirA-V5His transgene to *AAVS1* locus in WIBR2 hESCs. EcoRV-digested genomic DNA was hybridized with a 5' internal probe. Expected fragment size: wild type = 5.6 kb, targeted = 3.5 kb. Western blot analysis (right) with anti-V5 antibody confirming expression of BirA-V5His transgene in correctly targeted WIBR2 hESCs. **c**, Quantitative gene expression analysis for BirA expression in WIBR2 parental and WIBR2-BirA hESCs. Data are presented as mean ± SD, obtained from n=3 technical replicates. **d**, Strategy for TALEN-mediated targeting of a 3xFLAG-Biotin sequence to the STOP codon of endogenous *OCT4* in human ESCs. Donor vector contains a puromycin (Puro) selection cassette that can be removed upon transient expression of Cre recombinase. **e**, Southern blot analysis confirming excision of the PGK-Puro-pA selection cassette from correctly targeted WIBR2-$OCT4^{3xFLBio}$ hESCs. BamHI-digested genomic DNA was hybridized with a 3' external probe. Expected fragment size: wild type = 4.2 kb, targeted = 6.2 kb, Cre-excised = 4.4 kb. **f**, Western blot analysis with anti-OCT4, anti-3xFLAG, SA-HRP (anti-Biotin), and anti-GAPDH antibodies confirming expression of OCT4-3xFLBio fusion protein. **g**, Immunofluorescence staining for OCT4, SOX2 and NANOG in WIBR2-BirA and WIBR2-$OCT4^{3xFLBio}$ hESCs. Scale bar is 100 µm. **h,** Teratomas generated from WIBR2-BirA and WIBR2-$OCT4^{3xFLBio}$ hESCs. Representative tissues of the three germ layers are indicated. Scale bar is 50 µm.. **i**, Pipeline for identifying OCT4-associated proteins. **j**, Scatter plots of CCP score versus FDR value for the OCT4-associated proteins identified by AP-MS. The significance cutoff is set as FDR<0.1. **k**, Western blot analysis of OCT4-associated proteins in naïve and primed hESCs.

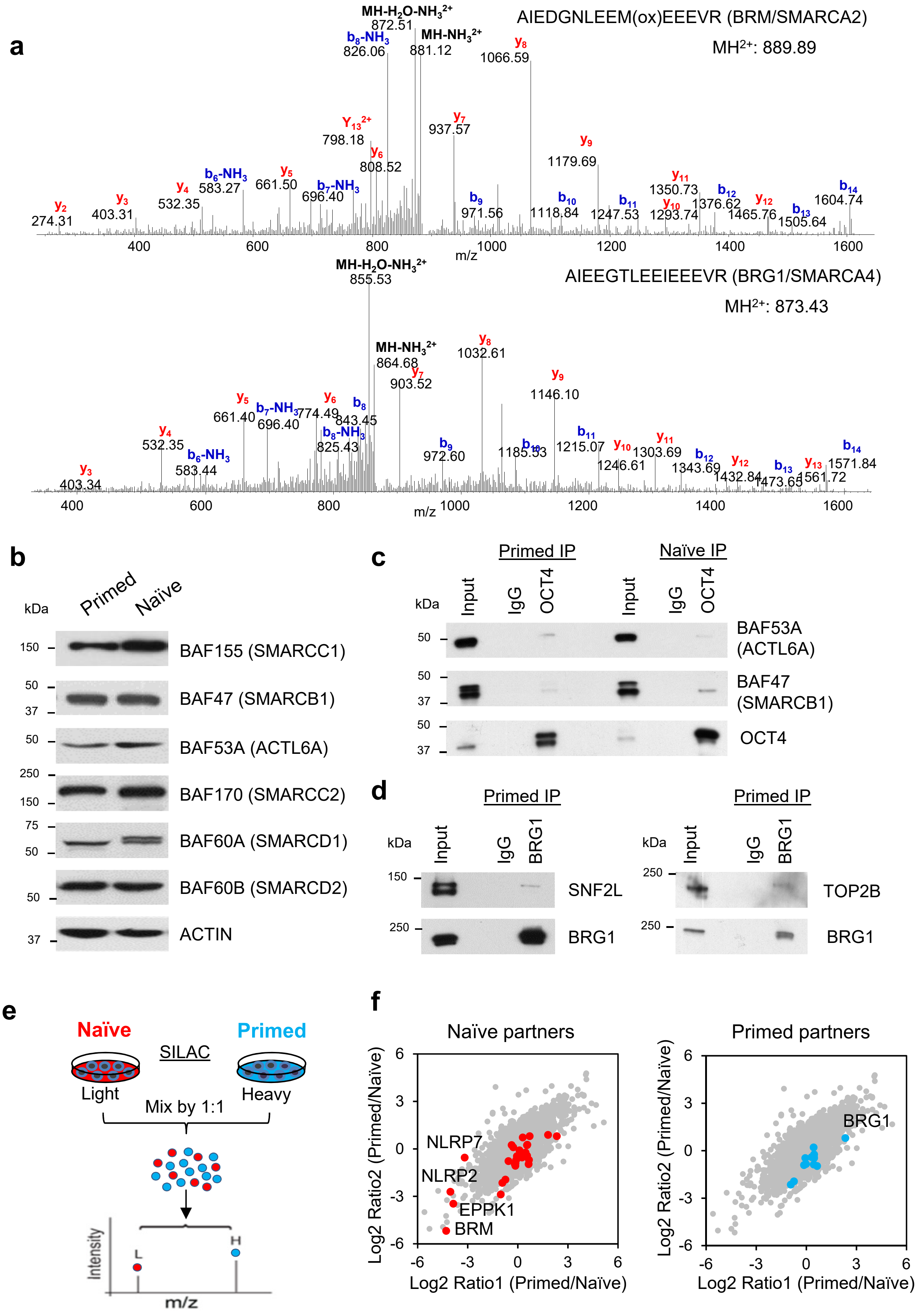

AIEDGNLEEM(ox)EEEVR (BRM/SMARCA2)
MH2+: 889.89
AIEEGTLEEIEEEVR (BRG1/SMARCA4)
MH2+: 873.43
m/z
Primed
Naïve
kDa
BAF155 (SMARCC1)
BAF47 (SMARCB1)
BAF53A (ACTL6A)
BAF170 (SMARCC2)
BAF60A (SMARCD1)
BAF60B (SMARCD2)
ACTIN
Primed IP
Naïve IP
Input
IgG
OCT4
BAF53A (ACTL6A)
BAF47 (SMARCB1)
OCT4
BRG1
SNF2L
TOP2B
Naïve
Primed
SILAC
Light
Heavy
Mix by 1:1
Intensity
m/z
Naïve partners
Primed partners
NLRP7
NLRP2
EPPK1
BRM
Log2 Ratio1 (Primed/Naïve)
Log2 Ratio2 (Primed/Naïve)

Suppl. Fig. 2

Supplementary Fig. 2 | MS identification and protein expression of BAF components. a, MS/MS spectra for identification of two homologous sequences in BRM (SMARCA2) (amino acids 1328~1342, AIEDGNLEEMEEEVR) and BRG1 (SMARCA4) (amino acids 1391~1405, AIEEGTLEEIEEEVR) from the OCT4 AP-MS data in hESCs. The b- and y-fragment ions are annotated. **b**, Western blot analysis of BAF and ISWI component proteins in primed and naïve hESCs. **c**, Co-immunoprecipitation (co-IP) of OCT4 and Western blot analysis of BAF components BAF47 (SMARCB1) and BAF53A (ACTL6A) in naïve and primed hESCs. **d**, BRG1 co-IP and western blot analysis of SNF2L (left) and TOP2B (right) in primed hESCs. **b-d**, Experiment is repeated independently twice with similar results. **e**, Strategy for stable isotope labeling of amino acids in culture (SILAC) and protein quantification by mass spectrometry of two pairs of isogenic primed and naïve hESC lines (WIBR2 and WIBR3). **f**, Scatter plots showing the heavy-versus-light (H/L) ratio distributions of all proteins (grey dots), and OCT4-associated proteins identified in naïve (left, red dots) and primed (right, blue dots) hESCs from the SILAC quantitation.

a

Cluster	Peak occupancy	Annotation
1	Naïve-specific OCT4, H3K27ac and BAF complex	Naïve-specific active enhancers
2	Primed-specific H3K4me3, H3K27me3, but no OCT4	Primed-specific bivalent promoters
3	Naïve/Primed-shared H3K4me3, but no OCT4 or H3K27ac	Naïve/Primed-shared silent promoters
4	Naïve/Primed-shared OCT4, H3K4me3, H3K27ac	Naïve/Primed-shared active promoters (OCT4-dependent)
5	Regions with only BAF complex	Unknown/random regions
6	Primed-specific OCT4, H3K27ac and BAF complex	Primed-specific active enhancers
7	Naïve/Primed-shared H3K4me3 and H3K27me3	Naïve/Primed-shared bivalent promoters
8	Naïve/Primed-shared OCT4, H3K27ac and BAF complex	Naïve/Primed-shared active enhancers
9	Naïve/Primed-shared H3K4me3 and H3K27ac, but no OCT4	Naïve/Primed-shared active promoters (OCT4-independent)
10	Naïve-specific OCT4, H3K4me3, H3K27ac and BAF complex	Naïve-specific active promoters

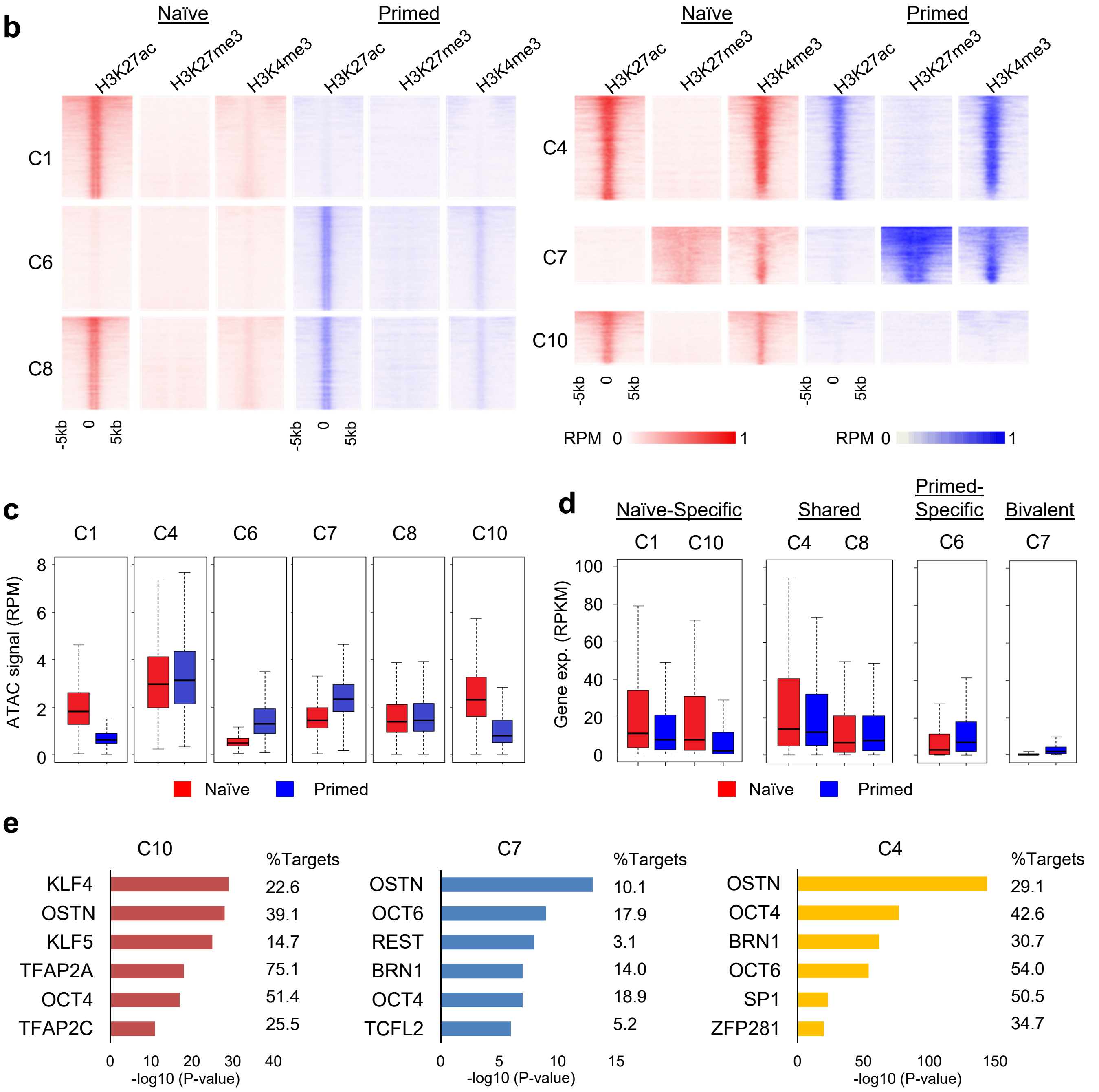

Suppl. Fig. 3

Supplementary Fig. 3 | Annotation of K-means clusters as naïve- and primed-specific enhancers. a, Annotation of the 10 K-means clusters identified in Fig. 3f. **b-d**, Intensity heatmaps (**b**) of histone H3 marks H3K27ac, H3K27me3, and H3K4me3 signals, ATAC-seq intensities (**c**), and target gene expression (**d**) at clusters C1, C4, C6, C7, C8, and C10 regions in naïve and primed hESCs. **c,d**, Boxplot presents the 25th, median, and 75th quartiles, and the whiskers extend 1.5 of interquartile ranges. Data are obtained from n=4 (**c**) and n=2 (**d**) biologically independent experiments, respectively. **e**, Top enrichment motifs from motif analysis at clusters C10, C7, and C4 regions, which are annotated as naïve-specific, naïve/primed shared bivalent, and naïve/primed shared active promoters, respectively. OSTN: OCT4/SOX2/TCF/NANOG consensus motif.

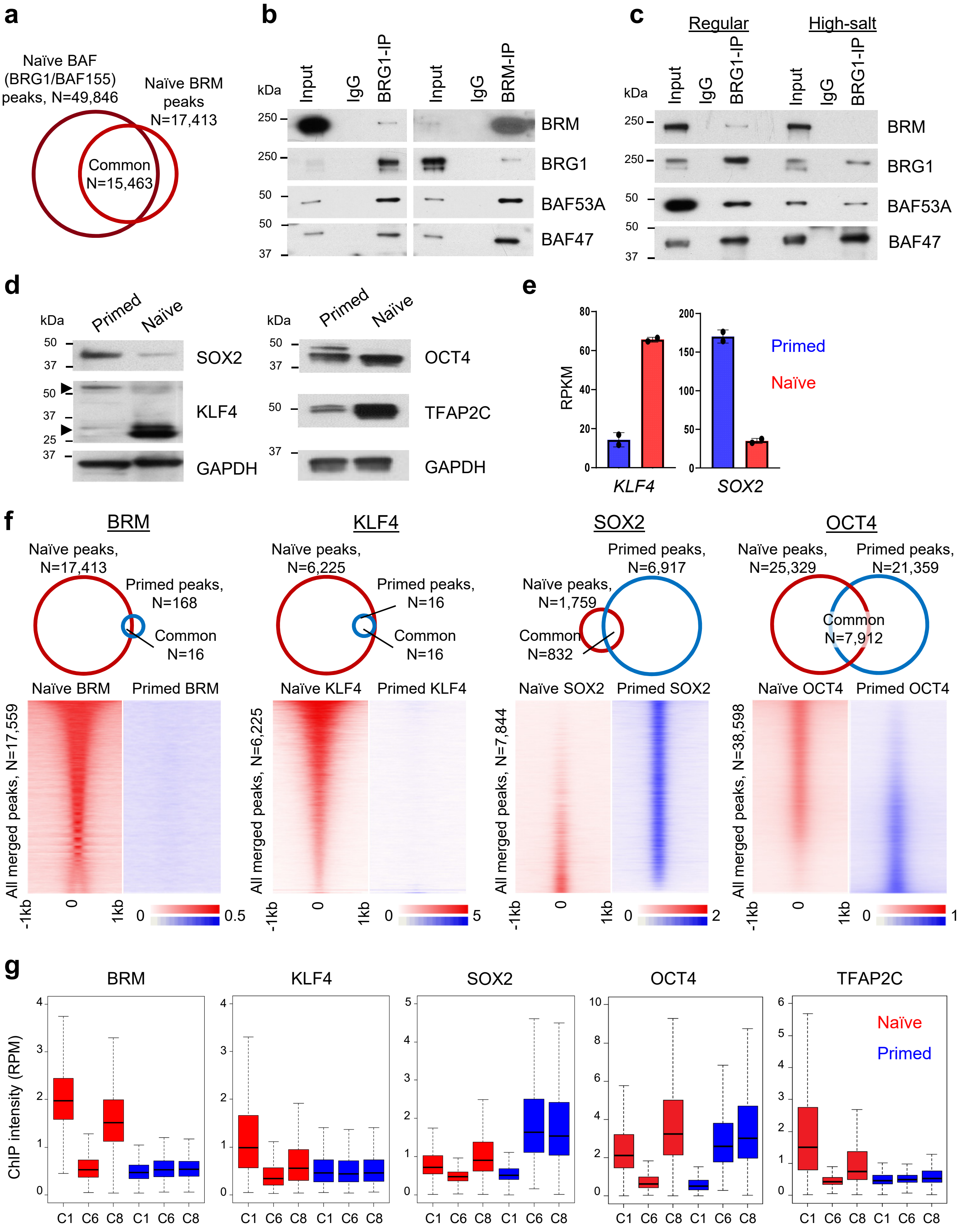
a
Naïve BAF (BRG1/BAF155) peaks, N=49,846
Naïve BRM peaks N=17,413
Common N=15,463
b
kDa
Input
IgG
BRG1-IP
Input
IgG
BRM-IP
250
250
50
50
37
BRM
BRG1
BAF53A
BAF47
c
Regular
High-salt
kDa
Input
IgG
BRG1-IP
Input
IgG
BRG1-IP
250
250
50
50
37
BRM
BRG1
BAF53A
BAF47
d
kDa
Primed
Naïve
50
37
SOX2
50
37
25
KLF4
37
GAPDH
kDa
Primed
Naïve
50
37
OCT4
50
TFAP2C
37
GAPDH
e
RPKM
KLF4
SOX2
Primed
Naïve
f
BRM
Naïve peaks, N=17,413
Primed peaks, N=168
Common N=16
Naïve BRM
Primed BRM
All merged peaks, N=17,559
KLF4
Naïve peaks, N=6,225
Primed peaks, N=16
Common N=16
Naïve KLF4
Primed KLF4
All merged peaks, N=6,225
SOX2
Primed peaks, N=6,917
Naïve peaks, N=1,759
Common N=832
Naïve SOX2
Primed SOX2
All merged peaks, N=7,844
OCT4
Naïve peaks, N=25,329
Primed peaks, N=21,359
Common N=7,912
Naïve OCT4
Primed OCT4
All merged peaks, N=38,598
-1kb
0
1kb
0
0.5
5
2
1
g
BRM
KLF4
SOX2
OCT4
TFAP2C
ChIP intensity (RPM)
C1 C6 C8 C1 C6 C8
Naïve
Primed

Suppl. Fig. 4

Supplementary Fig. 4 | Genome-wide location analysis for pluripotency-associated TFs in naïve and primed hESCs. a, Overlap of BRM and BAF (BRG1/BAF155) ChIP-seq peaks in naïve hESCs. **b**, Reciprocal co-IP of BRM and BRG1 with other BAF components in naïve hESCs. **c**, Co-IP of BRG1 and Western analysis of BRM and other BAF components in regular and high-salt conditions. **d**, Protein expression of SOX2, KLF4, OCT4, and TFAP2C in naïve and primed hESCs. Note that a strong KLF4 band is observed at about 28 kDa in naïve hESCs, which differs from the canonical molecular weight of KLF4 protein at 55 kDa. **b-d,** Experiment is repeated independently twice with similar results. **e**, Expression of KLF4 and SOX2 mRNA from RNA-seq analysis in naïve and primed hESCs. Data are presented as mean ± SD, obtained from n=2 biologically independent experiments. **f**, Overlapping peaks and intensity heatmaps for BRM, KLF4, OCT4, SOX2 in naïve and primed hESCs. **g**, Intensity plots of BRM, KLF4, SOX2, OCT4, and TFAP2C at clusters C1, C6, and C8 regions in naïve (red) and primed (blue) hESCs. Boxplot presents the 25th, median, and 75th quartiles, and the whiskers extend 1.5 of interquartile ranges.

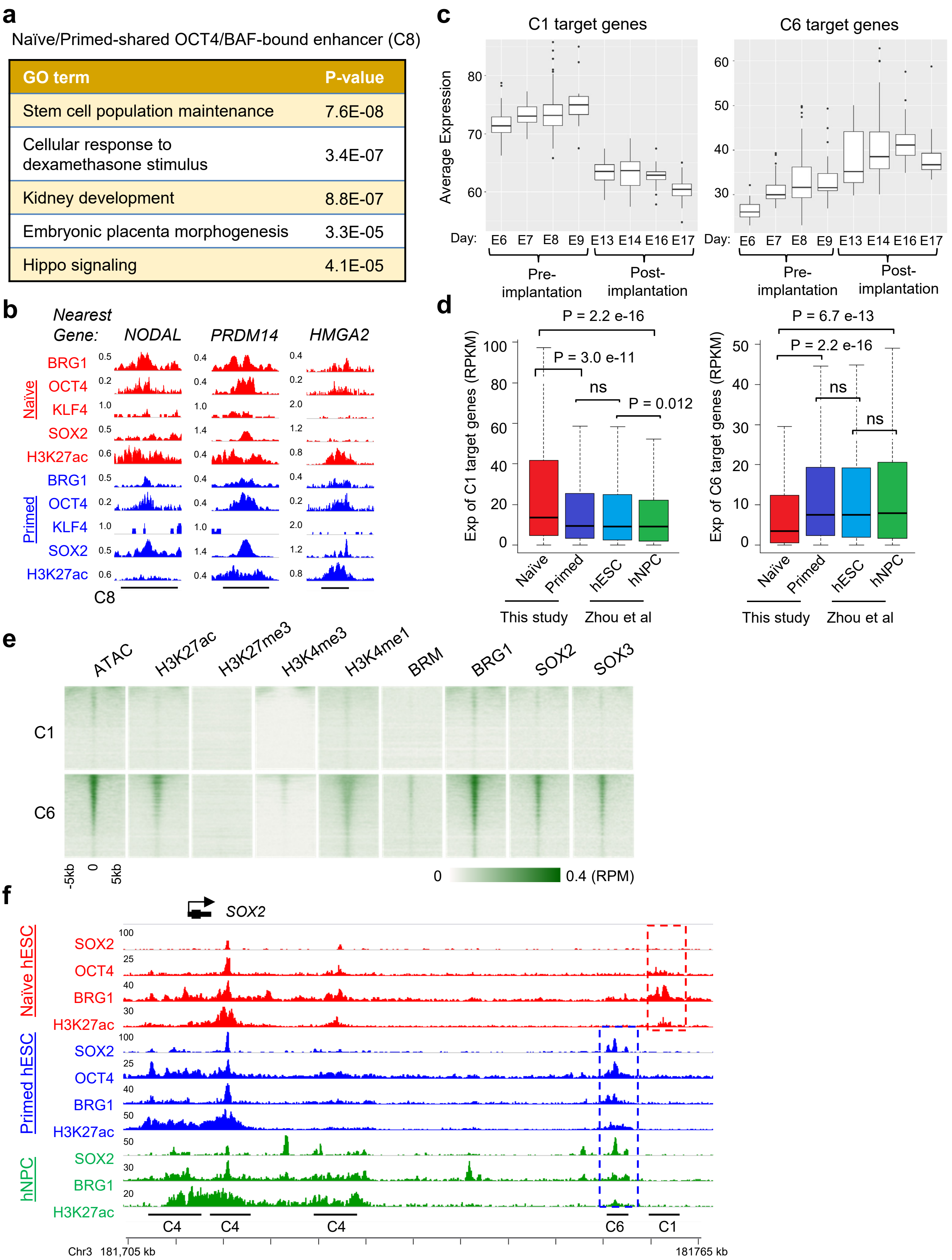
a
Naïve/Primed-shared OCT4/BAF-bound enhancer (C8)
GO term | P-value
Stem cell population maintenance | 7.6E-08
Cellular response to dexamethasone stimulus | 3.4E-07
Kidney development | 8.8E-07
Embryonic placenta morphogenesis | 3.3E-05
Hippo signaling | 4.1E-05
b
Nearest Gene:
NODAL
PRDM14
HMGA2
Naïve
Primed
BRG1
OCT4
KLF4
SOX2
H3K27ac
C8
c
C1 target genes
C6 target genes
Average Expression
Day: E6 E7 E8 E9 E13 E14 E16 E17
Pre-implantation
Post-implantation
d
Exp of C1 target genes (RPKM)
Exp of C6 target genes (RPKM)
P = 2.2 e-16
P = 3.0 e-11
ns
P = 0.012
P = 6.7 e-13
P = 2.2 e-16
Naïve
Primed
hESC
hNPC
This study
Zhou et al
e
ATAC
H3K27ac
H3K27me3
H3K4me3
H3K4me1
BRM
BRG1
SOX2
SOX3
C1
C6
-5kb
0
5kb
0
0.4 (RPM)
f
SOX2
Naïve hESC
Primed hESC
hNPC
C4
C6
C1
Chr3 181,705 kb
181765 kb

Suppl. Fig. 5

Supplementary Fig. 5 | Functional annotation of naïve/primed-shared (C8) enhancers and activity of primed-specific (C6) enhancers in hNPCs. **a-b**, GO analysis (**a**), ChIP-seq tracks (**b**) of representative target genes (*NODAL, PRDM14*, and *HMGA2*) with naïve/primed-shared OCT4/BAF-bound enhancers (C8). **c**, Expression of target genes with naïve- (C1) and primed-specific (C6) enhancers during pre-implantation and post-implantation development in non-human primates[1]. At least 20 cells were analyzed at each of the indicated timepoints. **d**, Expression of C1 and C6 target genes in naïve versus primed hESCs in this study compared to that in hESCs (primed) versus hNPCs from Zhou et al.[2] P-value is from two-sided Mann-Whitney test. Data are obtained from n=2 biologically independent experiments. **c,d**, Boxplot presents the 25th, median, and 75th quartiles, and the whiskers extend to the 1.5 of interquartile ranges. **e**, Intensity heatmaps for ATAC-seq, histone marks, BAF components and SOX TFs in hNPCs[3] at naïve- (C1) and primed-specific (C6) enhancers. **f**, ChIP-seq tracks of OCT4, SOX2, BRG1, and H3K27ac at *SOX2* locus in naïve and primed hESCs (this study) as well as in hNPCs[3].

a

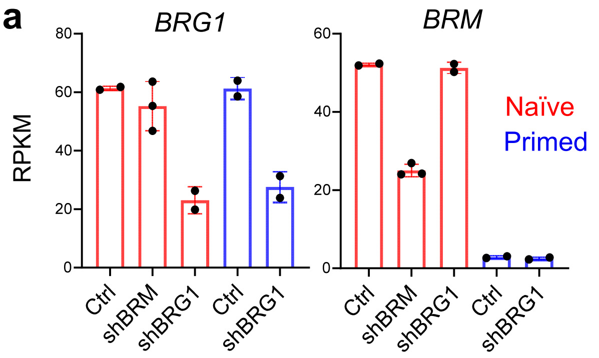

b

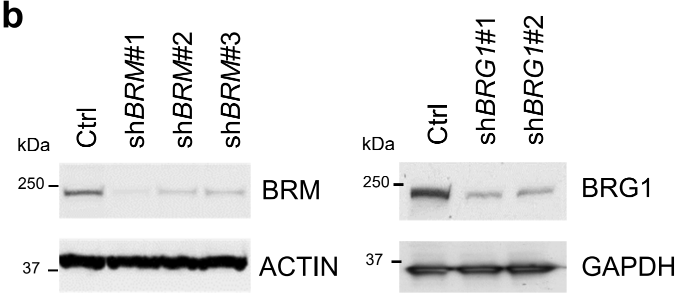

c

Genes downregulated in primed sh*BRG1* (N=101)

GO term	P-value
Immune response	0.0015
Xenobiotic metabolic process	0.0077
Steroid metabolic process	0.0209
Nucleosome assembly	0.0239
T cell receptor signaling pathway	0.0407

Genes upregulated in primed sh*BRG1* (N=94)

GO term	P-value
Extracellular matrix organization	0.0001
Cell fate commitment	0.0008
Regulation of cell migration	0.0032
Endodermal cell fate determination	0.0080
Cardiac cell fate determination	0.0159
Cell migration involved in gastrulation	0.0315
Post-embryonic development	0.0344

d

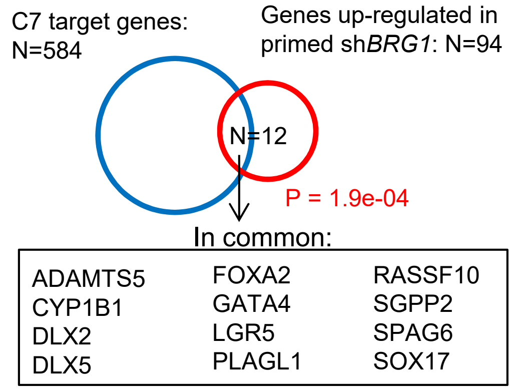

e

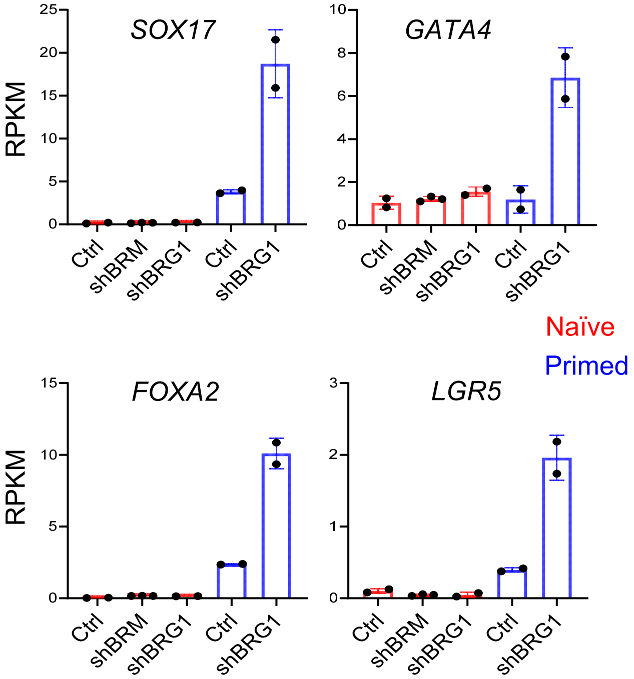

f

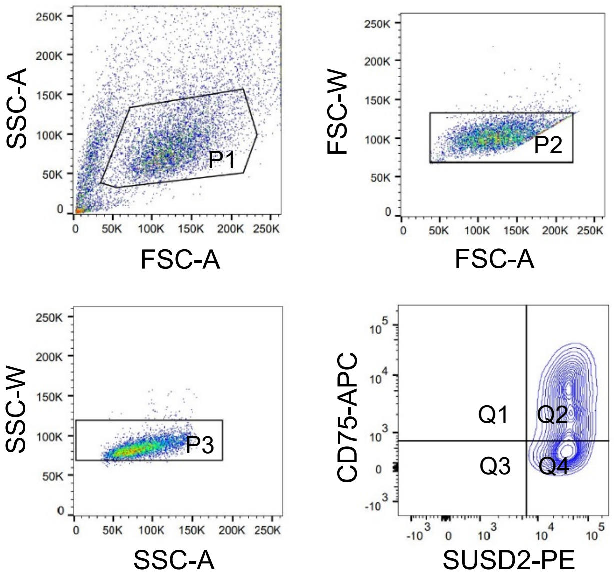

Suppl. Fig. 6

Supplementary Fig. 6 | Loss-of-function studies of BRG1 and BRM in naïve and primed hESCs. **a**, Expression of *BRG1* and *BRM* mRNA transcripts upon shRNA-mediated KD in both naïve and primed hESCs. Data are presented as mean ± SD. **b**, Western blot analysis of naïve hESCs treated with control or *BRM*-targeted shRNAs, and primed hESCs treated with control or *BRG1*-targeted shRNAs. ACTIN or GAPDH was used as a loading control. KD was confirmed by n=3 (*shBRM*) and n=2 (*shBRG1*) independent shRNAs. **c**, GO analysis of significantly up- and down-regulated genes upon *BRG1* KD (sh*BRG1*) in primed hESCs. **d**, Overlap of naïve/primed-shared bivalent promoter (C7) target genes with the genes significantly upregulated upon *BRG1* KD in primed ESCs. P-value is from the right-sided Fisher's Extract test. **e**, Expression of endodermal marker genes *SOX17, GATA4, FOXA2,* and *LGR5* upon KD of *BRG1* or *BRM* in naïve and primed hESCs. Data are presented as mean ± SD. **f**, Representative gating strategy for flow cytometry analysis for expression of naïve-specific cell-surface markers CD75 and SUSD2 (see Fig. 6f and Fig. 7c).

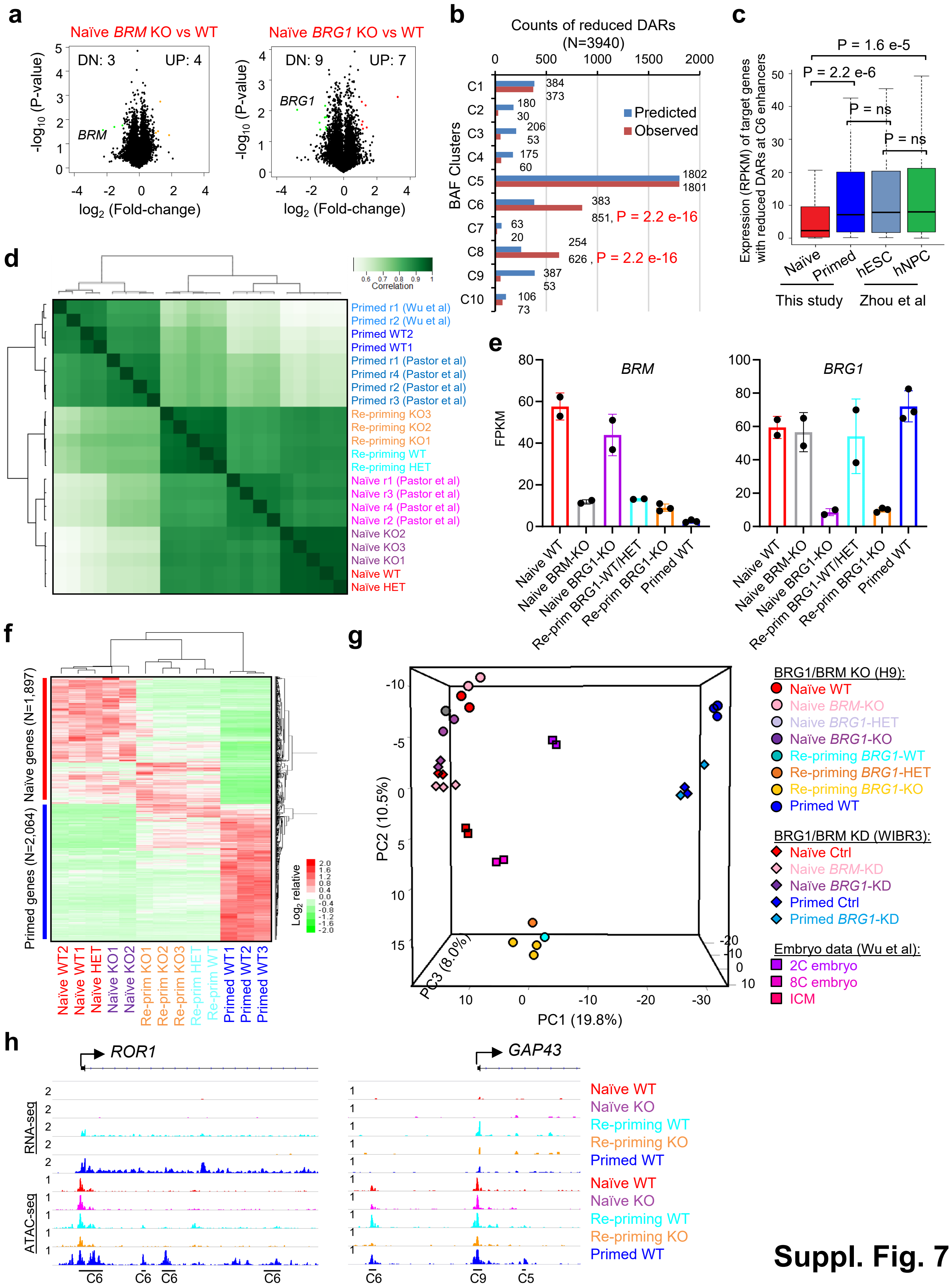
a
Naïve BRM KO vs WT
DN: 3
UP: 4
BRM
-log10 (P-value)
log2 (Fold-change)
Naïve BRG1 KO vs WT
DN: 9
UP: 7
BRG1
b
Counts of reduced DARs (N=3940)
BAF Clusters
Predicted
Observed
P = 2.2 e-16
P = 2.2 e-16
c
Expression (RPKM) of target genes with reduced DARs at C6 enhancers
P = 1.6 e-5
P = 2.2 e-6
P = ns
P = ns
Naïve
Primed
hESC
hNPC
This study
Zhou et al
d
Correlation
Primed r1 (Wu et al)
Primed r2 (Wu et al)
Primed WT2
Primed WT1
Primed r1 (Pastor et al)
Primed r4 (Pastor et al)
Primed r2 (Pastor et al)
Primed r3 (Pastor et al)
Re-priming KO3
Re-priming KO2
Re-priming KO1
Re-priming WT
Re-priming HET
Naïve r1 (Pastor et al)
Naïve r3 (Pastor et al)
Naïve r4 (Pastor et al)
Naïve r2 (Pastor et al)
Naïve KO2
Naïve KO3
Naïve KO1
Naïve WT
Naïve HET
e
BRM
BRG1
FPKM
Naive WT
Naive BRM-KO
Naive BRG1-KO
Re-prim BRG1-WT/HET
Re-prim BRG1-KO
Primed WT
f
Naive genes (N=1,897)
Primed genes (N=2,064)
Log2 relative
Naive WT2
Naive WT1
Naive HET
Naive KO1
Naive KO2
Re-prim KO1
Re-prim KO2
Re-prim KO3
Re-prim HET
Re-prim WT
Primed WT1
Primed WT2
Primed WT3
g
PC1 (19.8%)
PC2 (10.5%)
PC3 (8.0%)
BRG1/BRM KO (H9):
Naïve WT
Naive BRM-KO
Naive BRG1-HET
Naïve BRG1-KO
Re-priming BRG1-WT
Re-priming BRG1-HET
Re-priming BRG1-KO
Primed WT
BRG1/BRM KD (WIBR3):
Naïve Ctrl
Naive BRM-KD
Naïve BRG1-KD
Primed Ctrl
Primed BRG1-KD
Embryo data (Wu et al):
2C embryo
8C embryo
ICM
h
ROR1
GAP43
RNA-seq
ATAC-seq
Naïve WT
Naïve KO
Re-priming WT
Re-priming KO
Primed WT
C6
C9
C5

Suppl. Fig. 7

Supplementary Fig. 7 | Transcriptional and epigenomic analyses of *BRG*$^{-/-}$ naïve hESCs. a, Volcano plots showing relatively few differentially expressed genes (fold-change>2, p-value<0.05) between *BRM* KO *(BRM*$^{-/-}$*)* and WT (left), and *BRG1* KO *(BRG1*$^{-/-}$*)* and WT (right) naïve hESCs. **b**, Predicted and observed number of reduced ATAC DARs (N=3,940, as indicated in Fig. 7f) on day 4 of re-priming in *BRG1* KO cells at BAF-bound clusters C1-10 (as defined in Fig. 3f). Only C6 and C8 have higher observed counts, and P-value is from the right-sided Fisher's Extract test. **c,** Expression (RPKM) of the target genes with decreased ATAC signal at C6 enhancers in naïve versus primed hESCs in this study compared to that in hESCs (primed) versus hNPCs from Zhou et al.[2] P-value is from two-sided Mann-Whitney test. Boxplot presents the 25th, median, and 75th quartiles, and the whiskers extend to the 1.5 of interquartile ranges. Data are obtained from n=2 biologically independent experiments. **d**, Heatmaps for the correlation of ATAC-seq data from *BRG1* WT, HET, and KO cells on day 4 of re-priming compared to naïve and primed hESCs. Previously published naïve and primed ATAC-seq data are included for comparison[4, 5]. **e**, Expression of *BRM* and *BRG1* mRNA transcripts from the RNA-seq analysis of naïve, primed, and re-priming hESCs. Data are presented as mean ± SD, obtained from n=3 biologically independent experiments. **f**, Unbiased hierarchical clustering of RNA-seq data for *BRG1* WT, HET and KO cells on day 5 of re-priming based on naïve-specific genes (N=1,897) and primed-specific genes (N=2,064), as defined in Fig. 7i. **g,** PCA analysis of RNA-seq data from both *BRM/BRG1* knockout (KO) and knockdown (KD) cells under naïve, primed, and re-priming conditions (this study) compared to bulk RNA-seq analyses of human embryos[4]. **h,** the RNA-seq and ATAC-seq tracks at the *ROR1* and *GAP43* loci showing reduced expression and chromatin accessibility at enhancer and promoter regions in *BRG1* KO cells during re-priming.

SUPPLEMENTARY REFERENCES

1. Nakamura, T. *et al.* A developmental coordinate of pluripotency among mice, monkeys and humans. *Nature* **537**, 57-62 (2016).
2. Zhou, C. *et al.* Comprehensive profiling reveals mechanisms of SOX2-mediated cell fate specification in human ESCs and NPCs. *Cell Res* **26**, 171-189 (2016).
3. Gao, F. *et al.* Heterozygous Mutations in SMARCA2 Reprogram the Enhancer Landscape by Global Retargeting of SMARCA4. *Mol Cell* **75**, 891-904 e897 (2019).
4. Wu, J. *et al.* Chromatin analysis in human early development reveals epigenetic transition during ZGA. *Nature* **557**, 256-260 (2018).
5. Pastor, W.A. *et al.* TFAP2C regulates transcription in human naive pluripotency by opening enhancers. *Nat Cell Biol* **20**, 553-564 (2018).
