# Supplementary material for: OCT4 cooperates with distinct ATP-dependent chromatin remodelers in naïve and primed pluripotent states in human
Source: Nat Commun. 2021 Aug 26;12:5123. doi: 10.1038/s41467-021-25107-3 (PMC8390644; doi:10.1038/s41467-021-25107-3)
Supplement: Supplementary file 2 — Description of Additional Supplementary Files [file 41467_2021_25107_MOESM2_ESM.docx]

**Description of Additional Supplementary Files**

**Supplementary Data 1**. List of OCT4 interactome proteins in naïve and primed hESCs.

**Supplementary Data 2**. Summary of QC metrics of ChIP-seq and CUT&Tag data.

**Supplementary Data 3**. Genome-wide locations of all BAF peaks (N=69,007) and their annotations.

**Supplementary Data 4**. Summary of RNA-seq gene expression data from BRG1/BRM knockdown experiment. P-value is from the unpaired two-sided T-test.

**Supplementary Data 5**. Summary of RNA-seq gene expression data from BRG1/BRM knockout and re-priming experiment. P-value is from the unpaired two-sided T-test.

**Supplementary Data 6**. Summary of primers, sequencing data from public resources, and sequencing data generated in this study.
