# Supplementary material for: OCT4 cooperates with distinct ATP-dependent chromatin remodelers in naïve and primed pluripotent states in human
Source: Nat Commun. 2021 Aug 26;12:5123. doi: 10.1038/s41467-021-25107-3 (PMC8390644; doi:10.1038/s41467-021-25107-3)
Supplement: Supplementary file 9 — Source Data ZIP file [file 41467_2021_25107_MOESM9_ESM.zip › Source data-ChIP QC metrics.pdf]

QC metrics of ChIP-seq data

We provide the QC metrics of our ChIP-seq and CUT&Tag data in Supplementary Data 2. In addition, as shown in the table below, the critical QC metrics are comparable or even better to the ENCODE recommended QC standard for ChIP-seq (https://www.encodeproject.org/chip-seq/transcription-factor-encode4/). Such high-quality ChIP-seq data allow us perform downstream analyses with high confidence as presented in our manuscript:

	Assay Type	Total reads	Non-redundant uniquely_mapped reads	Random 20M_reads*	Uniquely mapped reads	Uniquely mapped ratio	Non-redundant uniquely_mapped reads	PBC1	PBC2
Naive_155	ChIP-seq	70266227	40,645,340	20,000,000	17306559	**86.53%**	15396255	**0.89**	**11.89**
Naive_BRG	ChIP-seq	71479139	39,120,543	20,000,000	17321451	**86.61%**	15113033	**0.87**	**11.02**
Naive_BRM	ChIP-seq	41246231	29,478,799	20,000,000	18235695	**91.18%**	16125948	**0.88**	**16.72**
Primed_155	ChIP-seq	74107781	42,979,122	20,000,000	19712575	**98.56%**	17799135	**0.90**	**9.18**
Primed_BRG	ChIP-seq	77486295	51,980,567	20,000,000	19667547	**98.34%**	17613848	**0.90**	**13.32**
**Averaged ChIP-seq QC:**						**79.06%**		**0.86**	**8.46**
ENCODE recommendation: compliant standard	ChIP-seq		10,000,000			**80%-90%**		**0.8-0.9**	**3-10**
ENCODE recommendation: Ideal standard	ChIP-seq		>20,000,000			**>90%**		**>0.9**	**>10**

The QC metrics were measured based on randomly selected 20M raw-reads, and yielded ~18.5M uniquely mapped reads, which were comparable to ENCODE recommendation.

Meanwhile, we carefully explored the ChIP-seq enrichment. First, we calculated the enrichment of BRG1 and BRM peaks in naïve and primed hESCs. As shown below, we see significant enrichment (BRG1, BRM ChIP-seq signal/input signal) of all the ChIP assays in our study, especially when compared to a random control in the genome (randomly selected regions with same size distribution as ChIP-seq peaks). These results indicate that our ChIP-seq assays have good enrichment of BRG1 and BRM binding and that the binding peaks identified in our study are reliable:

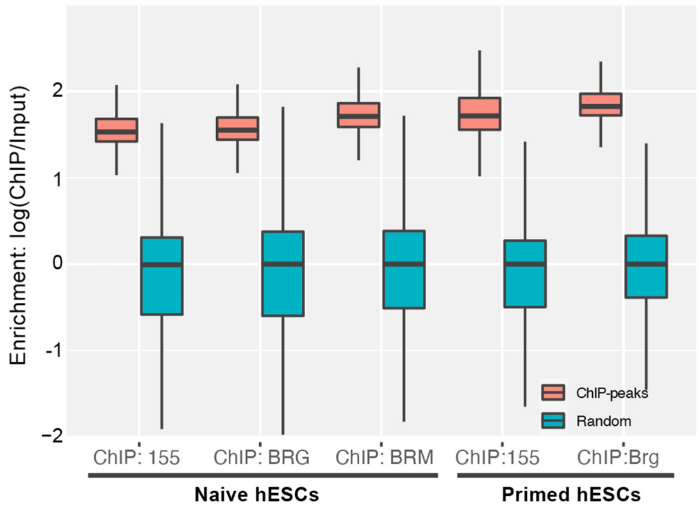

We examined the correlation between GC content and enrichment of all the ChIP-seq peaks in our study. We generated the scatter plots shown below in which GC content and enrichment of ChIP-seq peaks in both naïve and primed hESCs are plotted. The correlation coefficients are all close to 0, suggesting that the ChIP-seq peaks generated in our study are independent of GC content:

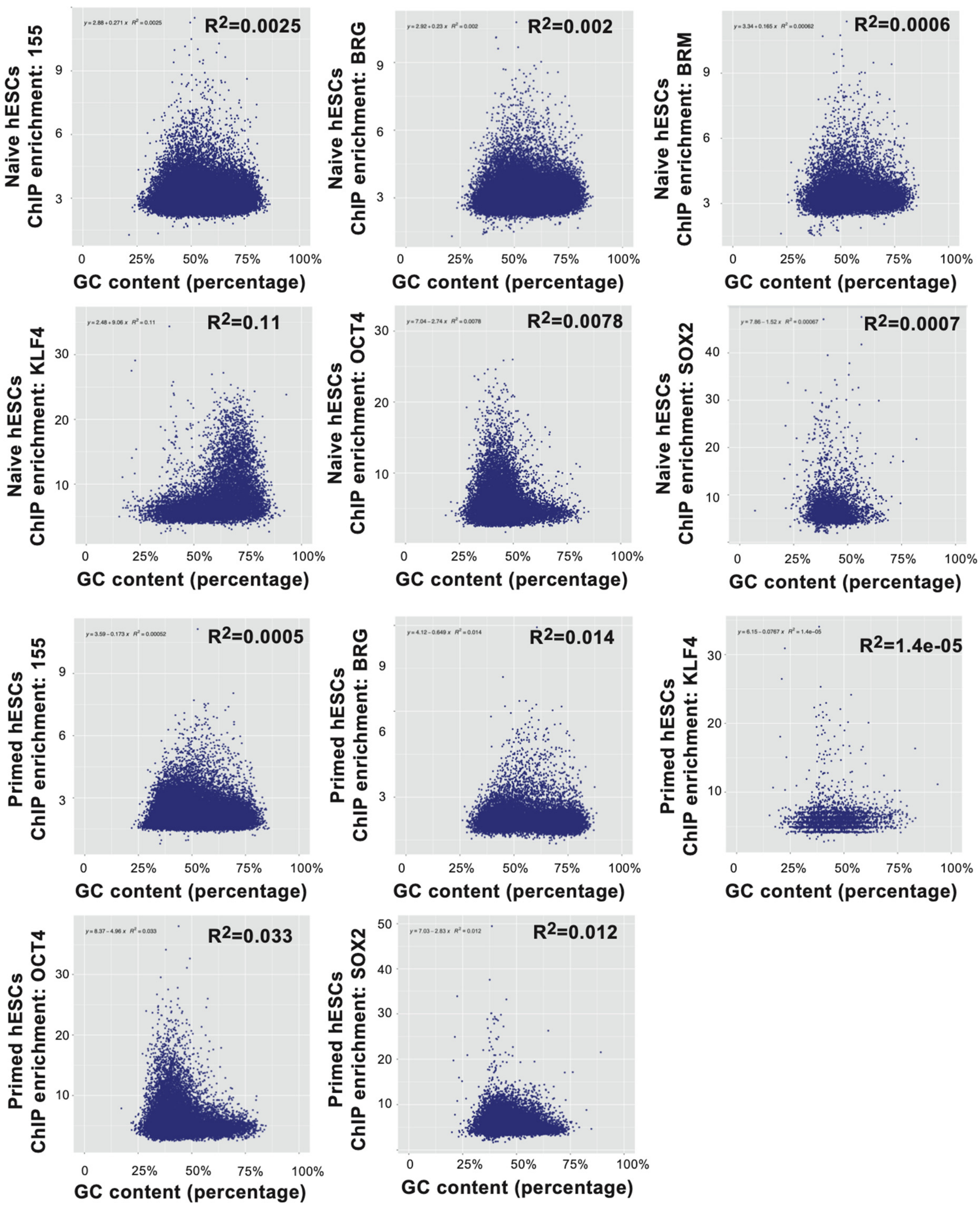

We explored the relationship between ATAC-seq data and ChIP-seq data. In naïve and primed hESCs, nearly 30% and 13% of BRG1 peaks, respectively, do not overlap with ATAC-seq peaks (as shown in the table below), indicating that BRG1 binding events may be independent of open chromatin status, and suggesting that BRG1 binding may induce open chromatin status during cell fate transitions:

	ATAC-seq peaks	ATAC-seq peaks overlapping BRG1 peaks	BRG1 peaks	BRG1 peaks without ATAC-seq peaks
Naïve ESC	127,833	46,928	61,839	14,911
Primed ESC	128,934	14,803	16,295	1,492

We also explored the signal density between BRG1 binding and ATAC-seq signal. We calculated the BRG1 ChIP-seq enrichment and ATAC-seq enrichment by using IgG input as control and plotted the enrichment based on overlap between BRG1 peaks and ATAC-seq peaks, as shown in the figure below. We can clearly observe BRG1 peaks that do not overlap with ATAC-seq peaks, since they exhibit highly enriched BRG1 signal but lower ATAC-seq enrichment. These results further suggest that BRG1 binding is independent of open chromatin status in both naïve and primed hESCs.

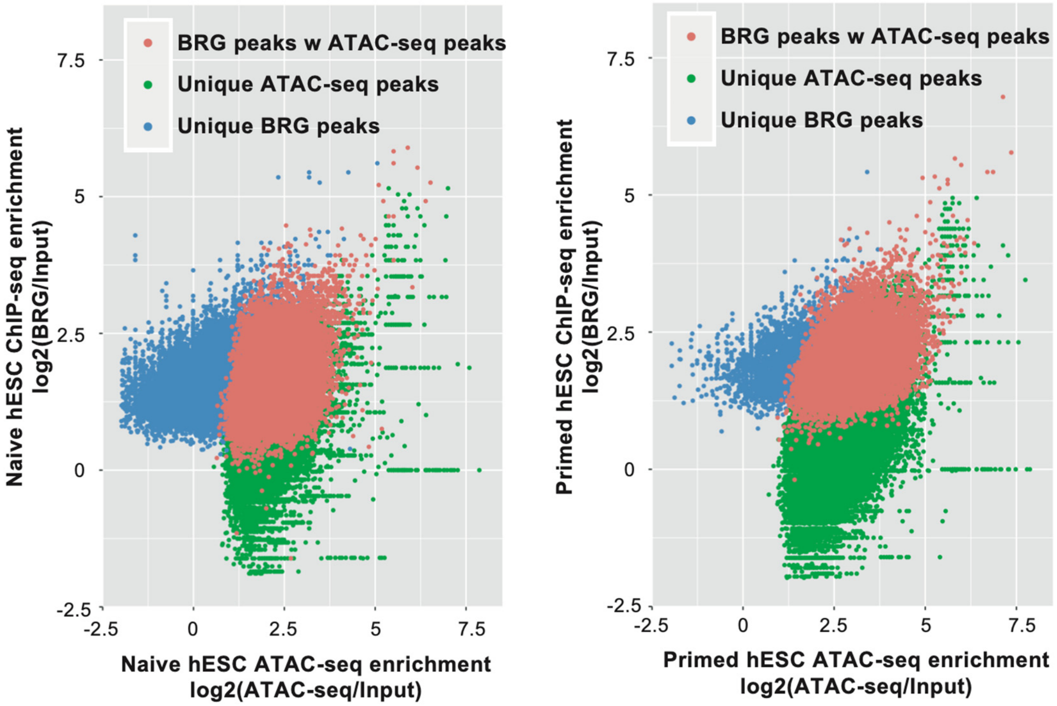

Finally, we performed motif scanning in the binding regions identified in OCT4 ChIP-seq, SOX2 ChIP-seq, and KLF4 CUT&Tag assays (as shown in the table below). By using FIMO with default parameter, in both naïve and primed hESCs we observed that ~60% of OCT4 peaks and SOX2 peaks contain OCT4 and SOX2 binding motifs, respectively. Similarly, 59% of KLF4 peaks in naïve hESC contain KLF4 binding motifs. We only identified 3,193 KLF4 binding peaks in primed hESCs and only 13% of them contain KLF4 binding motifs. This result suggests that KLF4 plays a more central role in gene regulation in the naïve pluripotent state.

ChIP	# of peaks	# of found motifs	# of unique Peaks with motif	% of peaks with motif
Naïve OCT4	17527	19335 (OCT4)	10625	0.6062075655
Primed OCT4	16989	18709 (OCT4)	9863	0.580552122
Naïve KLF4	11605	24266 (KLF4)	6802	0.5861266695
Primed KLF4	3193	778 (KLF4)	429	0.1343564046
Naïve SOX2	4259	4510 (SOX2)	2397	0.5628081709
Primed SOX2	16631	19296 (SOX2)	10592	0.6368829295

Methods

FIMO motif detection. Bedtools getfasta was used to extract DNA sequence under ChIP-seq peaks from human genome hg38 assemble for the downstream identification of transcription factor binding sites. Motif calling was performed using FIMO from the MEME suite with default parameters and using the HOCOMOCO database of motif sequence. Fraction of peaks containing a motif of interest was calculated as unique peaks containing the motif divided by total number of peaks.

ChIP enrichment vs GC content and vs random genomic region enrichment. For each ChIP-seq experiment, enrichment under peaks was determined by the callpeak function of macs2. Sequence under each peak was extracted using bedtools getfasta function, and fraction of C/G nucleotides (GC content) was further calculated. Enrichment vs GC content was plotted using the ggplot2 package in R for scatterplots and the ggmisc package to calculate best fit lines and R^2 values. Random genomic regions were selected by using the bedtools shuffle function to select regions with the same size distribution as the ChIP-seq peaks. Counts under these regions were quantified for ChIP and input libraries using the bedtools coverage function with option -counts and then CPM normalized by library depth. Enrichment was calculated as ChIP/input. Enrichment of ChIP-seq peaks vs random regions was plotted using ggplot2 in R.

ChIP enrichment vs ATAC enrichment. ChIP-seq and ATAC-seq peaks were both determined by the macs2 callpeak function using input samples as the control. Intersected peaks between ChIP and ATAC were called using the bedtools intersect function with options -f 0.25 -F 0.25 -e. Peaks unique to ChIP or ATAC were called using the bedtools subtract function with option -A to subtract the intersected peaks from the ChIP or ATAC peaks. Counts under each peak was quantified and normalized as described above for ChIP, ATAC, and input libraries. Enrichment was calculated as described above and plotted using the ggplot2 package in R.
