# Supplementary material for: OCT4 cooperates with distinct ATP-dependent chromatin remodelers in naïve and primed pluripotent states in human
Source: Nat Commun. 2021 Aug 26;12:5123. doi: 10.1038/s41467-021-25107-3 (PMC8390644; doi:10.1038/s41467-021-25107-3)
Supplement: Supplementary file 9 — Source Data ZIP file [file 41467_2021_25107_MOESM9_ESM.zip › Source data-Uncropped immunoblots.pdf]

Fig. 1d

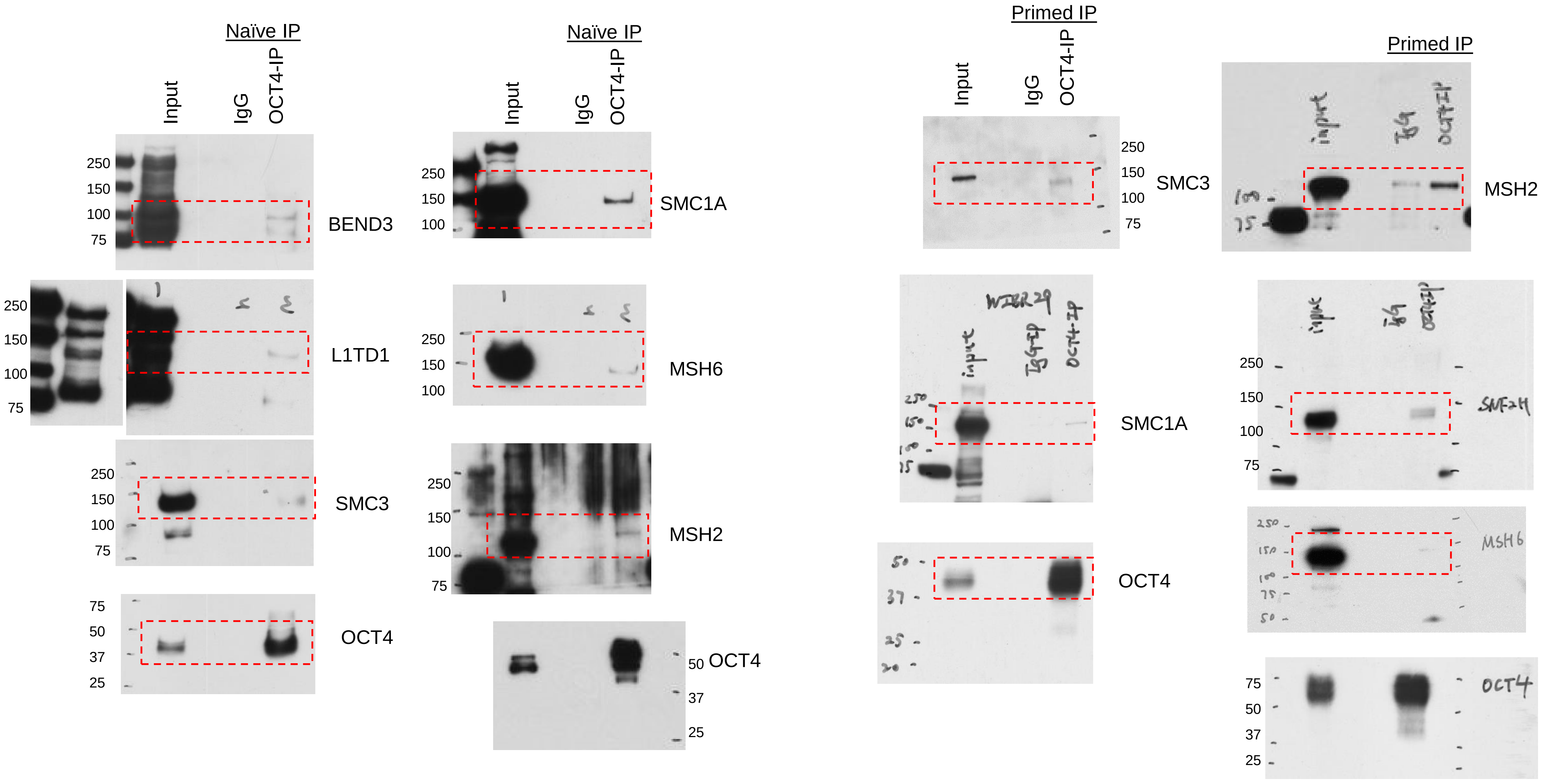

Naïve IP
Input
IgG
OCT4-IP
250
150
100
75
BEND3
L1TD1
SMC3
OCT4
50
37
25
SMC1A
MSH6
MSH2
Primed IP
MSH2
SMC1A
SNF2H
MSH6
OCT4

Fig. 1e

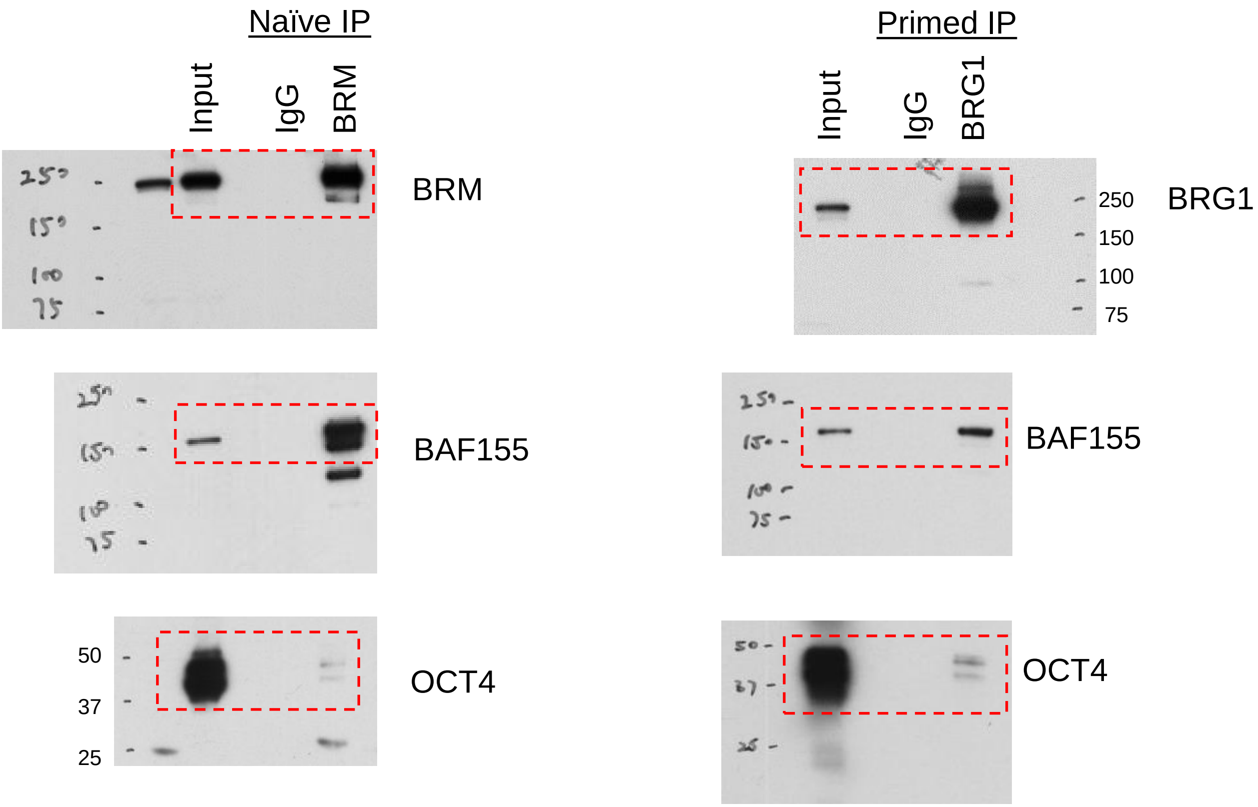

Naïve IP
Input
IgG
BRM
BRM
BAF155
OCT4
50
37
25
Primed IP
Input
IgG
BRG1
BRG1
250
150
100
75
BAF155
OCT4

Fig. 2b

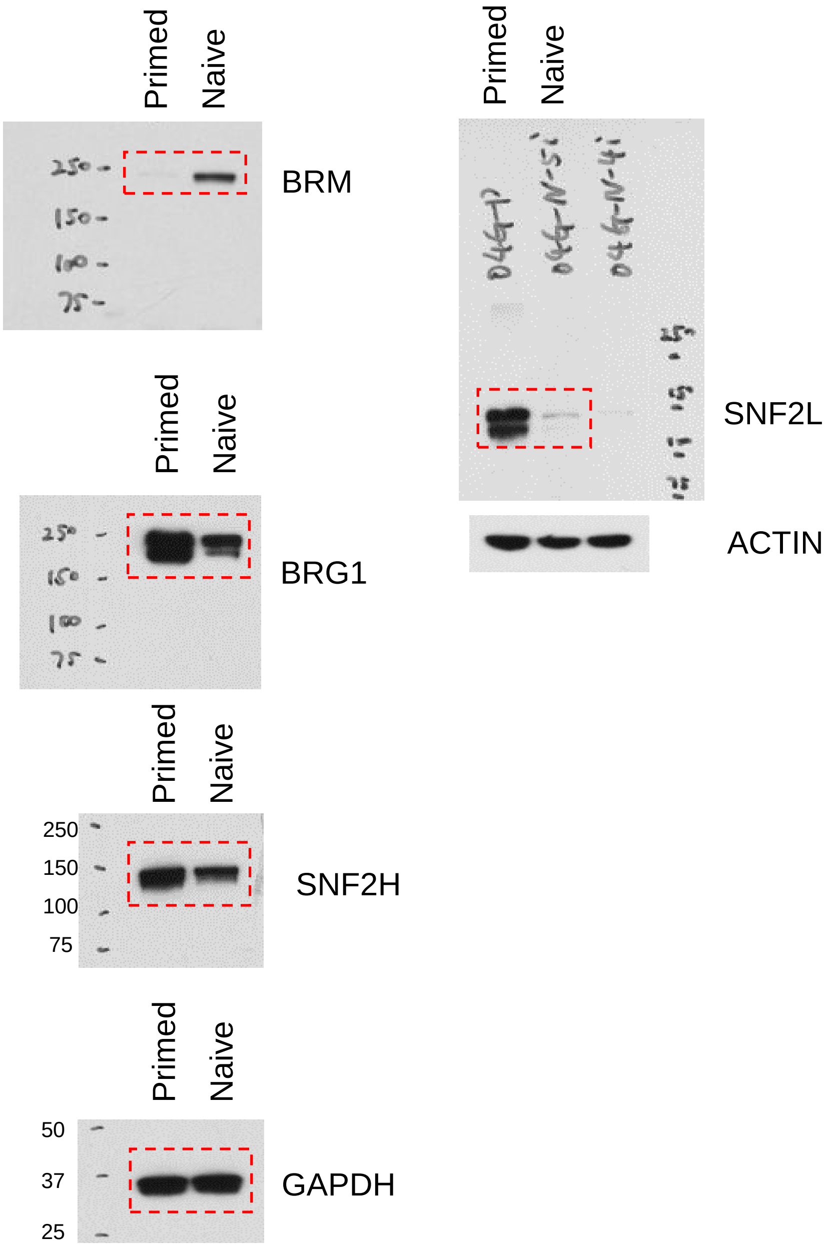
Primed
Naive
BRM
Primed
Naive
BRG1
Primed
Naive
250
150
100
75
SNF2H
Primed
Naive
50
37
25
GAPDH
Primed
Naive
SNF2L
ACTIN

Fig. 6g

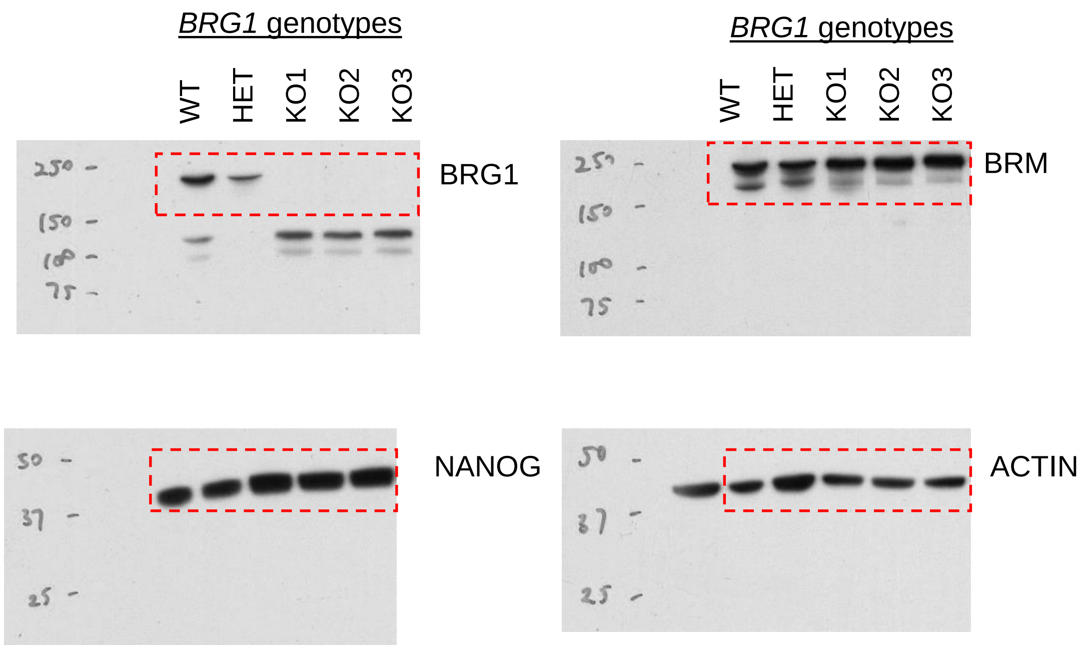
BRG1 genotypes
WT
HET
KO1
KO2
KO3
250
150
100
75
BRG1
BRG1 genotypes
WT
HET
KO1
KO2
KO3
250
150
100
75
BRM
50
37
25
NANOG
50
37
25
ACTIN

Fig. 7b

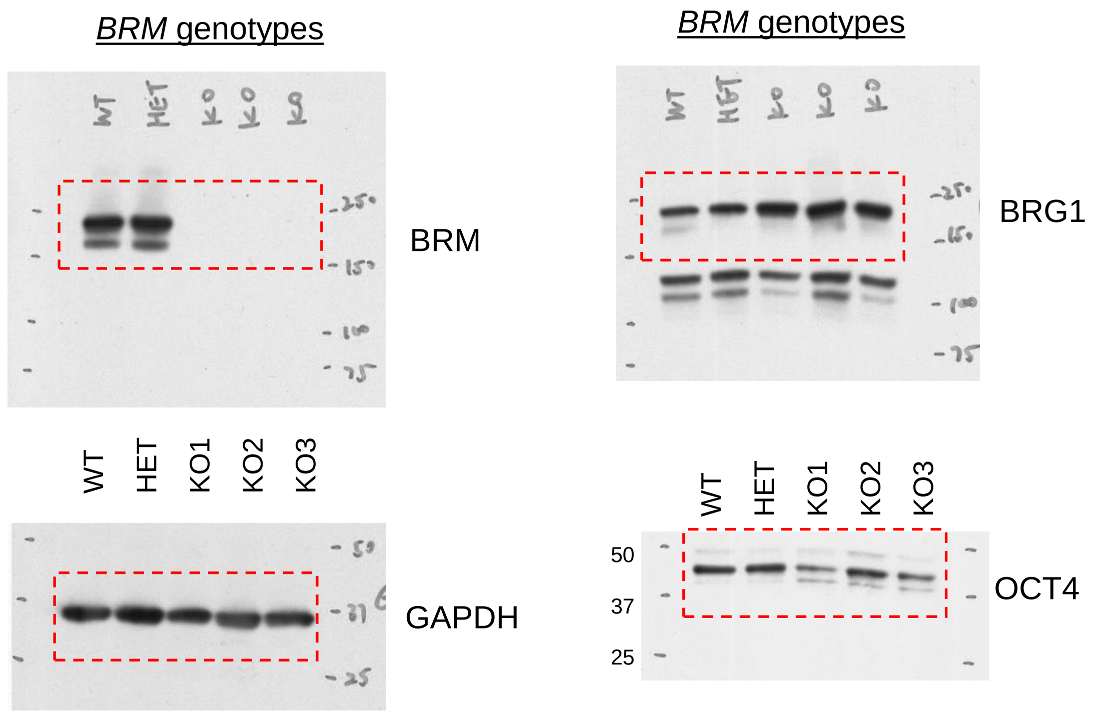

BRM genotypes
BRM
WT
HET
KO1
KO2
KO3
GAPDH
BRM genotypes
BRG1
WT
HET
KO1
KO2
KO3
50
37
25
OCT4

Suppl. Fig. 1f

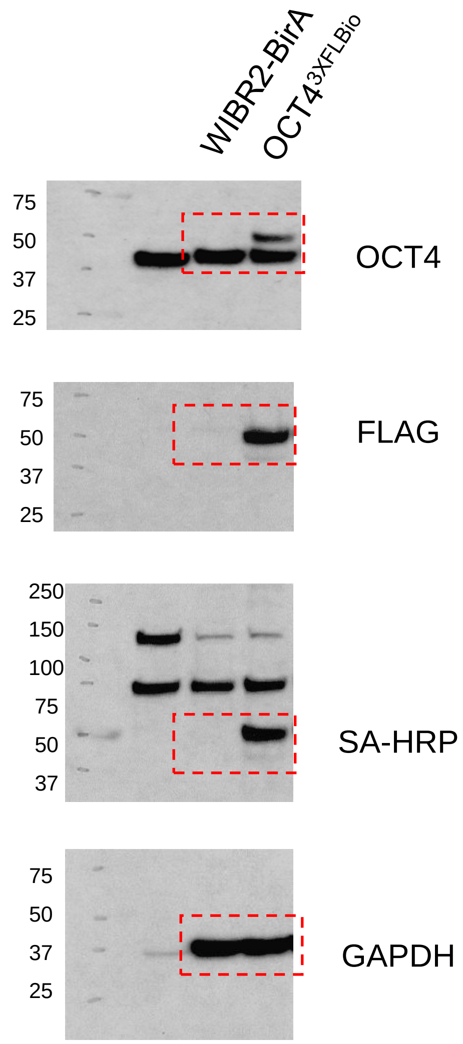

WIBR2-BirA
OCT4 3XFLBio
75
50
37
25
OCT4
75
50
37
25
FLAG
250
150
100
75
50
37
SA-HRP
75
50
37
25
GAPDH

Suppl. Fig. 1k

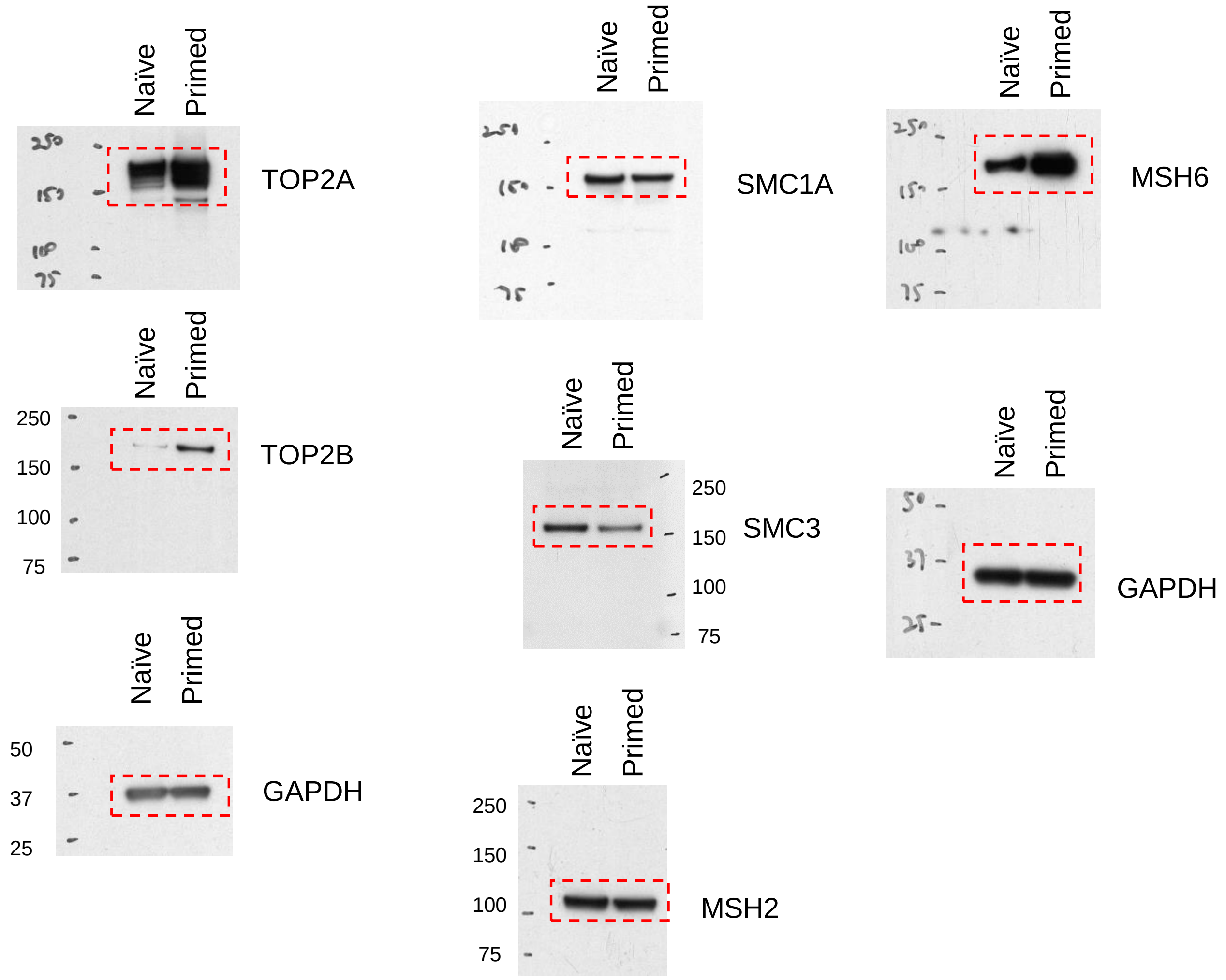
Naïve
Primed
TOP2A
SMC1A
MSH6
TOP2B
250
150
100
75
SMC3
GAPDH
50
37
25
MSH2

Suppl. Fig. 2b

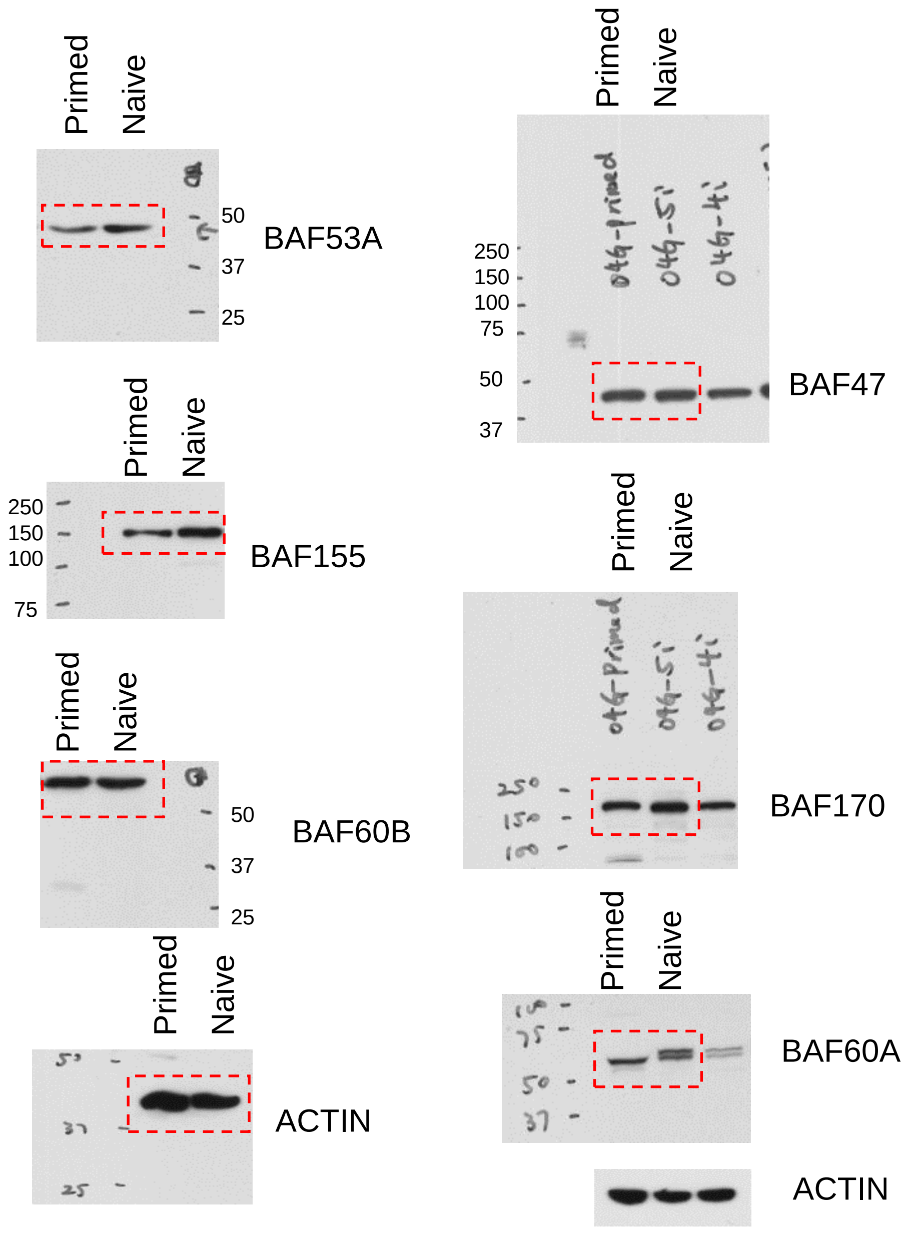

Primed
Naive
50
37
25
BAF53A
250
150
100
75
BAF155
BAF60B
ACTIN
BAF47
BAF170
BAF60A

Suppl. Fig. 2c

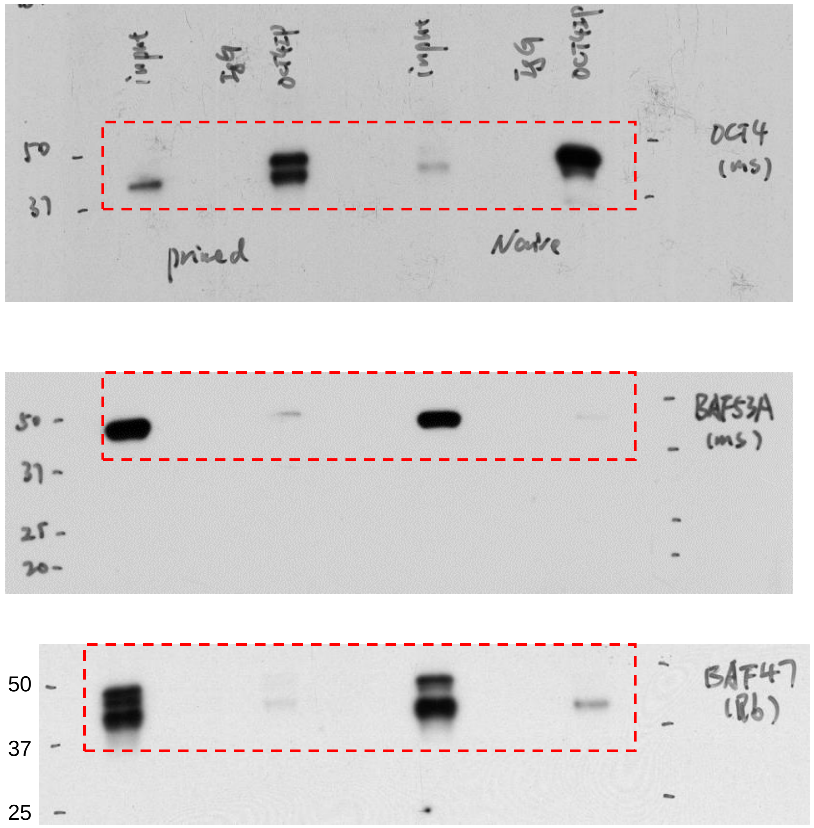

Suppl. Fig. 2d

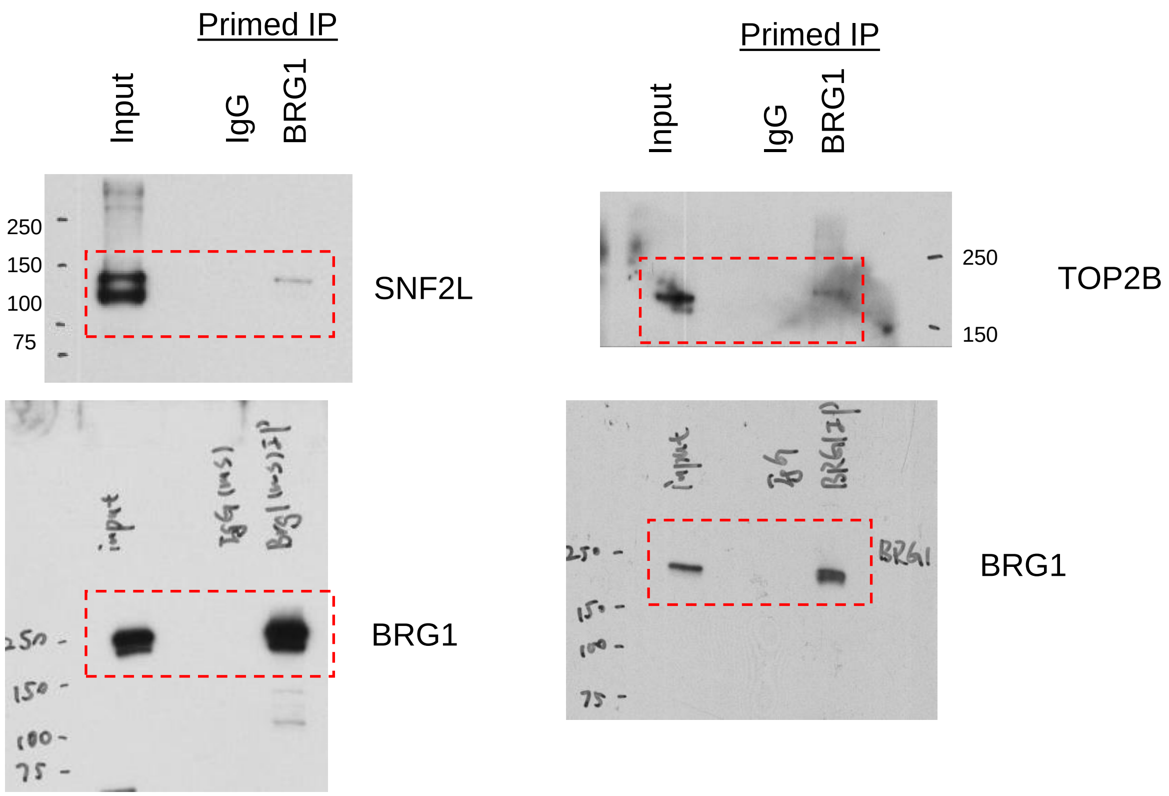

Primed IP
Input
IgG
BRG1
250
150
100
75
SNF2L
BRG1
Primed IP
Input
IgG
BRG1
250
150
TOP2B
BRG1

Suppl. Fig. 4b

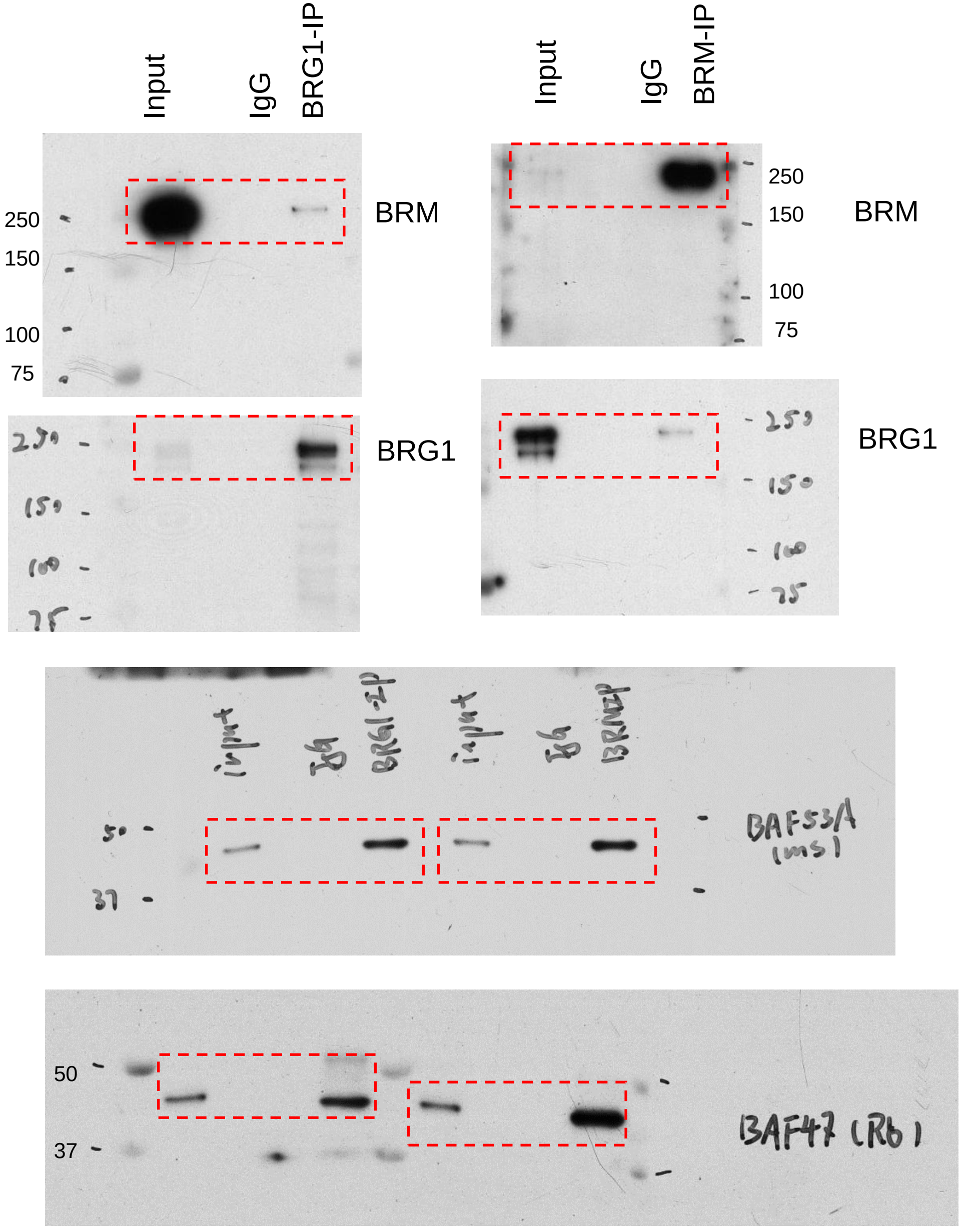
Input
IgG
BRG1-IP
250
150
100
75
BRM
BRG1
Input
IgG
BRM-IP
BRM
BRG1
50
37
BAF47 (Rb)

Suppl. Fig. 4c

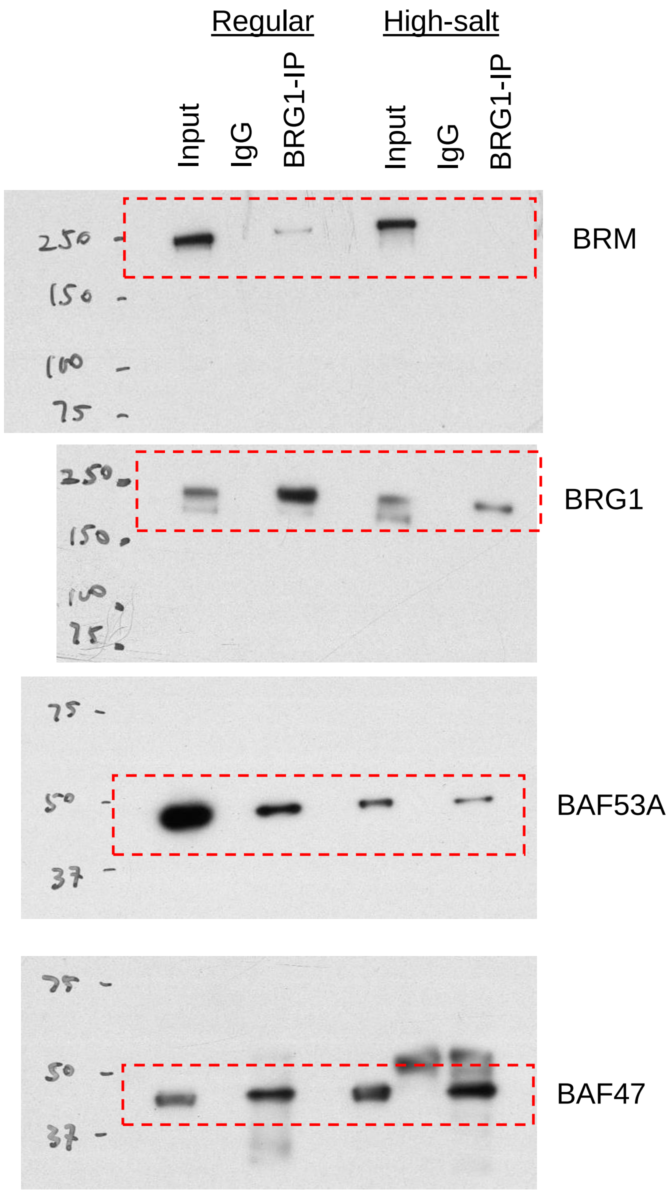
Regular
High-salt
Input
IgG
BRG1-IP
Input
IgG
BRG1-IP
250
150
100
75
BRM
250
150
100
75
BRG1
75
50
37
BAF53A
75
50
37
BAF47

Suppl. Fig. 4d

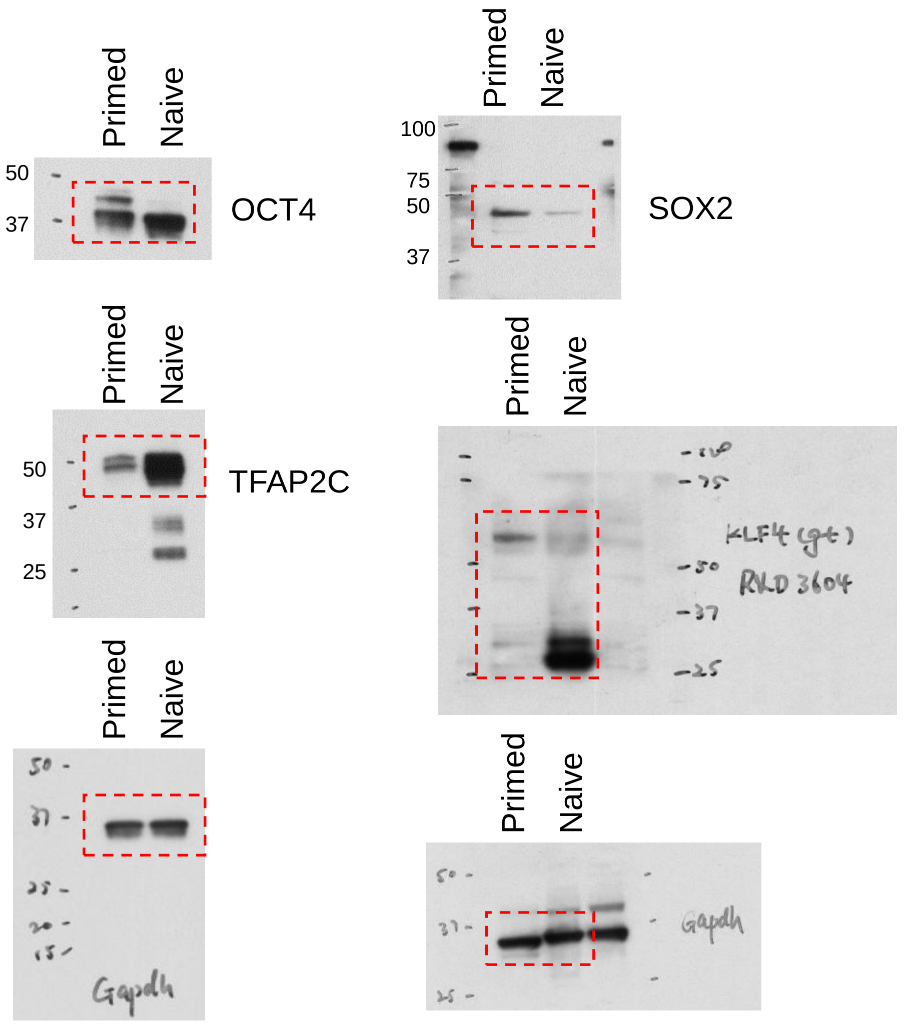
Primed
Naive
50
37
OCT4
Primed
Naive
100
75
50
37
SOX2
Primed
Naive
50
37
25
TFAP2C
Primed
Naive
KLF4 (gt)
RVD 3604
Primed
Naive
Gapdh
Primed
Naive
Gapdh

Suppl. Fig. 6b

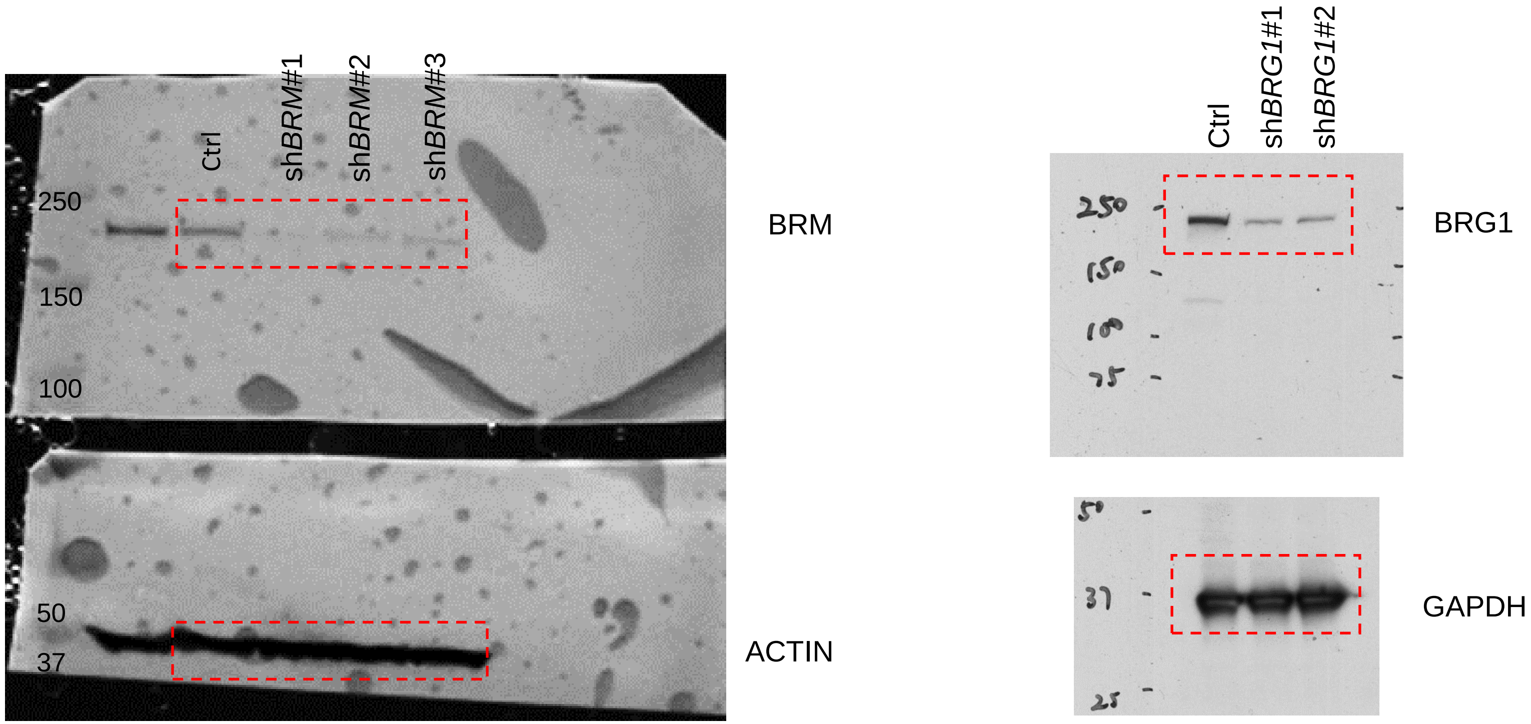

Ctrl
shBRM#1
shBRM#2
shBRM#3
250
150
100
50
37
BRM
ACTIN
Ctrl
shBRG1#1
shBRG1#2
250
150
100
75
BRG1
50
37
25
GAPDH
